# Impact and cost-effectiveness of short-course tuberculosis preventive treatment for household contacts and people with HIV in 29 high-incidence countries: a modelling analysis

**DOI:** 10.1016/S2214-109X(23)00251-6

**Published:** 2023-07-18

**Authors:** Theresa Ryckman, Jeff Weiser, Makaita Gombe, Karin Turner, Priyanka Soni, Dessislava Tarlton, Nargiza Mazhidova, Gavin Churchyard, Richard E Chaisson, David W Dowdy

**Affiliations:** a Center for Tuberculosis Research, Division of Infectious Diseases, Johns Hopkins University School of Medicine, Baltimore, MD, USA; b The Aurum Institute, Parktown, Johannesburg, South Africa; c Unitaid, Geneva, Switzerland; d Department of Epidemiology, Johns Hopkins Bloomberg School of Public Health, Baltimore, MD, USA

## Abstract

**Background:**

Guidelines and implementation of tuberculosis preventive treatment (TPT) vary by age and HIV status. Specifically, TPT is strongly recommended for people living with HIV/AIDS (PLWHA) and household contacts younger than 5 years but only conditionally recommended for older contacts. Cost remains a major barrier to implementation. The aim of this study was to evaluate the cost-effectiveness of TPT for household contacts and PLWHA.

**Methods:**

We developed a state-transition model to simulate short-course TPT for household contacts and PLWHA in 29 high-incidence countries based on data from previous studies and public databases. Our primary outcome was the incremental cost-effectiveness ratio, expressed as incremental discounted costs (2020 US$, including contact investigation costs) per incremental discounted disability-adjusted life year (DALY) averted, compared with a scenario without any TPT or contact investigation. We propagated uncertainty in all model parameters using probabilistic sensitivity analysis and also evaluated the sensitivity of results to the screening algorithm used to rule out active disease, the choice of TPT regimen, the modelling time horizon, assumptions about TPT coverage, antiretroviral therapy discontinuation, and secondary transmission.

**Findings:**

Between 2023 and 2035, scaling up TPT prevented 0·9 (95% uncertainty interval 0·4–1·6) people from developing tuberculosis and 0·13 (0·05–0·27) tuberculosis deaths per 100 PLWHA, at an incremental cost of $15 (9–21) per PLWHA. For household contacts, TPT (with contact investigation) averted 1·1 (0·5–2·0) cases and 0·7 (0·4–1·0) deaths per 100 contacts, at a cost of $21 (17–25) per contact. Cost-effectiveness was most favourable for household contacts younger than 5 years ($22 per DALY averted) and contacts aged 5–14 years ($104 per DALY averted) but also fell within conservative cost-effectiveness thresholds in many countries for PLWHA ($722 per DALY averted) and adult contacts ($309 per DALY averted). Costs per DALY averted tended to be lower when compared with a scenario with contact investigation but no TPT. The cost-effectiveness of TPT was not substantially altered in sensitivity analyses, except that TPT was more favourable in analysis that considered a longer time horizon or included secondary transmission benefits.

**Interpretation:**

In many high-incidence countries, short-course TPT is likely to be cost-effective for PLWHA and household contacts of all ages, regardless of whether contact investigation is already in place. Failing to implement tuberculosis contact investigation and TPT will incur a large burden of avertable illness and mortality in the next decade.

**Funding:**

Unitaid.

## Introduction

Tuberculosis preventive treatment (TPT), including short-course options such as 12 weeks of isoniazid and rifapentine (3HP) and 1 month of daily isoniazid and rifapentine (1HP), is effective at preventing progression to active (ie, infectious or symptomatic) disease.^1^ People living with HIV/AIDS (PLWHA) and household contacts of people diagnosed with tuberculosis are two populations at high-risk that could benefit from TPT.^2^, ^3^ TPT is effective in preventing tuberculosis in these populations,^4^, ^5^ and modelling analyses have suggested that delivering TPT to PLWHA and household contacts younger than 5 years will be cost-effective in both high-income^6^, ^7^, ^8^ and low-income and middle-income countries (LMICs).^9^, ^10^, ^11^, ^12^ As such, WHO recommends TPT for PLWHA and household contacts younger than 5 years. Studies in high-income settings have also shown TPT to be cost-effective for household contacts older than 5 years,^6^, ^7^, ^8^ but evidence from LMIC settings is scarce. Partially as a result, WHO has introduced recommendations for TPT provision to both PLWHA and household contacts, but recommendations for older contacts remain conditional.^13^ Reflecting this global hesitancy, 5-year global targets for treating PLWHA with TPT (2018–22) were met in 3 years,^14^ but only 29% of the target for household contacts younger than 5 years and less than 2% of that for adolescent and adult household contacts were met in that time.

**Research in context**

**Evidence before this study**

Previous studies in countries with low tuberculosis incidence have shown the cost-effectiveness of tuberculosis preventive treatment (TPT) for household contacts and people living with HIV/AIDS (PLWHA). In some high-tuberculosis-incidence settings, short-course TPT has also been shown to have potential to substantially reduce tuberculosis incidence and deaths and be cost-effective for household contacts younger than five years and PLWHA. However, evidence on the impact and cost-effectiveness of TPT for older contacts, and across a broader range of high-incidence settings, is scarce. Normative guidelines for TPT in this population are thus only conditional. We searched PubMed for studies of TPT cost-effectiveness in household contacts with the search terms (“economic evaluation” OR “cost-effectiveness”) AND (“TB” or “tuberculosis”) AND (“preventive therapy” OR “preventative therapy” OR “preventive treatment” OR “preventative treatment” OR “TPT” OR “3HP” OR “1HP” OR “IPT” OR “6H”) AND (“contact” OR “contacts”) from database inception to Aug 25, 2022. No language restrictions were applied to the search. This search yielded 25 publications, including nine cost-effectiveness studies: two assessing TPT for child contacts only; three assessing TPT for contacts in high-resource, low-incidence settings; one assessing TPT for contacts of people with multidrug-resistant tuberculosis only; two assessing interventions to improve diagnosis of tuberculosis infection or TPT initiation among contacts; and one that assessed scaling up TPT for both people with HIV and contacts together against current TPT levels. No studies were identified that provided estimates of the incremental cost-effectiveness of TPT among contacts older than 5 years in high-incidence settings.

**Added value of this study**

To our knowledge, this is the first study to estimate the impact and cost-effectiveness of TPT for household contacts older than 5 years, including in high-incidence settings. This study is also the first to provide comparable evidence on the cost-effectiveness of short-course TPT for PLWHA and household contacts in three age groups (<5 years, 5–14 years, and ≥15 years old), using consistent methods for all four populations. Additionally, we provide evidence that could be used to inform decisions in 29 high-incidence countries, and show how impact and cost-effectiveness vary across settings.

**Implications of all the available evidence**

Our findings suggest that short-course TPT is likely to be cost-effective (compared with no TPT) for contacts younger than 15 years old in almost all modelled settings, which includes countries with both high and low burdens of HIV. Short-course TPT is also projected to be cost-effective for adult contacts in 15 countries and PLWHA in seven countries, even under conservative cost-effectiveness thresholds. Domestic and external funding agencies should prioritise expansion of TPT for household contacts of all ages, not just those younger than 5 years—with particular focus on children and adolescents younger than 15 years.

The costs of scaling up a TPT programme, which include not just TPT drugs but also investigation to rule out active disease, are a major barrier to implementation of TPT guidelines and allocation of resources.^15^ However, we are not aware of any estimates of the cost-effectiveness of scaling up a TPT programme for household contacts of different ages (particularly adults) or TPT compared with no TPT for household contacts (of any ages) in high-incidence settings. We therefore aimed to estimate the cost, health impact, and cost-effectiveness of implementing TPT in four populations (PLWHA and household contacts in three age strata [<5 years, 5–14 years, and ≥15 years]) across 29 high-incidence countries.

## Methods

### Model and parameters

**Figure 1:**
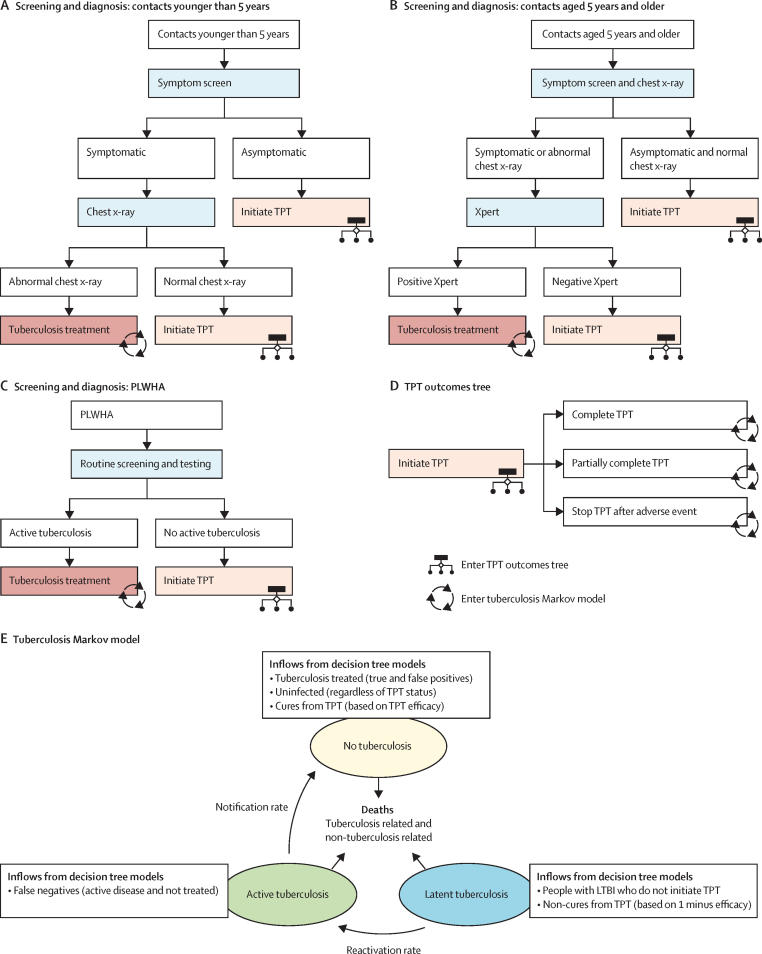
Model diagrams In A–D white boxes indicate populations and sub-populations, and coloured boxes indicate processes of contact investigation and TPT. (A) contact investigation for children younger than 5 years is assumed to screen for tuberculosis based on symptom screening only. Those with positive symptom screening are tested using chest x-ray, and those with chest x-rays suggestive of tuberculosis are initiated on tuberculosis treatment (including both true and false positives). All those presumed to not have tuberculosis disease (negative symptom screening or normal chest x-ray; including both true and false negatives) are eligible to initiate TPT. (B) Contacts aged 5 years and older (including contacts aged 5–14 years and contacts aged 15 years and older) are screened using both symptom screening and chest x-ray. Those with positive symptom screening or abnormal chest x-ray, or both, are tested for tuberculosis disease using Xpert MTB/RIF. Contacts who are Xpert positive (including both true and false positives) are initiated on tuberculosis treatment, and contacts who are Xpert negative and those with both negative symptom screening and normal chest x-ray are eligible to initiate TPT (including both true and false negatives). For household contacts of all ages with tuberculosis infection who are improperly treated for tuberculosis disease (false positives), treatment is assumed to cure their infections (see panel E). Household contacts of all ages with tuberculosis disease who are improperly given TPT or not given any treatment (false negatives) continue to have active disease until detected through the health system (based on background notifications; see panel E). (C) PLWHA taking antiretroviral therapy are assumed to be screened and tested for tuberculosis on a regular basis, such that active disease is detected promptly and treatment is subsequently initiated (see panel E). PLWHA for whom tuberculosis is not detected are eligible to initiate TPT during a routine health system visit. (D) Once TPT is initiated, both household contacts and PLWHA may complete the full course (full efficacy achieved), complete a partial course (50% efficacy achieved), or discontinue TPT due to an adverse event (no efficacy achieved). Those initiating TPT with tuberculosis infection are cured of their infections based on efficacy; those initiating TPT without tuberculosis infection are unaffected (see panel E). After following the decision trees in panels A–D, cohorts enter a Markov model that tracks their tuberculosis status on an annual basis (panel E). (E) Circles indicate states and arrows indicate transitions between states. Among PLWHA, antiretroviral therapy status is also tracked in the Markov model (panel E). Additional details for the Markov model are in the appendix (pp 4–6). LTBI=latent tuberculosis infection. PLWHA=people living with HIV/AIDS. TPT=tuberculosis preventive treatment.

We developed combined decision-tree and state-transition models to simulate the delivery of short-course TPT for PLWHA and household contacts at a country level (figure 1). Our model incorporates contact screening; TPT initiation, discontinuation, adverse events, and completion; testing and subsequent treatment of active tuberculosis; antiretroviral therapy provision for PLWHA; and subsequent risks and timing of reactivation, with associated mortality risks and costs. Details on the 29 countries modelled are in the appendix (p 3). All model parameters including data sources are in the table, with additional model and parameter details in the appendix (pp 4–14). Our analysis conforms to Consolidated Health Economic Evaluation Reporting Standards for economic evaluation studies (appendix p 15).^32^

Appropriate management of household contacts of people diagnosed with pulmonary or extrapulmonary tuberculosis includes both diagnosis (and treatment) of active tuberculosis and provision of TPT once active tuberculosis is ruled out. We thus conceptualised contact investigation and TPT as a combined intervention,^15^ modelling contact investigation (including screening for and diagnosis of active pulmonary tuberculosis) and TPT delivery for household contacts based on WHO guidance (figure 1A–B).^13^ For contacts younger than 5 years, we modelled a household visit with symptom screening, in which symptomatic children would be referred to a health facility for diagnosis using chest x-ray.^13^, ^33^ Those with a chest x-ray suggestive of tuberculosis would initiate treatment for tuberculosis disease, and children with a negative symptom screening or negative chest x-ray would be eligible to initiate TPT. For contacts aged 5 years and older, we modelled a household visit with symptom screening as well as facility-based chest x-ray; in a sensitivity analysis, we included symptom screening only.^13^ Those with a positive symptom screening or positive chest x-ray would be tested for tuberculosis using an Xpert MTB/RIF assay, with a positive Xpert test triggering tuberculosis treatment and a negative Xpert test (or negative symptom screening or negative chest x-ray) triggering TPT eligibility. For PLWHA on antiretroviral therapy, we assumed that eligibility for either TPT or tuberculosis treatment would be evaluated through routine health visits, without any added screening costs (figure 1C).

For simulated individuals deemed eligible for TPT, we modelled the cascade of TPT initiation and completion (figure 1D). We modelled a 74% probability of TPT initiation for eligible individuals;^16^ for PLWHA, we assumed that TPT initiation could occur during the same visit as initial evaluation, whereas for household contacts, we included the cost of one additional health facility visit. Those with undiagnosed active tuberculosis or without tuberculosis infection were assumed to obtain no benefit from TPT (while still incurring TPT-related costs); those with tuberculosis infection who completed TPT were assumed to be cured of existing infections according to TPT efficacy estimates. Completion of a partial course of TPT was assumed to provide half the benefit of a full course, but those who stopped TPT after having adverse reactions incurred additional costs of laboratory tests and, in severe cases, hospitalisation, and derived no benefits.

After modelling the outcomes of screening, diagnosis, and tuberculosis treatment or TPT, we used a state-transition Markov model (figure 1E) to track tuberculosis status from 2023 through 2035. This 13-year horizon matched global tuberculosis targets; longer horizons were explored in sensitivity analysis. Prevalence of tuberculosis infection was based on country-varying estimates for PLWHA and age-varying estimates for household contacts.^2^, ^34^ We represented tuberculosis status via three states (no infection, latent infection, and active disease) and modelled a risk of progression to active disease that differed by age (for household contacts), time since infection, and HIV status. For purposes of simplicity, we did not include any overlap in our modelled PLWHA and household contact populations. For PLWHA, we modelled transitions on and off antiretroviral therapy and associated costs; our base case analysis assumed frequent transitions on and off antiretroviral therapy, with sensitivity analysis considering infrequent transitions. We defined frequent transitions as 15% transition off in the first year on antiretroviral therapy and 8% transition off in subsequent years, and 72% annual probability of transitioning back on once transitioned off; and infrequent transitions as 10% transition off in the first year and no further transitions on or off in subsequent years. For household contacts and PLWHA off antiretroviral therapy, cure from active disease occurred according to country-specific and age-specific case detection and treatment success ratios, and tuberculosis disease was assumed to be detected through routine screening among PLWHA on antiretroviral therapy.

### Policy scenarios

We modelled scenarios in which coverage (ie, the percent of the TPT-eligible population entering the contact investigation and prevention or treatment cascade) increased from 0% in year 0 (2022) to 90% in year 10 (2032; remaining at 90% through 2035), for each of the four target populations across 29 selected countries that had begun to procure short-course TPT as of March, 2022 (appendix p 3). For purposes of transparency, linear annual increases of 9 percentage points per year in coverage (from 0–90%) were assumed. These assumptions resulted in cumulative numbers of people initiated on TPT in each country that are consistent with the UN High Level Meeting targets for TPT provision.^35^

We compared outcomes to a no-TPT scenario; for household contacts, we also assumed no contact investigation (ie, that tuberculosis among household contacts would only be detected and treated according to age-specific and country-specific background notification rates). Our primary analysis considered 3HP as the TPT regimen being scaled up, but in sensitivity analysis we considered 1HP. 1HP was assumed to have higher completion,^4^ fewer monitoring visits, and higher drug costs. Lower 3HP coverage (50%) and more required monitoring visits were considered in separate sensitivity analyses. For household contacts, we also considered comparisons of: (1) contact investigation (no TPT) versus no intervention, and (2) contact investigation plus TPT versus contact investigation alone.

### Outcomes and sensitivity analyses

We estimated cumulative tuberculosis incidence, tuberculosis deaths, disability-adjusted life years (DALYs), and costs (expressed in 2020 US$) from 2023 through 2035. Cases, deaths, and costs are presented in per person terms—calculated as total cases, deaths, or costs divided by the total population size of contacts of a given age or PLWHA. Our primary outcome was the incremental cost-effectiveness ratio (ICER), defined as the mean incremental discounted costs of a 3HP programme (according to a health systems perspective) divided by the mean incremental discounted DALYs averted, compared with no TPT. We calculated these outcomes by sampling 50 000 sets of parameter values independently from their corresponding distributions (table; appendix p 10) using Latin hypercube sampling. We ran the model with each parameter set for each country and calculated means and 95% uncertainty intervals (UIs; 2·5th and 97·5th percentiles) of modelled outcomes across simulations. We used a 3% annual discount rate for both costs and DALYs, consistent with standard cost-effectiveness guidelines.^36^ ICERs were compared to country-specific cost-effectiveness thresholds,^37^ with per-capita gross national income and US$1000 per DALY averted used as secondary references (appendix p 14).

**Table:** Model parameters

	PLWHA*	Household contacts*	Sources*
TPT efficacy†	87% (75–95)	87% (75–95)	Martinez et al (2020)^5^
TPT acceptance†	73·5% (68·7–88·3)	73·5% (68·7–88·3)	Mandalakas et al (2021)^16^
3HP completion	85% (77–91)	85% (77–91)	Yanes-Lane et al (2021);^4^ Villarino et al (2015)^17^
Discontinuation due to adverse reaction: not requiring hospitalisation†	5·1% (4·2–6·1)	Age <15 years 1·5% (0·6–2·8); age ≥15 years 5·1% (4·2–6·1)	Villarino et al (2015)^17^
Discontinuation due to adverse reaction: requiring hospitalisation†	1·0% (0·1–2·7)	Age <15 years 0·2% (<0·1%–0·8); age ≥15 years 0·5% (0·2–0·8)	Villarino et al (2015)^17^
LTBI prevalence	6–57%‡	35·5% (30·3–41·1)§	Fox et al (2013)^2^
Annual tuberculosis progression¶	0·33% (0·08–0·78) to 8·3% (3·8–14·2)	0·08% (0·06–0·09) to 10% (4·3–20·9)	Martinez et al (2020)^5^
Tuberculosis case-fatality ratio	On ART 10% (5–15); off ART 25% (16–35)	Ages <5 years and treated 1·9% (0·5–7·1); age ≥5 years and treated 0·8% (0·3–2·1); age <5 years and untreated 43·6% (36·8–50·6); age ≥5 years and untreated 14·9% (11·5–19·1)	Jenkins et al (2017)^18^
Background notification rate‖	On ART (100%); off ART (varies‡)	Varies‡	WHO (2020)^14^
Probability of treatment success	Varies‡	Varies‡	WHO (2020)^14^
Active tuberculosis prevalence	NA**	Age <5 years 10% (5·0–18·9); age 5–14 years 8·4% (2·8–22·6); age ≥15 years 3·2% (2·0–5·3)	Fox et al (2013)^2^
Prevalence of positive screen	NA**	Age <5 years 34·5% (31·5–37·6); age 5–14 years 28% (18–38); age ≥15 years 18% (11–27)	Martinez et al (2018)^19^
Chest x-ray sensitivity	NA**	Age <5 years 87% (75–93)	Vonasek et al (2021)^20^
Chest x-ray specificity	NA**	Age <5 years 99% (68–100)	Vonasek et al (2021)^20^
Xpert sensitivity	NA**	Age 5–14 years 73% (64·7–79·6); age ≥15 years 91% (86·2–94·7)	Zifodya et al (2021)^21^
Xpert specificity	NA**	Age 5–14 years 98·0% (95·8–98·5); age ≥15 years 98·0% (95·8–98·5)	Zifodya et al (2021) ^21^
Household contact investigation	NA**	$1·7–48·8‡	Chikovani et al (2021)^22^
3HP drugs full course	$13·5 and one outpatient visit	Age <5 years $6 and two outpatient visits; age 5–14 years $12 and two outpatient visits; age ≥15 years $13·5 and two outpatient visits	Assumed††
1HP drugs full course	$22 and one outpatient visit	All ages: $22 and two outpatient visits	Assumed††
TPT overhead and administrative costs	10% of drug costs	10% of drug costs	Arinaminpathy et al (2015)^23^
Chest x-ray	NA**	$2·8–20·0‡	Chikovani et al (2021)^22^
Xpert MTB/RIF	NA**	$23·3 (20–31)	Pooran et al (2019)^24^
Laboratory testing for adverse drug reaction	$18·20 (13·3–23·1)	$18·20 (13·3–23·1)	South Africa National Health Laboratory Service (2018)^25^
Outpatient visit	$0·4–7·7‡	$0·4–7·7‡	WHO (2011)^26^
Inpatient visit	$1·6–82·3‡	$1·6–82·3‡	WHO (2011)^26^
Tuberculosis treatment, full course‡‡	$130–4745‡	$130–4745‡	WHO (2020)^14^
ART per year ‡‡	$95·7 and four outpatient visits per year, 10% overheads	NA	Ravelo (2021)^27^
All-cause mortality	Year 1 on ART 5·8% (2·8–8·9); subsequent years 2·2% (0·7–3·7); off ART: 6·5% (2·1–11·0)	Varies by country and age	Bernard et al (2018)^28^
Tuberculosis disability weight	0·408 (0·274–0·549)	0·333 (0·224–0·454)	Global Burden of Disease Collaborative Network (2019)^29^
Non-tuberculosis disability weight	On ART 0·087 (0·057–0·123); off ART 0·274 (0·186–0·371)	0·000	Global Burden of Disease Collaborative Network (2019)^29^
Annual decline in tuberculosis incidence	NA**	2%	Assumed
Annual decline in HIV incidence§§	0–11%‡	NA	UNAIDS AIDSinfo (2022)^30^
ART coverage§§	81%	NA	Assumed§§
Transition off ART—ie, from loss to follow-up	Year 1 on ART 15% (12–18); subsequent years 8% (5–11)	NA	Bernard et al (2018)^28^
Transition back on ART if LTFU	72% (63–80)	NA	Calibrated¶¶
Delay between HIV infection and ART initiation	2 years	NA	Assumed
Life expectancy	27 years (25–29)	Varies by country and age	Mills et al (2011)^31^

Data are mean (95% CI). 1HP=1 month of daily isoniazid and rifapentine. 3HP=3 months of weekly isoniazid and rifapentine. ART=antiretroviral therapy. LTBI=latent tuberculosis infection. LTFU=loss to follow up. NA=not applicable. PLWHA=people living with HIV/AIDS. TPT=tuberculosis preventive treatment.

* In many cases, more than one reference was used to develop a parameter estimate; one representative reference is provided in this table. For most parameters, the full set of references and the distributions used in probabilistic sensitivity analysis are shown in the appendix (pp 10–11). Cost parameter references and distributions are described in the appendix (pp 7–9).

† Parameter was assumed to be the same for both 3HP and 1HP.

‡ Estimates vary by country (appendix pp 6–9).

§ For household contacts, we focused on LTBI prevalence that was from recent infection.

¶ Annual progression was modelled among those with LTBI only and varies by age, ART status (for PLWHA), and time since infection (for household contacts; appendix p 12).

‖ Background notification rates (ie, probability of case detection absent TPT or contact investigation; also known as the treatment coverage ratio) varied by age and country (appendix pp 6–9).

** These parameters are relevant for household contacts only and are not required in the model of PLWHA.

†† Drug price estimates from Aurum Institute. At the time of the analysis, the price per full adult course of 3HP was US$14·25. For PLWHA, the TPT initiation and completion visit coincide with routine quarterly visits.

‡‡ The costs of tuberculosis treatment and ART shown include all cost components.

§§ Annual decline in HIV incidence was based on declines over the past 5 years. ART coverage was assumed to achieve the global 90-90-90 targets for the HIV care cascade.

¶¶ More details in the appendix (p 6).

To benchmark programme affordability, we compared total programme costs to countries' reported tuberculosis budgets.^14^ To evaluate the potential impact of TPT in averting ongoing transmission, we performed scenario analyses assuming that each person with active tuberculosis caused 0·5 or one additional person to develop tuberculosis a mean of 2 years later.^38^ We used linear regression meta-modelling^39^ to conduct one-way sensitivity analysis describing the influence of each model parameter on projected cost-effectiveness. We also assessed the sensitivity of cost-effectiveness to the price of 3HP and the range of TB treatment costs across the 29 countries (table). The country-specific tuberculosis treatment costs are in the appendix (p 9).

### Role of the funding source

PS, DT, and NM are employees of Unitaid and they contributed to interpretation of the results and suggesting revisions to the manuscript. The funder of the study had no role in study design, data collection, data analysis, or writing of the report.

## Results

**Figure 2:**
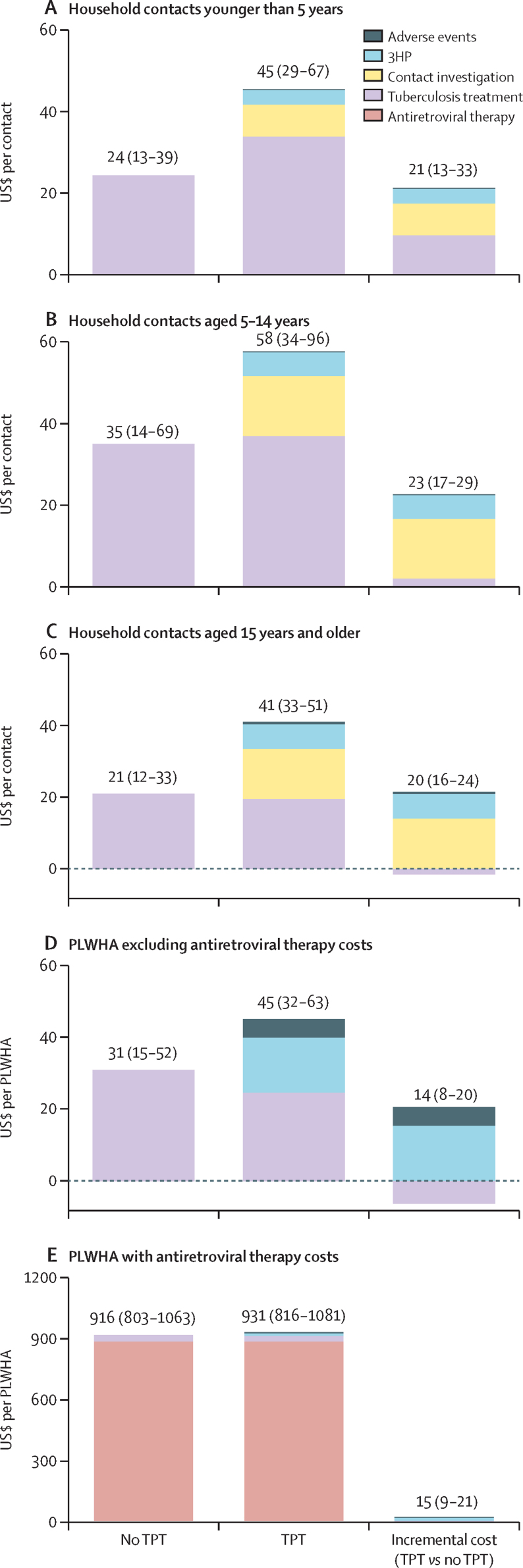
Mean per-person costs of scaling up short-course TPT for PLWHA and household contacts across 29 countries (A–D) Mean tuberculosis-related costs per household contact (of each age group) and per eligible PLWHA in 29 countries of tuberculosis treatment in a population not initiating TPT, in a population for whom 3HP is scaled up through 2035, and the mean per-person cost differences between the two scenarios (incremental cost). Costs include tuberculosis treatment, contact investigations, 3HP, and adverse events. (E) The same costs as panel D, but includes antiretroviral therapy costs for PLWHA. Text labels indicate mean costs, with 95% UI (2·5th and 97·5th percentiles) from the 50 000 probabilistic sensitivity analysis samples in brackets. Contact investigation and ruling out active tuberculosis comprised 39% of incremental costs among children younger than 5 years and 65–70% of incremental costs among older contacts. 3HP drugs and delivery comprised 18% (contacts younger than 5 years), 26% (contacts aged 5–14 years), and 35% (contacts aged 15 years and older) of incremental costs. Additional tuberculosis treatment accounted for 42% (95% UI 12–66) of total incremental costs to contacts younger than 5 years, and tuberculosis treatment savings were expected among contacts aged 15 years and older. 95% UI=95% uncertainty intervals. 3HP=12 weeks of isoniazid and rifapentine. PLWHA=people living with HIV/AIDS. TPT=tuberculosis preventive treatment.

For every 100 household contacts in the 29 modelled countries, without any TPT or contact investigations, 7·2 (95% UI 4·6–10·6) contacts were expected to develop tuberculosis disease and be treated from 2023 through 2035. Accounting also for contacts who would not develop tuberculosis, the average cost of tuberculosis treatment was $24 (95% UI 13–39) per contact younger than 5 years, $35 (14–69) per contact aged 5–14 years, and $21 (12–33) per contact aged 15 years and older (figure 2A–C).

If contact investigation and short-course TPT were scaled up simultaneously to initiate 102 million (95% UI 95 million–108 million) contacts on 3HP through 2035 (appendix p 22), projected costs nearly doubled (incremental cost of $21 [17–25] per contact) to $45 (29–67) per contact younger than 5 years, $58 (34–96) per contact aged 5–14 years, and $41 (33–51) per contact aged 15 years and older. If TPT were instead incorporated into existing contact investigation programmes, incremental costs (which no longer include the costs of contact investigation itself) were $1 (cost-saving to $3) per contact younger than 5 years, $3 (1–5) per contact aged 5–14 years, and $4 (1–7) per contact aged 15 years and older (appendix p 23).

For every 100 PLWHA in the 29 modelled countries, 4·3 (95% UI 2·1–7·3) per 100 were expected to develop tuberculosis disease and be treated (assuming no TPT), at an estimated mean tuberculosis treatment cost of $31 (15–52) per PLWHA (figure 2D–E, accounting also for PLWHA who would not develop tuberculosis). Scaling up short-course TPT to cover 14·8 million PLWHA was expected to cost $15 (9–21) per PLWHA more than no TPT, almost entirely due to TPT drug costs.

Incremental costs varied by country, primarily reflecting different background notification levels and tuberculosis treatment costs (appendix pp 24–28). The annual mean incremental costs of a short-course TPT programme for PLWHA ranged from cost-saving to 6% of countries' annual tuberculosis budgets (appendix p 29). Costs of contact investigation and short-course TPT ranged from 1–7% of budgets for contacts younger than 5 years, 1–17% for contacts aged 5–14 years, and 2–35% for contacts aged 15 and older.

**Figure 3:**
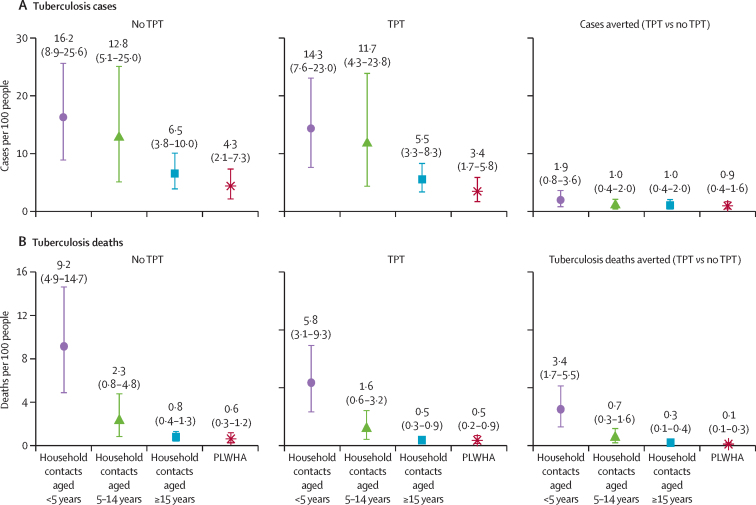
Mean per-person health impact of scaling up 3HP for PLWHA and household contacts in 29 countries (A) Mean active tuberculosis cases per 100 eligible people (ie, per household contact of each of three age groups, displayed in years, or per PLWHA) across 29 countries, in populations not initiating TPT, populations for whom TPT is scaled up through 2035, and the incremental cases averted between the two scenarios (cases averted). (B) The same cumulative outcomes and differences by scenario and population as in A, but for tuberculosis deaths rather than cases. Note the difference in scale on the y axis for the two outcomes. 3HP=12 weeks of isoniazid and rifapentine. PLWHA=people living with HIV/AIDS. TPT=tuberculosis preventive treatment.

For every 100 household contacts across the 29 modelled countries, 8·8 (95% UI 5·8–12·5) contacts were expected to develop tuberculosis disease, and 1·9 (1·3–2·8) contacts were expected to die of tuberculosis through 2035; 19% (10–31) of incidence and 48% (30–67) of deaths were among children younger than 5 years (figure 3). Scaling up contact investigation and TPT through 2035 could yield a 13% (7–19) cumulative reduction in the number of people developing tuberculosis (1·1 [0·5–2·0] cases averted per 100 contacts) and a 35% (32–38) cumulative reduction in deaths (0·7 [0·4–1·0] deaths averted per 100 contacts). Among children younger than 5 years, the impact of contact investigation on mortality was so substantial that a programme with both contact investigation and short-course TPT was estimated to avert more deaths than cases. Contact investigation alone was responsible for 68% of all deaths averted (appendix p 23).

Among PLWHA, scaling up short-course TPT was projected to cumulatively reduce incidence by 21% (95% UI 14–28) and tuberculosis mortality by 22% (15–29) compared with no TPT (0·9 [95% UI 0·4–1·6] cases and 0·13 [0·05–0·27] deaths averted per 100 PLWHA). Relative reductions in incidence among both household contacts and PLWHA were similar across the 29 modelled countries; mortality reductions varied with background case detection ratios (appendix pp 30–37).

**Figure 4:**
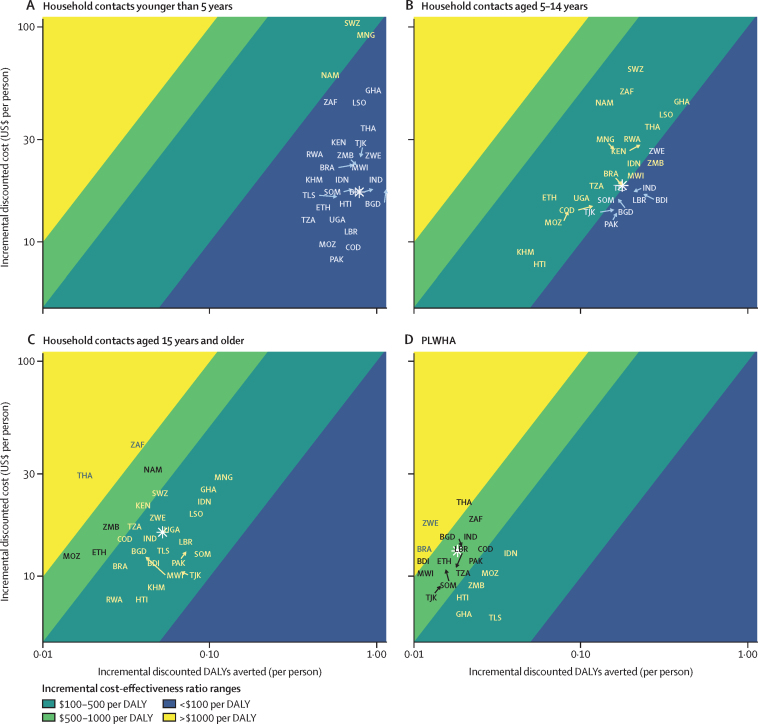
Cost-effectiveness of 3HP for household contacts and PLWHA in 29 countries Each marker represents a country-specific estimate of discounted incremental DALYs averted per person (ie, per household contact or PLWHA) from implementing 3HP (x axis) and corresponding discounted incremental costs (y axis), compared with a scenario of no TPT for household contacts younger than 5 years (A), household contacts aged 5–14 years (B), household contacts aged 15 years and older (C), and PLWHA (D). Both x and y axes are on a log scale. Countries are labelled by their 3-digit ISO codes (positions of country code labels have been jittered to avoid overlaps), and population-weighted means across the 29 countries are designated via a white star. Shaded areas indicate incremental cost-effectiveness ratios. For example, in B, the plot indicates that the incremental cost-effectiveness ratio of 3HP for contacts aged 5–14 years in ZAF is between $100 and $500 per DALY averted, and in D, the plot indicates that the incremental cost-effectiveness ratio of tuberculosis preventive treatment for PLWHA in ZAF is between $500 and $1000 per DALY averted. Cambodia, Eswatini, Mongolia, and Namibia are omitted from panel D because scaling up 3HP for PLWHA in these countries was estimated to be cost saving; there were no countries for which the intervention was cost saving in the other analyses presented in A–C. Values and cost-effectiveness thresholds for each country are in the appendix (pp 56–58). 3HP=12 weeks of isoniazid and rifapentine. DALY=disability-adjusted life year. PLWHA=people living with HIV/AIDS. BDI=Burundi. BGD=Bangladesh. BRA=Brazil. COD=DR Congo. ETH=Ethiopia. GHA=Ghana. HTI=Haiti. IDN=Indonesia. IND=India. KEN=Kenya. KHM=Cambodia. LBR=Liberia. LSO=Lesotho. MNG=Mongolia. MOZ=Mozambique. MWI=Malawi. NAM=Namibia. PAK=Pakistan. RWA=Rwanda. SOM=Somalia. SWZ=Eswatini. THA=Thailand. TJK=Tajikistan. TLS=Timor-Leste. TZA=Tanzania. UGA=Uganda. ZAF=South Africa. ZMB=Zambia. ZWE=Zimbabwe.

The cost-effectiveness of short-course TPT plus contact investigation had a mean of $22 per DALY averted for contacts younger than 5 years (range across the 29 countries: $14–154), $104 per DALY averted for contacts aged 5–14 years (range $70–320), $309 per DALY averted for contacts aged 15 years and older (range $155–1637), and $722 per DALY averted for PLWHA (range: cost-saving to $2866; figure 4). In 25 of the 29 countries, short-course TPT for contacts younger than 5 years and contacts aged 5–14 years were the two most cost-effective strategies. In 19 countries, the incremental cost-effectiveness of short-course TPT for household contacts aged 15 years and older was more favourable than short-course TPT for PLWHA (appendix pp 38–39). When added to an existing contact investigation programme (ie, excluding the incremental costs of contact investigation), short-course TPT was generally somewhat more cost-effective, with mean ICERs of $4 (contacts younger than 5 years), $70 (contacts aged 5–14 years), and $164 per DALY averted (contacts aged 15 years and older; appendix p 40).

The estimated cost-effectiveness of short-course TPT for household contacts younger than 15 years fell below country-specific thresholds in all countries (all countries apart from DR Congo for contacts aged 5–14 years; appendix pp 56–58) and most parameter samples (appendix pp 54–55). For contacts aged 15 years and older and PLWHA, the cost-effectiveness of short-course TPT was more varied but remained below thresholds in 15 countries (contacts aged 15 years and older) and seven countries (PLWHA).

Variation across countries was driven primarily by three model parameters: background notification rates, outpatient visit cost, and tuberculosis treatment costs (appendix pp 41–51). Reduced TPT prices could improve cost-effectiveness, particularly for PLWHA (appendix p 52).

Short-course TPT cost-effectiveness was substantially more favourable (often crossing cost-effectiveness thresholds) if averted secondary transmission was incorporated or a longer time horizon were assumed. Most other sensitivity analyses in most countries did not affect whether short-course TPT was considered cost-effective (appendix pp 59–74).

## Discussion

This simulation model of 29 high-incidence, early short-course TPT adopter countries suggests that implementing 3HP for household contacts younger than 15 years is cost-effective in almost all countries (except for DR Congo for contacts aged 5–14 years), even under conservative cost-effectiveness thresholds. This finding held regardless of whether a contact investigation programme was already in place. Short-course TPT was also cost-effective for adult contacts in 15 of 29 countries and for PLWHA in seven countries, although these conclusions vary depending on the cost-effectiveness threshold used. In our assessment of cost-effectiveness, we used conservative country-specific thresholds. Under more liberal thresholds (per-capita gross national income or $1000 per DALY averted), short-course TPT for PLWHA and adult contacts was cost-effective in 72–97% of countries.

If TPT and contact investigation are not scaled up for these populations, nearly 850 000 preventable tuberculosis deaths are projected to occur through 2035 (compared with 1·1 million total tuberculosis deaths estimated to occur in the 29 countries in 2021), 700 000 of which will be among household contacts younger than 15 years old. Short-course TPT (with contact investigation) among PLWHA and household contacts—especially children and adolescents—is therefore both a cost-effective intervention and an urgent health priority. Short-course TPT was almost always more cost-effective for younger contacts because children face higher risks of tuberculosis progression and mortality and are less likely to be detected through the health system without contact investigation.

Despite its cost-effectiveness, the cost of scaling up TPT (with contact investigation) to such a large population (over 80 million contacts) is substantial, representing more than 50% of the total tuberculosis budget in some countries such as DR Congo and Pakistan. External funding, with an explicit plan for bridging to domestic support as tuberculosis burden declines, will be needed to support this activity in many LMICs. Such a funding plan can help make TPT scaleup feasible in the short term and sustainable in the long term.

Importantly, we assumed an incremental cost of contact investigation for all contacts, but reducing the costs of contact investigation visits and screening tests could improve cost-effectiveness. To the extent that contact investigations can be conducted more efficiently (eg, screening multiple households or household members on a single visit) or costs can be shared (by leveraging community health worker platforms or combining visits with other screening activities), the incremental cost of contact investigation (and thus of a TPT programme) could be substantially reduced, particularly in settings with larger households. Designing innovative, efficient approaches to contact investigation should be considered a high priority for TPT implementation research.

Our analysis assumed household visits; other types of contact investigations, such as those done at health facilities, would probably reduce costs but at the expense of coverage. The price of diagnosis might also decrease with the development of new technologies, such as point-of-care and near point-of-care tools, which could improve cost-effectiveness. TPT might also be more cost-effective in subgroups of contacts at high risk, such as those with undernourishment or diabetes who are more likely to progress from infection to disease.

Although we used 3HP as our reference TPT regimen, use of the most effective and cost-effective regimen (3HP, 1HP, four months of rifampin, or other emerging regimens) is an important consideration. A previous analysis concluded that 3HP for PLWHA was likely to be cost-effective compared with 9 months of daily isoniazid (IPT) if the price of rifapentine were reduced to its current level.^40^ If the price of 3HP could be further lowered close to the price of IPT, which is estimated to have lower completion, carry higher risks of isoniazid resistance acquisition, and result in higher mortality risks,^4^, ^41^ 3HP would be cost-effective in virtually all settings and may even be cost saving for PLWHA.^42^ Therefore, although we found that minor fluctuations in the price of 3HP are unlikely to affect cost-effectiveness, price reductions remain a high priority. Under one set of assumptions regarding cost, efficacy, and adherence, we found that 1HP could be similarly (but slightly less) cost-effective, consistent with other analyses that have addressed this question in more detail.^43^ Future analyses comparing different regimens should consider the potential for price reductions, subsequent evidence on comparative efficacy, emerging programmatic evidence on adherence, and feasibility of implementation in varied health systems.

We chose to simulate an intervention in which TPT was provided to all contacts once active disease was ruled out, regardless of infection status. Requiring a positive test for tuberculosis infection would reduce the frequency of adverse events but would also avert fewer cases^44^ and substantially increase costs. Given differential risks of tuberculosis disease, TPT acceptance, adverse events, and contact patterns by age, future studies should consider whether an age cutoff might be appropriate for requiring infection testing, or initiating TPT at all, among older contacts.

Strengths of our analysis include comparison across multiple countries and consideration of the costs of contact investigation. We expect these findings to generalise to other countries with high burdens of tuberculosis, because the countries we modelled represent a range of income levels, geographical regions, and HIV and tuberculosis epidemics. Our results are broadly consistent, though somewhat less optimistic, when compared with other published studies evaluating the cost-effectiveness for PLWHA.^9^, ^10^, ^42^ Our more pessimistic projections might reflect the focus of most published literature on IPT (priced lower than 3HP), our assumption that TPT would be given to all PLWHA regardless of infection status, and our incorporation of time-varying tuberculosis progression risk that is dependent on antiretroviral therapy status.

Given the uncertainties regarding the annual risk of tuberculosis infection and the contribution of PLWHA (or contacts) to transmission at the population level, we conservatively did not incorporate reductions in transmission in our primary analysis. Consideration of secondary transmission improved cost-effectiveness. To the extent that adults are more likely to transmit tuberculosis than children, the cost-effectiveness of TPT for older contacts might be most improved by considering secondary transmission.

Other limitations include uncertainty regarding the long-term trajectories of tuberculosis and HIV incidence and the risk of reinfection. There is limited evidence on appropriate cost-effectiveness thresholds for LMICs;^45^ as such, we compared ICERs to multiple references and tried to avoid reliance on explicit thresholds, but this approach limits precision. Available evidence indicates that health opportunity costs in these settings tend to be lower than gross national income per capita,^37^, ^45^ consistent with our methodology. Given domestic financing constraints, the willingness-to-pay of funding partners is also an important consideration. Additionally, we did not consider cost implications outside of the health sector (eg, patient-borne transportation costs and lost wages), which could accrue to those taking TPT and those with tuberculosis disease. Finally, we did not model drug resistance. Currently, TPT is recommended only for high-risk contacts of people with multidrug resistant (MDR) tuberculosis.^13^ In this context, TPT might cost more, but savings from averting MDR-tuberculosis could also be higher. Ongoing trials will provide additional evidence that can inform cost-effectiveness estimates of TPT for MDR-tuberculosis contacts.

In summary, short-course TPT is likely to be cost-effective not only for child contacts younger than 5 years, but also for older child and adult contacts in most countries. Failure to deliver TPT to these populations in the 29 high-incidence countries considered here alone could result in nearly 850 000 avertable tuberculosis deaths through 2035. Guidelines should consider stronger recommendations for providing TPT to household contacts of all ages (especially those aged 5–14 years), and both domestic and external funding agencies should prioritise urgent expansion of TPT to these populations if ambitious targets for ending tuberculosis are to be met.

## Data sharing

All data used in this study are from publicly available sources that are listed in the table and cited in the references.

## Declaration of interests

REC reports funding from Unitaid through a grant subcontract to Johns Hopkins (2017-20-IMPAACT4TB). All other authors declare no competing interests.
