# Supplementary material for: Impact and cost-effectiveness of short-course tuberculosis preventive treatment for household contacts and people with HIV in 29 high-incidence countries: a modelling analysis
Source: Lancet Glob Health. 2023 Jul 18;11(8):e1205–16. doi: 10.1016/S2214-109X(23)00251-6 (PMC10369017; doi:10.1016/S2214-109X(23)00251-6)
Supplement: Supplementary appendix [file mmc1.pdf]

THE LANCET
Global Health

Supplementary appendix

This appendix formed part of the original submission and has been peer reviewed. We post it as supplied by the authors.

Supplement to: Ryckman T, Weiser J, Gombe M, et al. Impact and cost-effectiveness of short-course tuberculosis preventive treatment for household contacts and people with HIV in 29 high-incidence countries: a modelling analysis. *Lancet Glob Health* 2023; **11:** e1205–16.

Supplementary Material for "Impact and cost-effectiveness of short-course TB preventive treatment for household contacts and people with HIV in 29 high-incidence countries: a modeling analysis"

SUPPLEMENTARY METHODS 3

Modeled Countries 3
Appendix Table 1: Additional details on the 29 modeled countries *3*
Model Details 4
Household Contacts *4*
People living with HIV/AIDS *5*
Parameter Details 6
Background probabilities of detection and treatment *6*
Country-Specific Costs *7*
Appendix Table 2: Country-specific model parameters *9*
Appendix Table 3: Parameter distributions *10*
Annual TB Reactivation *12*
Appendix Table 4: Annual progression probabilities *12*
Willingness-to-Pay Threshold Details 14
Appendix Table 5: CHEERS checklist for economic evaluation studies 15
Supplementary Methods References 17

SUPPLEMENTARY RESULTS 22

Appendix Figure 1: Projected TPT initiation among household contacts and PLWHA in 29 countries 22
Appendix Figure 2: Cumulative ten-year costs and health impact of scaling up household contact investigations with or without TPT in 29 countries 23
Appendix Table 6: Costs of scaling up a 3HP program for household contacts under five years 24
Appendix Table 7: Costs of scaling up a 3HP program for household contacts five to fourteen years 25
Appendix Table 8: Costs of scaling up a 3HP program for household contacts fifteen years and above 26
Appendix Table 9: Costs of scaling up a 3HP program for people living with HIV/AIDS 27
Appendix Table 10: Costs of scaling up a 3HP program for people living with HIV/AIDS, excluding ART 28
Appendix Figure 3: Average annual cost of a 3HP program, as a share of countries' annual TB budgets 29
Appendix Table 11: Total projected TB cases with and without 3HP 30
Appendix Table 12: Projected TB cases per 100 contacts and per 100 people living with HIV/AIDS, with and without 3HP 32
Appendix Table 13: Total projected TB deaths with and without 3HP 34
Appendix Table 14: Projected TB deaths per 100 contacts and per 100 people living with HIV/AIDS, with and without 3HP 36
Appendix Figure 4A: Comparative cost-effectiveness of TPT for different target populations 38
Appendix Figure 4B: Comparative cost-effectiveness of TPT for different target populations 39
Appendix Figure 5: Cost-effectiveness of contact investigation with 3HP versus contact investigation without 3HP for household contacts in 29 countries 40
Summary of sensitivity of results to individual model parameters 41
Appendix Figure 6: Sensitivity of cost-effectiveness results for contacts < 5 years to parameter uncertainty 42
Appendix Figure 7: Sensitivity of cost-effectiveness results for contacts aged 5-14 years to parameter uncertainty 44
Appendix Figure 8: Sensitivity of cost-effectiveness results for contacts aged 15 years and above to parameter uncertainty 46
Appendix Figure 9: Sensitivity of cost-effectiveness results for people living with HIV/AIDS to parameter uncertainty 48

Appendix Figure 10: Sensitivity of cost-effectiveness results to the cost of TB treatment....................................50
Appendix Figure 11: Sensitivity of cost-effectiveness results to the price of 3HP 52
Appendix Figure 12: Cost-effectiveness of TPT by population across cost-effectiveness thresholds 54
Appendix Table 15: Cost-effectiveness of 3HP for household contacts and PLWHA in 29 countries – totals 56
Appendix Table 16: Cost-effectiveness of 3HP for household contacts and PLWHA in 29 countries – per person 57
Appendix Table 17: Cost-effectiveness of 3HP for household contacts and PLWHA in 29 countries...................58
Summary of sensitivity analysis results 59
Appendix Table 18: Sensitivity analysis results for contacts < 5 years..60
Appendix Table 19: Sensitivity analysis results for contacts aged 5-14 years..64
Appendix Table 20: Sensitivity analysis results for contacts over 15 years...69
Appendix Table 21: Sensitivity analysis results for people living with HIV/AIDS...74

SUPPLEMENTARY METHODS

Modeled Countries

Appendix Table 1 shows additional details on the TB and HIV epidemics in the 29 modeled countries.

Appendix Table 1: Additional details on the 29 modeled countries

Country	3-digit ISO code (used in figures)	Included in the WHO's 30 high TB burden countries?	Included in the WHO's 30 TB-HIV high-burden countries?	Estimated annual TB incidence per 100,000 population, 2021	Estimated HIV prevalence, 2021 (ages 15-49, %)	Estimated percent of incident TB among people with HIV, 2021 (%)
Burundi	BDI	No	No	100	0.9	5.7
Bangladesh	BGD	Yes	No	221	< 0.1	0.2
Brazil	BRA	Yes	Yes	48	0.6	12
Congo DRC	COD	Yes	Yes	318	0.7	8
Ethiopia	ETH	Yes	Yes	119	0.8	5.2
Ghana	GHA	No	No	136	1.7	14
Haiti	HTI	No	No	159	1.8	14
Indonesia	IDN	Yes	Yes	354	0.3	2.3
India	IND	Yes	Yes	210	0.2	1.8
Kenya	KEN	Yes	Yes	251	4.0	25
Cambodia	KHM	No*	No	288	0.6	1.7
Liberia	LBR	Yes	Yes	308	1.1	11
Lesotho	LSO	Yes	Yes	614	20.9	65
Mongolia	MNG	Yes	No	428	< 0.1	0.1
Mozambique	MOZ	Yes	Yes	361	**	25
Malawi	MWI	No	Yes	132	7.7	45
Namibia	NAM	Yes	Yes	457	11.8	32
Pakistan	PAK	Yes	No	264	0.2	2.4
Rwanda	RWA	No	No	56	2.3	16
Somalia	SOM	No	No	250	< 0.1	0.9
Eswatini	SWZ	No	Yes	348	27.9	60
Thailand	THA	Yes	Yes	143	1.0	8.8
Tajikistan	TJK	No	No	88	0.2	2.9
Timor-Leste	TLS	No	No	486	0.2	1.0
Tanzania	TZA	Yes	Yes	208	4.5	18
Uganda	UGA	Yes	Yes	199	5.2	32
South Africa	ZAF	Yes	Yes	513	18.3	53
Zambia	ZMB	Yes	Yes	307	10.8	34
Zimbabwe	ZWE	No*	Yes	190	11.6	62

Data from WHO[1] and UNAIDS[2]

*Cambodia and Zimbabwe are instead categorized as "global TB watchlist" countries.

** Recent prevalence estimates are not available from UNAIDS' database.

Model Details

Household Contacts

We modeled annual cohorts of household contacts consisting of each of three age groups (< 5, 5-14, and ≥ 15 years old); the size of each cohort was based on the estimated number of TB notifications (all forms) and the average household size (by age group) in each country. We included contacts of people diagnosed with both pulmonary and extrapulmonary TB. To estimate annual TB notifications, we assumed that TB incidence would decline by 2% annually (from estimated country-specific 2021 incidence levels) and that the percent of people with TB that are notified would remain constant (again compared to estimated 2021 country-specific treatment coverage ratios).[3] Data on household sizes and the household-age distribution came from a United Nations Population Division database made up of country census and household survey data.[4] For lack of better data, we assumed that the composition of households with TB would be similar to the average household in each country.

The proportion of each household contact cohort that has active TB disease (active), latent TB infection (LTBI), or no TB infection/disease (no TB) was based on estimates of active and latent TB prevalence from household contact studies (see main text Table 1 and *Parameter Details* section of the appendix for more details on all parameter estimates).[5]

In the *TPT* scenario, a percentage of each cohort receives a contact investigation visit. For household contacts, the percentage receiving visits increases linearly from 0% in the year before the first year of the model (2022), to 9% in the first year (2023), up to 90% in the tenth through thirteenth years (2032-35). TPT initiation was then based on the proportion of household contacts that screen/test negative for active disease (and are thus eligible for TPT) and estimates of the TPT initiation ratio among investigated household contacts that do not have active TB disease.[6] Contacts < 5 are assumed to be screened for symptoms only (main text Figure 1A). Those that screen positive are tested for active disease using chest Xray (CXR), and those with abnormal CXR are presumed to have TB disease and initiate TB treatment. Everyone else (negative symptom screen and/or normal CXR) is eligible to initiate TPT. Contacts ≥ 5 are assumed to be screened for symptoms and screened with CXR (main text Figure 1B). Those with abnormal CXR and/or positive symptom screen are tested for active disease using Xpert, and those with a positive Xpert are presumed to have TB disease and initiate TB treatment. Everyone else (negative symptom screen and normal CXR, or negative Xpert) is eligible to initiate TPT.

The proportions of household contacts that screen positive/negative and test positive/negative are based on symptom screening, CXR, and Xpert sensitivity and specificity[7–9] (which vary by age), prevalence survey data[10] (on the proportions of populations that screen positive on either CXR or symptom screen), contact investigation data[11–13] (on the proportions of child contacts that screen positive on symptom screen) and the proportions of each cohort that have active TB, LTBI, or no TB from household contact studies[5] (discussed above). Costs are incurred per person screened (contact investigation cost and, for contacts ≥ 5, CXR costs) and per person tested (CXR costs for contacts < 5, Xpert costs for contacts ≥ 5).

Contacts that screen and test positive (including true and false positives) initiate treatment (we assume no dropout among symptomatic contacts). Both true positive and false positives incur the costs of treatment; true positives who do not fail treatment (based on country-specific treatment success ratios, see *Parameter Details* section) are assumed to be cured of TB disease (transition to "no TB" state), false positives with LTBI are assumed to clear their TB infections (transition to "no TB" state), and false positives without LTBI receive no benefit from treatment. Country-specific treatment costs were based on country-specific WHO data on drug-susceptible TB treatment unit costs, but were varied widely in a focused one-way sensitivity analysis.[14] A proportion of contacts that screen negative or test negative (including true and false negatives) initiate TPT.[6] False negatives (i.e., active TB that remains undetected) continue to have TB disease regardless of whether they are initiated on TPT, as do true positives that fail treatment, and are only treated based on age-specific background notification rates in each country (see *Parameter Details* section). Everyone who initiates TPT incurs the full cost of a TPT regimen, regardless of completion or benefits. A proportion of true negatives with LTBI that initiate TPT clear their infections; that proportion is based on TPT efficacy[15,16] and their TPT completion. True negatives without LTBI receive no benefits from TPT.

The proportion of TPT initiators who complete TPT was based on estimates of TPT completion[17–21] and the frequency of adverse events (e.g., hepatotoxicity, hypersensitivity) that result in TPT discontinuation[21–23] (main text Figure 1D). We assumed that all modeled toxicity events result in TPT discontinuation (with no benefits from TPT) and incur the costs of laboratory tests[24] and an outpatient visit[25]; severe toxicity events additionally incur the cost of three inpatient bed days.[25] We assumed that those with no toxicity events that initiate but do not complete TPT have half the probability of clearing their infections as those that complete TPT (i.e., 50% relative efficacy).

In the *No TPT* scenario, no contact investigations or TPT initiations were assumed to occur, and active TB could only be cured based on country- and age-specific background notification ratios multiplied by country- and age-specific treatment success ratios (see *Parameter Details* section). Each cycle, active TB that is not detected (or is detected but not cured/does not complete treatment) remains active.

After modeling contact investigation, TPT initiation and completion (and resulting cures), and active TB treatment from contact investigation (and resulting cures), cohorts enter a Markov state-transition model that tracks their TB status annually (main text Figure 1E). Each annual timestep, percentages of each cohort can be notified and treated for active disease (active transition to no TB; incurring TB treatment costs), progress from LTBI to active disease that is subsequently notified and cured (LTBI transition to no TB; incurring TB treatment costs) or remains unnotified (LTBI transition to active), die of TB (if active) or non-TB (any state) causes, or remain in their current state. Progression from LTBI to active disease depends on age and time since entering the model (i.e., for most contacts, time since infection) (see *Parameter Details* section).[15,26,27] Treatment of active TB depends on background age-specific notification and successful treatment ratios (see *Parameter Details* section). TB mortality depends on age and treatment status.[28]

People living with HIV/AIDS

We modeled both people living with HIV/AIDS (PLWHA) that are newly enrolled on ART (new) and PLWHA that have already been established on ART but have not yet initiated TPT (established). The size of the new and established PLWHA populations are estimated based on current numbers of adult PLWHA on ART, estimated HIV incidence, and reported data on the numbers of PLWHA that have already initiated TPT. Country-specific data on the size of the adult PLWHA population enrolled on ART in 2021 were obtained from UNAIDS.[29] We added to this estimates of HIV incidence from 2019-21, assuming a 2 year delay between incidence and ART enrollment and assuming that, going forward, countries meet the targets of 90% of PLWHA knowing their HIV status and 90% of those that know their status enrolling in care (81% overall ART coverage). For 2022 and beyond, we estimated HIV incidence by calculating the average increase or decline in HIV incidence from the past five years, based on UNAIDS estimates, and assuming this trend continues for the next ten years. These calculations yielded estimates of the size of the previously-enrolled PLWHA population in 2023 and the size of the new PLWHA population in 2023-35. To estimate the size of the established (but not yet initiated on TPT) population in 2023, we subtracted out the total number of PLWHA enrolled on TPT in each country from 2015 onward, based on data reported to the WHO[3], assuming an annual mortality probability of 3·7%[30] (to account for PLWHA that enrolled in TPT but subsequently died and thus should not be subtracted out of the total number of PLWHA still alive in 2023). This adjustment yielded estimates of the size of established PLWHA population in 2023.

In 2023, we initiated the model with these two populations (new and established). In subsequent years (2024-35), the size of the new PLWHA population is also based on the above calculations. The size of the established population that could be eligible to initiate TPT in subsequent years was estimated directly from modeled output for the previous year (details in subsequent paragraphs).

Each annual timestep, a percentage of both the new and established PLWHA populations are offered TPT. Similar to household contacts, in the *TPT* scenario, this percentage increases linearly from 0% in the year before the first year of the model (2022), to 9% in the first year (2023), up to 90% in the final four years (2032-35). In the *No TPT* scenario, TPT coverage is fixed at 0% for all years. We assumed that TB disease among PLWHA will be detected and treated (and subsequently cured) through screening at routine HIV-related health facility visits, and that TPT can be initiated during one of these visits for any PLWHA that do not have active disease (Figure 1C). Because active disease screening and TPT initiation are assumed to be done through routine care and thus incur no additional costs,

we did not explicitly model a TPT initiation/acceptance probability (that would allow us to back-calculate the number of PLWHA offered TPT based on each coverage level as we did for household contacts) (i.e., we acknowledge that there is some < 100% chance that those offered TPT initiate it, but because this does not affect costs or outcomes, we did not include it in the model).

LTBI prevalence among PLWHA was based on published country-specific LTBI estimates (see Appendix Table 2).[31] We assumed PLWHA without LTBI receive no benefit from TPT. The impact of TPT for PLWHA with LTBI depends on efficacy[16] and completion. As with household contacts, the proportion of TPT initiators who complete TPT was based on estimates of TPT completion[17–21] and the frequency of adverse events (e.g., hepatotoxicity, hypersensitivity) that result in TPT discontinuation[21–23] (main text Figure 1D). We assumed that all modeled toxicity events result in TPT discontinuation (with no benefits from TPT) and incur the costs of laboratory tests[24] and an outpatient visit[25]; severe toxicity events additionally incur the cost of 3 inpatient bed days.[25] We assumed that those with no toxicity events that initiate but do not complete TPT have half the probability of clearing their infections as those that complete TPT (i.e., 50% relative efficacy).

After modeling screening for active disease and TPT initiation and completion each year, disease progression, mortality, and ART status are tracked via a state-transition Markov model (main text Figure 1E). The probability of progression from LTBI to active disease depends on time since entering the model, ART status, and time on ART upon entering the model (see *Parameter Details* section).[16,17,26,27,32] Detection and treatment among those that progress depends on ART status; all those on ART are assumed to be detected through routine care, while only some of those not on ART are detected, based on background adult notification rates (see *Parameter Details* section). Those who are notified are cured based on country-specific probabilities of treatment success for PLHIV (based on WHO treatment outcomes data). We further assumed that background notification of PLWHA not on ART with active disease would result in them being reconnected to care (transition back onto ART). TB mortality (among those that progress) and non-TB mortality (among everyone) also depends on ART status, and non-TB mortality is also assumed to be higher for those newly initiated on ART compared to those already established.[33–38]

Transitions off ART are based on cohort studies of PLWHA[35–38]; we modeled higher ART discontinuation and loss-to-follow-up in the first year on ART than in subsequent years. Transitions back on to ART were calibrated so that the overall modeled proportion of PLWHA on ART would equal 90% (consistent with the UNAIDS target for 90% of PLWHA that know their HIV status to be on ART). Because of uncertainty in ART discontinuation and return probabilities, we also conducted a sensitivity analysis with less frequent ART-related transitions (all discontinuation occurs in the first year on ART, with a 10% discontinuation risk and no subsequent returns to care). Those on ART incur annual ART costs consisting of drugs, 2 viral load tests, 4 outpatient visits, and 10% overheads.[39]

The modeled number of PLWHA on ART that have not initiated TPT at the end of a given year then forms the eligible established population for the following year (i.e., we assume that PLWHA not on ART would not initiate TPT until they returned to care).

Parameter Details

All parameter estimates are listed in the main text (Table 1), and the *Model Details* section (above) describes how these parameters were used in the model. Country-specific parameter estimates are shown in Appendix Table 2. The distributions we sampled from for each parameter in the probabilistic sensitivity analysis are shown in Appendix Table 3. Descriptions of a few estimated parameters that were not taken directly from the literature and are not described in the previous section are included below.

Background probabilities of detection and treatment

Background notification probabilities (also known as treatment coverage ratios) by age and country were calculated by dividing reported age-specific notifications by estimated age-specific incidence, after subtracting out a share of estimated notifications that are from contact investigation from the numerator.[3] We also subtracted estimated misdiagnoses (false positives) from the numerator for children < 15 years, assuming that 19·4% of notifications

among children < 5 years are false positives and half of this (9·7%) of notifications among children aged 5-14 years are false positives.[40] We incorporated uncertainty in misdiagnoses by sampling an < 5 misdiagnosis probability parameter from a beta distribution fit to 19·4% [14·4-24·8%], accounting for uncertainty reported in the source study and widening this uncertainty to account for generalizability across settings. We incorporated uncertainty in age-specific incidence by fitting gamma distributions to incidence means and confidence intervals estimated by the WHO.[3] We incorporated uncertainty in the proportion of contact investigations that would have been diagnosed routinely anyway (if not found by contact investigation) via an uninformed beta distribution (mean 50%, 95% CI [21-79%]). We sampled from these respective distributions 50,000 times to calculate 50,000 background notification probability samples for each age group and country, thus propagating uncertainty in misdiagnoses, true TB incidence, and notifications absent any contact investigation.

We multiplied the background notification probability samples by successful treatment probability samples to estimate the background probability of being detected and successfully treated. The probability of treatment success (completion and/or documented cure) was based on data on treatment outcomes reported to the WHO for each country. We sampled from beta distributions for each country, with the alpha parameter (numerator) equal to the number of documented treatment successes among child or adult cohorts in 2019 (*newrel_014_succ* and *newrel_succ - newrel_014_succ*, respectively, in the WHO outcomes database) and denominator equal to the outcomes cohort size (*newrel_014_coh* and (*newrel_coh - newrel_014_coh*), respectively) (beta parameter equals denominator minus numerator). For countries for which treatment outcomes were not reported, we drew 50,000 samples from all the other countries' samples combined. For PLHIV, treatment success probabilities were estimated similarly (based on *tbhiv_coh* and *tbhiv_succ* in the WHO database), while background notification probabilities were assumed to be 100% for PLHIV on ART and equal to adult background notification probabilities for PLHIV not on ART.

The resulting adjusted age-specific and country-specific probabilities of detection and successful treatment are shown in Appendix Table 2.

Country-Specific Costs

Country-specific costs estimates, along with other country-specific parameters, are shown in Appendix Table 2. Additional details on how these costs were estimated are provided below.

Contact investigation and CXR costs

To estimate country-specific contact investigation and CXR costs for all 29 countries, we searched the literature for published cost data that would cover each of the WHO geographic regions represented by the countries (Africa, Western Pacific, Southeast Asia, Americas, Europe).[3] For Pakistan and Somalia (which are in the Eastern Mediterranean region), we used Southeast Asia cost data and Africa cost data, respectively. We identified region-specific estimates of the cost of conducting CXR[41–45] and the cost of household contact investigation[41,46–49] and converted the cost estimates to 2020 USD using consumer price index data from the International Monetary Fund.[50] We then estimated country-specific prices by scaling the regional prices by relative Gross National Income per capita (GNI p.c.).[51] For contact investigation we scaled costs 1:1 with GNI p.c. and for CXR we scaled 1:4 with GNI p.c. since we assumed that the majority of contact investigation costs would be determined by local wages, while evidence indicated that consumables and equipment (such as CXR machines themselves, which are typically internationally-traded commodities) made up much of CXR costs and, furthermore, we did not observe much correlation between countries' income levels and reported CXR costs.[43–45]

Costs of Xpert and laboratory tests for adverse events

We assumed Xpert and lab test costs would be similar across countries and thus did not vary them across countries in our analysis. Estimates of the cost of Xpert came from a recent multi-country economic evaluation study of Xpert in southern Africa[44], while estimates of the cost of laboratory tests for suspected hepatotoxicity came from the South Africa National Health Laboratory Service price lists (we included the costs for ALT, full blood count, urine

dipstick, urine sodium, and added 50% [0-100%] for labor and overheads).[24] We conservatively applied the cost of laboratory tests for suspected hepatotoxicity to all adverse events.

Inpatient and outpatient visit costs

Country-specific estimates of the cost per outpatient visit and the cost per inpatient bed day came from WHO CHOICE estimates.[25] We converted the estimates, which were reported in 2010 international dollars, to 2010 local currency, inflated them to 2020 local currency, and then converted them to 2020 USD.[50,51] When estimates were not reported for any particular country, we used regional estimates instead.

TB treatment costs

We used WHO estimates of TB treatment unit costs, which represent the median cost per person treated with drug-susceptible TB in a country.[14] We used drug-susceptible TB costs because, in the countries with high HIV prevalence, most new TB incidence is drug-susceptible, and because the analysis is assumed to cover household contacts of people with drug-susceptible TB only.

Drug costs

Based on estimates from our coauthors at Aurum Institute, we assumed that a full course of 3HP would cost $6 per person < 5 years old, $12 per person aged 5-14 years, and $13·5 per person aged 15 and above. We also costed an outpatient monitoring visit (and for household contacts, an initiation visit). In scenario analyses of 1HP, we assumed a full course of 1HP would cost $22 for all ages and would require an outpatient visit. To account for drug wastage, we assumed that full TPT courses would be set aside for all TPT initiators (to avoid stockouts), regardless of TPT completion.

We estimated an annual ART cost of $63 (dolutegravir, lamivudine, and tenofovir) based on recent price reports.[39] We also included the costs of two viral load tests (at $12 each) and 4 outpatient visits annually. While ART, viral load tests, and monitoring visits are likely to be borne by HIV programs, not TB programs, and are mostly unaffected by the introduction of TPT, some increases in spending are expected from keeping PLWHA on ART who would have otherwise died of TB absent TPT

We added a 10% [6-14%] markup for overheads to all drug costs, based on two published studies that captured evidence on drug cost overheads (such as transportation, insurance, and quality control).[52,53]

Appendix Table 2: Country-specific model parameters

Country	Contact investigation costs	CXR costs	Outpatient visit costs	Inpatient visit costs	TB treatment costs	LTBI prevalence (PLWHA, %)	Background treatment, ages < 5 years (%)	Background treatment, ages 5-14 years (%)	Background treatment, ages ≥ 15 years (%)	Treatment success, PLWHA (%)
Burundi	8·1 [4·3-13·1]	2·4 [1·7-3·2]	0·4 [0·0-1·4]	1·6 [0·5-3·4]	402 [304-501]	21·7 [18·3-25·3]	14·8 [6·3-33·9]	32·9 [17·6-61·0]	58·6 [42·5-80·5]	86·6 [83·7-89·3]
Bangladesh	3·5 [1·1-7·3]	10·8 [7·4-14·2]	1·5 [0·1-4·7]	7·9 [2·6-16·2]	244 [184-304]	28·5 [26·3-30·9]	5·5 [1·8-14·7]	45·3 [28·9-71·0]	64·0 [49·6-82·6]	70·8 [62·4-78·5]
Brazil	5·2 [3·3-7·6]	10·7 [7·9-13·5]	1·6 [0·1-4·8]	13·7 [4·2-28·4]	610 [461-760]	13·3 [9·3-17·8]	21·9 [12·7-36·9]	35·6 [23·1-54·4]	54·4 [48·7-60·9]	49·1 [48·0-50·3]
Congo DRC	8·8 [4·7-14·1]	5·0 [3·5-6·4]	0·7 [0·1-1·9]	2·0 [0·6-4·4]	151 [114-188]	28·3 [25·5-31·3]	32·1 [18·5-55·0]	58·7 [48·4-71·1]	66·4 [54·5-81·0]	62·5 [61·7-63·4]
Ethiopia	9·6 [5·1-15·4]	8·0 [5·7-10·4]	1·0 [0·1-3·0]	4·2 [1·3-8·7]	527 [398-655]	23·9 [21·7-26·0]	35·5 [21·3-57·9]	61·5 [49·3-74·2]	62·5 [48·5-77·8]	76·9 [49·3-90·1]
Ghana	12·7 [6·7-20·4]	20·1 [14·3-26·1]	1·0 [0·1-3·1]	5·3 [1·7-10·9]	892 [674-1111]	27·2 [22·8-31·8]	5·3 [2·0-13·5]	10·8 [4·3-25·7]	29·3 [15·0-56·4]	69·3 [67·5-71·0]
Haiti	4·1 [2·6-6·0]	1·7 [1·3-2·1]	1·2 [0·1-3·6]	5·4 [1·7-11·2]	657 [495-819]	25·0 [20·4-30·0]	29·2 [16·8-50·3]	47·4 [35·1-63·8]	52·0 [35·2-77·2]	74·6 [72·6-76·5]
Indonesia	4·4 [1·4-9·1]	20·8 [14·3-27·3]	2·5 [0·2-7·7]	17·2 [5·3-36·0]	245 [185-305]	47·8 [46·7-49·0]	24·7 [17·7-34·3]	27·4 [19·9-37·6]	40·2 [35·2-45·9]	69·9 [69·0-70·7]
India	3·5 [1·1-7·2]	10·2 [7·0-13·4]	2·2 [0·2-6·5]	14·3 [4·6-29·7]	290 [219-362]	27·3 [25·9-28·8]	13·9 [5·8-32·0]	37·4 [22·5-61·9]	57·0 [44·5-73·3]	71·2 [70·8-71·7]
Kenya	11·6 [6·2-18·6]	15·9 [11·3-20·6]	1·9 [0·2-5·5]	9·4 [2·9-19·7]	744 [562-925]	12·5 [11·2-13·9]	27·5 [14·7-50·9]	28·3 [14·6-53·7]	47·7 [32·2-70·8]	80·3 [79·8-80·9]
Cambodia	2·8 [1·1-5·3]	6·8 [4·5-9·1]	1·7 [0·1-5·4]	9·4 [2·9-19·8]	1088 [823-1354]	56·8 [54·5-59·1]	40·2 [26·1-60·3]	59·9 [47·7-72·8]	53·3 [37·8-73·7]	85·8 [83·2-88·2]
Liberia	8·7 [4·6-14·0]	4·8 [3·4-6·2]	0·4 [0·0-1·4]	1·6 [0·5-3·4]	341 [258-425]	23·4 [20·4-26·6]	56·8 [47·3-67·8]	63·6 [53·4-75·3]	68·5 [58·2-80·4]	76·9 [49·3-90·1]
Lesotho	10·0 [5·3-16·2]	9·9 [7·0-12·9]	1·2 [0·1-3·5]	6·4 [2·0-13·4]	1132 [855-1411]	34·8 [32·9-36·8]	32·9 [18·8-56·0]	33·1 [18·1-59·5]	40·5 [23·8-67·6]	65·4 [62·4-68·3]
Mongolia	3·8 [1·5-7·3]	16·7 [11·2-22·3]	2·4 [0·2-7·4]	16·1 [4·8-34·1]	3056 [2307-3803]	39·8 [34·4-45·4]	12·3 [5·0-28·8]	17·5 [7·7-38·6]	28·7 [15·6-52·3]	75·7 [74·4-76·9]
Mozambique	8·6 [4·6-13·8]	4·2 [2·9-5·4]	0·8 [0·1-2·4]	3·5 [1·1-7·3]	267 [201-333]	35·2 [31·9-38·6]	10·6 [4·1-25·8]	28·2 [14·1-55·5]	27·7 [13·9-54·3]	75·0 [29·1-99·2]
Malawi	8·8 [4·7-14·2]	5·2 [3·7-6·8]	0·8 [0·1-2·3]	3·0 [1·0-6·2]	667 [505-831]	19·5 [16·9-22·1]	38·0 [23·9-58·8]	57·9 [45·3-71·9]	72·7 [58·7-81·3]	88·3 [88·0-88·7]
Namibia	18·0 [9·5-28·9]	40·8 [28·9-52·8]	4·6 [0·5-13·5]	39·3 [10·4-87·2]	2470 [1863-3081]	37·9 [34·9-40·9]	22·9 [11·1-45·9]	34·5 [19·4-60·9]	54·0 [39·3-74·4]	85·6 [84·8-86·4]
Pakistan	3·2 [1·0-6·6]	6·9 [4·7-9·0]	1·3 [0·1-3·8]	7·7 [2·4-16·2]	130 [98-162]	28·0 [25·9-30·1]	36·8 [22·6-59·3]	38·4 [22·7-64·2]	49·9 [34·6-71·8]	81·8 [80·2-83·3]
Rwanda	9·3 [4·9-15·0]	7·0 [5·0-9·1]	1·0 [0·1-2·8]	4·3 [1·4-9·0]	1547 [1165-1928]	6·3 [5·1-7·6]	35·1 [21·0-57·8]	44·9 [29·1-68·1]	43·0 [25·7-70·8]	81·1 [78·0-83·9]
Somalia	8·2 [4·4-13·2]	2·8 [2·0-3·6]	1·5 [0·1-4·5]	6·9 [2·2-14·4]	511 [386-636]	21·8 [19·0-24·7]	46·4 [33·0-64·9]	30·9 [16·0-58·6]	65·8 [56·6-76·5]	76·4 [73·9-78·8]
Eswatini	15·8 [8·4-25·4]	32·3 [22·9-41·9]	3·5 [0·3-10·3]	25·6 [8·1-53·1]	3800 [2869-4735]	30·3 [27·7-33·0]	26·4 [13·5-50·0]	38·7 [23·0-64·3]	38·7 [22·2-66·1]	78·2 [71·0-84·7]
Thailand	5·9 [1·9-12·1]	37·9 [26·1-49·7]	5·7 [0·6-16·6]	49·5 [15·9-101·2]	357 [270-444]	33·7 [29·2-38·4]	26·4 [13·7-49·7]	29·8 [15·5-56·0]	52·7 [37·9-73·3]	84·9 [83·2-86·6]
Tajikistan	5·0 [3·4-6·8]	2·7 [1·7-3·7]	1·0 [0·1-3·0]	5·1 [1·6-10·6]	840 [633-1046]	19·0 [15·5-22·8]	13·3 [5·3-31·3]	20·3 [9·0-44·1]	70·8 [66·2-75·7]	75·0 [73·9-76·0]
Timor-Leste	3·5 [1·1-7·1]	9·8 [6·8-12·9]	1·7 [0·2-4·7]	8·5 [2·7-17·6]	568 [429-706]	47·8 [46·7-49·0]	24·0 [11·1-48·3]	49·8 [34·5-71·1]	47·2 [32·6-68·1]	71·3 [63·5-78·5]
Tanzania	10·0 [5·3-16·1]	9·8 [6·9-12·6]	1·2 [0·1-3·5]	5·6 [1·9-11·3]	488 [368-607]	29·6 [28·1-31·2]	27·6 [14·4-51·2]	35·1 [19·7-61·2]	43·9 [27·3-69·4]	87·9 [75·1-96·5]
Uganda	9·3 [5·0-15·0]	7·2 [5·1- 9·4]	1·0 [0·1-2·9]	4·4 [1·3-9·2]	371 [281-462]	14·8 [11·7-18·1]	26·8 [14·0-49·3]	38·0 [24·2-58·6]	57·3 [45·1-72·8]	61·1 [51·6-70·3]
South Africa	20·0 [10·6-32·2]	48·9 [34·6-63·2]	6·9 [0·6-21·2]	61·7 [18·2-130·9]	887 [671-1102]	31·5 [28·0-35·2]	50·1 [37·4-66·8]	52·5 [37·5-73·6]	60·5 [45·6-80·4]	89·7 [89·3-90·2]
Zambia	10·3 [5·4-16·5]	10·7 [7·6-13·9]	0·9 [0·1-2·9]	4·5 [1·4-9·4]	565 [427-705]	31·6 [28·2-35·2]	41·4 [29·1-58·4]	48·9 [36·0-66·2]	42·2 [27·1-65·7]	80·7 [80·1-81·4]
Zimbabwe	10·0 [5·3-16·1]	9·8 [7·0-12·7]	3·9 [0·3-11·6]	34·3 [12·1-68·0]	525 [397-653]	20·8 [17·7-24·2]	26·5 [14·2-48·6]	38·7 [24·5-61·2]	57·6 [46·7-71·0]	79·1 [74·9-83·1]

Values in the table indicate means and 95% uncertainty intervals. Details regarding parameter sources and estimation are provided in the main text (Table 1) and in the pages 3-8 of this supplement. Costs are all presented in 2020 USD. "CXR" = chest Xray, "LTBI" = latent tuberculosis infection. We sampled from the distributions shown in Table 1 by fitting gamma distributions to the reported means and standard deviations on chest Xray, inpatient bed day, and outpatient visit costs, normal distributions to the reported means and standard deviations on contact investigation and TB treatment unit costs, and beta distributions to the reported means and 95% confidence intervals on latent TB prevalence. More details regarding the estimation of background notification and treatment success probabilities are shown on pages 6-7.

Appendix Table 3: Parameter distributions

Parameter	Distribution (PLWHA)	Distribution (Household Contacts)	References
**TB Preventive Treatment**			
3HP Efficacy	Beta (34·8, 5·2)	Beta (34·8, 5·2)	15,54
3HP Completion	Beta (77·3, 13·6)	Beta (77·3, 13·6)	18,21,55,56
1HP Completion	3HP completion + Uniform (0, 0·15)*	3HP completion + Uniform (0, 0·15)*	1HP completion was assumed to be higher on average than 3HP completion
Adverse reaction (not requiring hospitalization) for 3HP, 1HP	Beta (106, 1962)	-Contacts < 15: Beta (7, 464)<br>-Contacts 15+: Beta (106, 1962)	21–23
Adverse reaction (requiring hospitalization) for 3HP, 1HP	Beta (11, 194)	-Contacts < 15: Beta (1, 470)<br>-Contacts 15+: Beta (9, 1852)	21–23
**TB Natural History & Mortality**			
LTBI prevalence	see Appendix Table 2	Beta (99·4, 180·6)	5,31
Annual TB progression (among those with LTBI; see next page for details)	-New, year 1: Beta (9·8, 165·2) times Normal (1·32, 0·08)<br>-New, year 2: Beta (9·8, 165·2) times Normal (0·59, 0·03)<br>-Established, year 1: Beta (9·8, 165·2) times Normal (1·32, 0·08) times Beta (22·4, 201·6) divided by Beta (23·7, 101·0)<br>-Established, year 2: Beta (9·8, 165·2) times Normal (0·59, 0·03) divided by Beta (23·7, 101·0)<br>-On ART: years 3-9: Beta (9·8, 165·2) times Normal (0·12, 0·04)<br>-On ART: years 10+: Beta (9·8, 165·2) times Normal (0·12, 0·04) times Normal (0·5, 0·13)<br>-Not on ART, year 2: Beta (9·8, 165·2) times Normal (0·59, 0·03) times Normal (2·5, 0·25)<br>-Not on ART, years 3-9: Beta (9·8, 165·2) times Normal (0·12, 0·04) times Normal (2·5, 0·25)<br>-Not on ART, years 10+: Beta (9·8, 165·2) times Normal (0·12, 0·04) times Normal (0·5, 0·13) times Normal (2·5, 0·25)	-Contacts < 5, first 2 years: Beta (6, 54)<br>-Contacts 5+, first 2 years: Beta (5·1, 94·9)<br>-All contacts, years 2-4: Beta (12, 5988)<br>-All contacts, years 5-9: Beta (6, 5988)<br>-All contacts, years 10+: Beta (80, 99920)	15–17,26,27,32
TB case-fatality ratio	-Not on ART: Beta (20·8, 62·5)<br>-On ART: mortality not on ART, multiplied by Beta (21, 29)	-Contacts < 5, on treatment: Gamma (2·16, 0·012)<br>-Contacts < 5, not on treatment: Beta (87·2, 112·8)<br>-Contacts 5+, on treatment: Beta (4·5, 495·5)<br>-Contacts 5+, not on treatment: Beta (49·7, 283·7)	28,33,34
Background notification rate	-On ART: 100% (no uncertainty)<br>-Not on ART: see Appendix Table 2	see Appendix Table 2	14
Probability of treatment success	see Appendix Table 2	see Appendix Table 2	14
**TB Contact Investigation Parameters†**			
Active TB prevalence	NA	-Contacts < 5: Beta (6·7, 60)<br>-Contacts 5-14: Gamma (3·61, 0·026)<br>-Contacts 15+: Beta (12·8, 387·2)	5
Prevalence of positive screen	NA	-Contacts < 5 (symptom only): Beta (241·5, 458·5)<br>-Contacts 5-14 (symptom or CXR): Normal (0·28, 0·1)<br>-Contacts 15+ (symptom or CXR): Gamma (20·25, 0·0089)	10–13
CXR Sensitivity	NA	-Contacts < 5: Beta (43, 7)	7
CXR Specificity	NA	-Contacts < 5: Beta (6·3, 0·3)	7
Xpert Sensitivity	NA	-Contacts 5-14: Beta (87·6, 32·4)<br>-Contacts 15+: Beta (136·5, 13·5)	8,9
Xpert Specificity	NA	-Contacts 5+: Beta (293·1, 6·9)	8,9

TPT initiation (i.e., 1 minus between household visit and facility visit for TPT)	NA	Beta (240, 85)	6
**Costs (see Appendix Table 2 for country-varying cost parameters)**			
Xpert	NA†	Gamma (61·36, 0·383)	44
Labs for adverse reaction	Normal (18·2, 2·5)	Normal (18·2, 2·5)	24
**Disability Weights & Non-TB Mortality**			
All-cause mortality	-New on ART: Normal (0·08, 0·02)**<br>-Established: Normal (0·03, 0·01)***<br>-Not on ART:, Normal (0·03, 0·01) times Normal (3, 0·5)<br>We multiplied each mortality rate by 1 minus Beta (14, 36)††	NA – varies by country	35–38
TB disability weight	Beta (20·4, 29·6)	Beta (22·2, 44·4)	57
Non-TB disability weight	-On ART: Beta (23·4, 276·6)<br>-Not on ART: Beta (24·0, 63·6)	NA – assumed to equal 0	57
**Target Population Size Projections (only those varied in the PSA are shown)**			
Transition off ART (i.e., from loss to follow-up)	-New on ART: Beta (75, 425)<br>-Established: Normal (0·08, 0·02)***	NA	35–38
Transition back on ART if LTFU	Beta (72, 28)	NA	Calibrated (see appendix page 6 for details)
Life expectancy	Normal (27, 1)	NA – varies by country	30

Ages of household contacts are displayed in years. For beta-distributed quantities, the alpha and beta parameters are shown (such that the mean of the distribution equals alpha divided by alpha plus beta). For gamma-distributed quantities, the shape and scale parameters are shown. For normally-distributed quantities, the mean and standard deviation are shown. For uniformly-distributed parameters, the minimum and maximum are shown. Distributions were selected and parameterized to best fit the means and uncertainty reported in Table 1 in the main text. *Truncated above at 100%; ** Truncated below at 1%; ***Truncated below at 0·5%; † Relevant for household contacts only; †† The estimated percent of HIV deaths in Africa that are related to TB from IHME's global burden of disease[58] CXR = chest Xray, LTFU = lost-to-follow-up.

Annual TB Reactivation

The probability of progression from latent infection to active disease was assumed to depend on time since entering the model, age (for household contacts only), and ART status (for PLWHA only). Estimates are shown in Appendix Table 4.

Appendix Table 4: Annual progression probabilities

Year	Contacts < 5 years	Contacts 5-14 years	Contacts ≥ 15 years	PLWHA newly enrolled on ART	PLWHA established on ART	PLWHA not on ART
1	10% [4-21%]	5% [2-11%]	5% [2-11%]	7·4% [3·2-12·6%]	4·0% [1·5-8·2%]	NA*
2	10% [4-21%]	5% [2-11%]	5% [2-11%]	3·3% [1·6-5·6%]	1·8% [0·7-3·7%]	8·3% [3·8-14·2%]
3	0·2% [0·1-0·3%]	0·2% [0·1-0·3%]	0·2% [0·1-0·3%]	0·7% [0·2-1·4%]	0·7% [0·2-1·4%]	1·7% [0·4-3·5%]
4	0·2% [0·1-0·3%]	0·2% [0·1-0·3%]	0·2% [0·1-0·3%]	0·7% [0·2-1·4%]	0·7% [0·2-1·4%]	1·7% [0·4-3·5%]
5	0·2% [0·1-0·3%]	0·2% [0·1-0·3%]	0·2% [0·1-0·3%]	0·7% [0·2-1·4%]	0·7% [0·2-1·4%]	1·7% [0·4-3·5%]
6	0·1% [0·05-0·2%]	0·1% [0·05-0·2%]	0·1% [0·05-0·2%]	0·7% [0·2-1·4%]	0·7% [0·2-1·4%]	1·7% [0·4-3·5%]
7	0·1% [0·05-0·2%]	0·1% [0·05-0·2%]	0·1% [0·05-0·2%]	0·7% [0·2-1·4%]	0·7% [0·2-1·4%]	1·7% [0·4-3·5%]
8	0·1% [0·05-0·2%]	0·1% [0·05-0·2%]	0·1% [0·05-0·2%]	0·7% [0·2-1·4%]	0·7% [0·2-1·4%]	1·7% [0·4-3·5%]
9	0·1% [0·05-0·2%]	0·1% [0·05-0·2%]	0·1% [0·05-0·2%]	0·7% [0·2-1·4%]	0·7% [0·2-1·4%]	1·7% [0·4-3·5%]
10	0·08% [0·06-0·09%]	0·08% [0·06-0·09%]	0·08% [0·06-0·09%]	0·33% [0·08-0·78%]	0·33% [0·08-0·78%]	0·88% [0·19-1·9%]

For household contacts, progression is based on current age, not age upon which a contact entered the model. Year indicates the year since entering the model.

*All modeled cohorts start out on ART

We estimated annual progression probabilities of 10% [4-21%] for LTBI household contacts < 5 years and 5% [2-11%] for LTBI household contacts ≥ 5 years in the first two years since entering the model (i.e., the first two years after someone in their households was diagnosed with TB). These estimates were based on age-stratified pooled estimates of cumulative TB incidence among household contacts with positive TST/IGRA over 2 years from a recent meta-analysis of contact cohort studies by Martinez et al. (see their Figure 3A).[15] We estimated the relative decline in progression risk after two years based on estimates from a modeling study of TB progression over time by Menzies et al.[26] (see their Figure 1A) and a systematic review of cohort studies that measured TB reactivation > 2 years post-infection by Dale et al.[27] (see their Figure 2). Based on these two studies, the assumption that most household contacts with LTBI have been recently infected, and the assumption that after the first two years contacts originally < 5 years old would no longer be at higher risk of disease progression than older contacts, we estimated that annual progression risk among all household contacts would drop to 0·2% [0·1-0·3%] after 2 years, 0·1% [0·05-0·2%] after 5 years, and 0·08% [0·06-0·09%] after 10 years.

We estimated that PLWHA newly enrolled on ART would have a 7·4% [3·2-12·6%] chance of progressing to active disease in the first year since initiating ART and a 3·3% [1·6-5·6%] chance of progressing in the second year, based on progression to active disease among those with positive TST that were randomized not to receive IPT in a trial of the impact of ART and IPT on TB incidence by Golub et al.[16] In particular, we started with the 2-year probability of infection among non-IPT initiators with a positive TST (11·5%). We subtracted from this the probability of infection among IPT initiators with a positive TST (1·6%; to proxy incidence attributable to reinfections, rather than progression) and adjusted for the proportion of non-IPT initiators that had not yet initiated ART (26%) by applying the study-estimated relative hazard of developing TB without ART (vs. with ART; 0·41 [0·31-0·54]). This yielded a two-year probability of infection of 7·2% among ART initiators. For PLWHA who are newly enrolled on ART, who

are assumed to be at a higher risk of progression than established PLWHA, we adjusted this two-year probability of infection upward by accounting for the percentage of patients in the trial that had received ART earlier on (76%) and the relative hazard of developing TB disease with higher vs. medium CD4 counts (0·54 [0·31-0·90]). This yielded an annual probability of progression of 5·6% among newly enrolled PLWHA. We then applied data from a Swiss cohort study authored by Elz et al.[32] to account for the higher incidence rate in year 1 (1·32 [1·19-1·45] relative risk vs. years 1 and 2 combined) compared to year 2 (0·59 [0·54-0·64] relative risk vs. years 1 and 2 combined). We propagated uncertainty in all estimates/trial-reported data to estimate that newly enrolled PLWHA with LTBI face an annual progression risk of 7·4% [3·2-12·6%] in the first year of observation and 3·3% [1·6-5·6%] in the second year of observation. We assumed that in years 1 and 2, established PLWHA face approximately half the risks of newly enrolled PLWHA (0·54 [0·31-0·90]), again based on the relative hazard by CD4 count estimates reported in Golub et al.

Based on outcomes among control groups from Golub et al. and the TEMPRANO trial of TPT among PLWHA[16,17], we estimated that PLWHA not on ART continually (i.e., regardless of year of observation) face progression risks that are 2·5 [2-3] times that of PLWHA on ART.

After two years of observation, we assume that progression risks decline over time based on data from the Elz et al. Swiss cohort study (progression in years 2-10 was 0·12 [0·04-0·2] times that of progression in years 0-2).[32] After ten years of observation, we assumed that progression among PLWHA on ART relative to years 2-10 would be similar to the relative progression risk estimated for household contacts, above, based on Menzies et al. and Dale et al.[26,27] We intentionally used data from lower-burden settings to parameterize progression after the second year of observation to reduce the influence that reinfections would have on our estimates.

The risk of progression is assumed to decline over time not because “year of observation” is equivalent to “years since infection” but because in high-incidence settings with relatively high annual risks of infection, it is reasonable to assume that the average person with LTBI has been infected recently[59], and that this would be reflected in published data (from trials and cohort studies).

Willingness-to-Pay Threshold Details

Ochalek et al. provide estimates of country-specific cost per DALY thresholds in 2015 US dollars and as a percentage of 2015 GDP per capita, based on four different calculation methods.[60] We used the median percentage of GDP per capita across the four methods and applied the percentages to 2020 GDP per capita to calculate the updated cost-effectiveness thresholds in 2020 US dollars used in the main analysis. Given the evolving evidence on country-specific health opportunity costs, we also compared incremental cost-effectiveness ratios (ICERs) to the full range of thresholds (based on the percentage of GDP per capita ranges for each country from Ochalek et al.), gross national income per capita (less conservative), and $1000 (less conservative for lower-income countries, more conservative for higher-income countries).

Appendix Table 5: CHEERS checklist for economic evaluation studies

Topic	No.	Item	Location where item is reported
**Title**	1	Identify the study as an economic evaluation and specify the interventions being compared.	Title page (page 1)
**Abstract**	2	Provide a structured summary that highlights context, key methods, results, and alternative analyses.	Abstract (page 2)
**Introduction**			
**Background and objectives**	3	Give the context for the study, the study question, and its practical relevance for decision making in policy or practice.	Introduction
**Methods**			
**Health economic analysis plan**	4	Indicate whether a health economic analysis plan was developed and where available.	Not Applicable
**Study population**	5	Describe characteristics of the study population (such as age range, demographics, socioeconomic, or clinical characteristics).	Methods (paragraphs 1-4)
**Setting and location**	6	Provide relevant contextual information that may influence findings.	Methods (paragraphs 1-4)
**Comparators**	7	Describe the interventions or strategies being compared and why chosen.	Methods (paragraphs 5-6)
**Perspective**	8	State the perspective(s) adopted by the study and why chosen.	Methods (paragraph 7)
**Time horizon**	9	State the time horizon for the study and why appropriate.	Methods (paragraph 4)
**Discount rate**	10	Report the discount rate(s) and reason chosen.	Methods (paragraph 7)
**Selection of outcomes**	11	Describe what outcomes were used as the measure(s) of benefit(s) and harm(s).	Methods (paragraph 7)
**Measurement of outcomes**	12	Describe how outcomes used to capture benefit(s) and harm(s) were measured.	Methods (paragraphs 1-7, Table 1)
**Valuation of outcomes**	13	Describe the population and methods used to measure and value outcomes.	Methods (paragraphs 1-7, Table 1)
**Measurement and valuation of resources and costs**	14	Describe how costs were valued.	Methods (paragraphs 1-4, Table 1)
**Currency, price date, and conversion**	15	Report the dates of the estimated resource quantities and unit costs, plus the currency and year of conversion.	Methods (paragraph 7)
**Rationale and description of model**	16	If modelling is used, describe in detail and why used. Report if the model is publicly available and where it can be accessed.	Methods (paragraphs 1-4), Figure 1
**Analytics and assumptions**	17	Describe any methods for analysing or statistically transforming data, any extrapolation methods, and approaches for validating any model used.	Methods (paragraphs 3-4), Table 1, Appendix pages 3-13
**Characterizing heterogeneity**	18	Describe any methods used for estimating how the results of the study vary for subgroups.	Not Applicable
**Characterizing distributional effects**	19	Describe how impacts are distributed across different individuals or adjustments made to reflect priority populations.	Not Applicable
**Characterizing uncertainty**	20	Describe methods to characterise any sources of uncertainty in the analysis.	Methods (paragraph 7)
**Approach to engagement with patients and others affected by the study**	21	Describe any approaches to engage patients or service recipients, the general public, communities, or stakeholders (such as clinicians or payers) in the design of the study.	Not Applicable
**Results**			
**Study parameters**	22	Report all analytic inputs (such as values, ranges, references) including uncertainty or distributional assumptions.	Table 1, Appendix pages 4-14
**Summary of main results**	23	Report the mean values for the main categories of costs and outcomes of interest and summarise them in the most appropriate overall measure.	Results (paragraphs 1-7), Figure 2, Figure 3
**Effect of uncertainty**	24	Describe how uncertainty about analytic judgments, inputs, or projections affect findings. Report the effect of choice of discount rate and time horizon, if applicable.	Results (paragraphs 8-9), Appendix pages 41-76
**Effect of engagement with patients and others affected by the study**	25	Report on any difference patient/service recipient, general public, community, or stakeholder involvement made to the approach or findings of the study	Not Applicable
**Discussion**			
**Study findings, limitations, generalizability, and current knowledge**	26	Report key findings, limitations, ethical or equity considerations not captured, and how these could affect patients, policy, or practice.	Discussion
**Other relevant information**			

Topic	No.	Item	Location where item is reported
**Source of funding**	27	Describe how the study was funded and any role of the funder in the identification, design, conduct, and reporting of the analysis	Abstract, Methods (last paragraph), Funding Statement
**Conflicts of interest**	28	Report authors conflicts of interest according to journal or International Committee of Medical Journal Editors requirements.	Declaration of Interests

Supplementary Methods References

1 World Health Organization. Global Tuberculosis Report 2022. 2022 https://www.who.int/publications-detail-redirect/9789240061729 (accessed Dec 21, 2022).

2 UNAIDS. AIDSinfo: Global data on HIV epidemiology and response. https://aidsinfo.unaids.org/ (accessed March 23, 2023).

3 WHO. TB Data. 2021; published online Oct 14. https://www.who.int/teams/global-tuberculosis-programme/data (accessed Aug 15, 2022).

4 United Nations Population Division. Household Size and Composition Data. 2019. https://www.un.org/development/desa/pd/data/household-size-and-composition (accessed Aug 25, 2022).

5 Fox GJ, Barry SE, Britton WJ, Marks GB. Contact investigation for tuberculosis: a systematic review and meta-analysis. *European Respiratory Journal* 2013; **41**: 140–56.

6 Mandalakas AM, Hesseling AC, Kay A, *et al.* Tuberculosis prevention in children: a prospective community-based study in South Africa. *Eur Respir J* 2021; **57**: 2003028.

7 Vonasek B, Ness T, Takwoingi Y, *et al.* Screening tests for active pulmonary tuberculosis in children. *Cochrane Database of Systematic Reviews* 2021. DOI:10.1002/14651858.CD013693.pub2.

8 Kay AW, González Fernández L, Takwoingi Y, *et al.* Xpert MTB/RIF and Xpert MTB/RIF Ultra assays for active tuberculosis and rifampicin resistance in children. *Cochrane Database Syst Rev* 2020; **2020**: CD013359.

9 Zifodya JS, Kreniske JS, Schiller I, *et al.* Xpert Ultra versus Xpert MTB/RIF for pulmonary tuberculosis and rifampicin resistance in adults with presumptive pulmonary tuberculosis. *Cochrane Database of Systematic Reviews* 2021. DOI:10.1002/14651858.CD009593.pub5.

10 Frascella B, Richards AS, Sossen B, *et al.* Subclinical Tuberculosis Disease—A Review and Analysis of Prevalence Surveys to Inform Definitions, Burden, Associations, and Screening Methodology. *Clin Infect Dis* 2020; **73**: e830–41.

11 Martinez L, Shen Y, Handel A, *et al.* Effectiveness of WHO's pragmatic screening algorithm for child contacts of tuberculosis cases in resource-constrained settings: a prospective cohort study in Uganda. *The Lancet Respiratory Medicine* 2018; **6**: 276–86.

12 Kruk A, Gie RP, Schaaf HS, Marais BJ. Symptom-Based Screening of Child Tuberculosis Contacts: Improved Feasibility in Resource-Limited Settings. *Pediatrics* 2008; **121**: e1646–52.

13 Triasih R, Robertson CF, Duke T, Graham SM. A Prospective Evaluation of the Symptom-Based Screening Approach to the Management of Children Who Are Contacts of Tuberculosis Cases. *Clinical Infectious Diseases* 2015; **60**: 12–8.

14 World Health Organization. Global Tuberculosis Report. 2020. https://www.who.int/teams/global-tuberculosis-programme/tb-reports (accessed Aug 4, 2021).

15 Martinez L, Cords O, Horsburgh CR, *et al.* The risk of tuberculosis in children after close exposure: a systematic review and individual-participant meta-analysis. *The Lancet* 2020; **395**: 973–84.

16 Golub JE, Saraceni V, Cavalcante SC, *et al.* The impact of antiretroviral therapy and isoniazid preventive therapy on tuberculosis incidence in HIV-infected patients in Rio de Janeiro, Brazil. *AIDS* 2007; **21**: 1441–8.

17 The TEMPRANO ANRS 12136 Study Group. A Trial of Early Antiretrovirals and Isoniazid Preventive Therapy in Africa. *New England Journal of Medicine* 2015; **373**: 808–22.

18 Belknap R, Holland D, Feng P-J, *et al.* Self-administered Versus Directly Observed Once-Weekly Isoniazid and Rifapentine Treatment of Latent Tuberculosis Infection: A Randomized Trial. *Ann Intern Med* 2017; **167**: 689–97.

19 Munseri PJ, Talbot EA, Mtei L, Fordham von Reyn C. Completion of isoniazid preventive therapy among HIV-infected patients in Tanzania. *The International Journal of Tuberculosis and Lung Disease* 2008; **12**: 1037–41.

20 Rangaka MX, Wilkinson RJ, Boulle A, *et al.* Isoniazid plus antiretroviral therapy to prevent tuberculosis: a randomised double-blind, placebo-controlled trial. *The Lancet* 2014; **384**: 682–90.

21 Villarino ME, Scott NA, Weis SE, *et al.* Treatment for Preventing Tuberculosis in Children and Adolescents: A Randomized Clinical Trial of a 3-Month, 12-Dose Regimen of a Combination of Rifapentine and Isoniazid. *JAMA Pediatrics* 2015; **169**: 247–55.

22 Sterling TR, Villarino ME, Borisov AS, *et al.* Three Months of Rifapentine and Isoniazid for Latent Tuberculosis Infection. *New England Journal of Medicine* 2011; **365**: 2155–66.

23 Sterling TR, Scott NA, Miro JM, *et al.* Three months of weekly rifapentine and isoniazid for treatment of Mycobacterium tuberculosis infection in HIV-coinfected persons. *AIDS* 2016; **30**: 1607–15.

24 South Africa National Health Laboratory Service. State Price List. 2018. https://cdn.ymaws.com/www.paediatrics.org.za/resource/resmgr/nhls_state_price_list_2018.pdf (accessed Dec 16, 2021).

25 World Health Organization. WHO-CHOICE Estimates of cost for inpatient and outpatient health service delivery. 2011. https://cdn.who.int/media/docs/default-source/health-economics/who-choice-estimates-of-cost-for-inpatient-and-outpatient-health-service-delivery.pdf?sfvrsn=b814d37e_1&download=true.

26 Menzies NA, Swartwood N, Testa C, *et al.* Time Since Infection and Risks of Future Disease for Individuals with Mycobacterium tuberculosis Infection in the United States. *Epidemiology* 2021; **32**: 70–8.

27 Dale KD, Karmakar M, Snow KJ, Menzies D, Trauer JM, Denholm JT. Quantifying the rates of late reactivation tuberculosis: a systematic review. *The Lancet Infectious Diseases* 2021; published online April 20. DOI:10.1016/S1473-3099(20)30728-3.

28 Jenkins HE, Yuen CM, Rodriguez CA, *et al.* Mortality in children diagnosed with tuberculosis: a systematic review and meta-analysis. *The Lancet Infectious Diseases* 2017; **17**: 285–95.

29 AIDSinfo | UNAIDS. http://aidsinfo.unaids.org/ (accessed April 1, 2019).

30 Mills EJ, Bakanda C, Birungi J, *et al.* Life expectancy of persons receiving combination antiretroviral therapy in low-income countries: a cohort analysis from Uganda. *Ann Intern Med* 2011; **155**: 209–16.

31 Houben RMGJ, Dodd PJ. The Global Burden of Latent Tuberculosis Infection: A Re-estimation Using Mathematical Modelling. *PLOS Medicine* 2016; **13**: e1002152.

32 Elz L, Schlegel M, Weber R, *et al.* Reducing Tuberculosis Incidence by Tuberculin Skin Testing, Preventive Treatment, and Antiretroviral Therapy in an Area of Low Tuberculosis Transmission. *Clinical Infectious Diseases* 2007; **44**: 94–102.

33 Odone A, Amadasi S, White RG, Cohen T, Grant AD, Houben RMGJ. The Impact of Antiretroviral Therapy on Mortality in HIV Positive People during Tuberculosis Treatment: A Systematic Review and Meta-Analysis. *PLOS ONE* 2014; **9**: e112017.

34 Mukadi YD, Maher D, Harries A. Tuberculosis case fatality rates in high HIV prevalence populations in sub-Saharan Africa. *AIDS* 2001; **15**: 143–52.

35 Bernard C, Balestre E, Coffie PA, *et al.* Aging with HIV: what effect on mortality and loss to follow-up in the course of antiretroviral therapy? The IeDEA West Africa Cohort Collaboration. *HIV AIDS (Auckl)* 2018; **10**: 239–52.

36 Cornell M, Johnson LF, Schomaker M, *et al.* Age in antiretroviral therapy programmes in South Africa: a multi-centre observational cohort study. *Lancet HIV* 2015; **2**: e368–75.

37 Tiendrebeogo T, Messou E, Arikawa S, *et al.* Ten-year attrition and antiretroviral therapy response among HIV-positive adults: a sex-based cohort analysis from eight West African countries. *Journal of the International AIDS Society* 2021; **24**: e25723.

38 Mugglin C, Haas AD, van Oosterhout JJ, *et al.* Long-term retention on antiretroviral therapy among infants, children, adolescents and adults in Malawi: A cohort study. *PLoS One* 2019; **14**: e0224837.

39 Jenny Lei Ravelo. Pricing agreement brings HIV drug darunavir within reach of LMICs. Devex. 2021; published online July 27. https://www.devex.com/news/sponsored/pricing-agreement-brings-hiv-drug-darunavir-within-reach-of-lmics-100478 (accessed Aug 11, 2021).

40 Marais BJ, Schaaf HS. Tuberculosis in Children. *Cold Spring Harb Perspect Med* 2014; **4**: a017855.

41 Chikovani I, Shengelia N, Marjanishvili N, *et al.* Cost of TB services in the public and private sectors in Georgia. *Int J Tuberc Lung Dis* 2021; **25**: 1019–27.

42 Pallas SW, Courey M, Hy C, Killam WmP, Warren D, Moore B. Cost analysis of tuberculosis diagnosis in Cambodia with and without Xpert® MTB/RIF for people living with HIV/AIDS and people with presumptive multidrug-resistant tuberculosis. *Appl Health Econ Health Policy* 2018; **16**: 537–48.

43 Pande T, Pai M, Khan FA, Denkinger CM. Use of chest radiography in the 22 highest tuberculosis burden countries. *European Respiratory Journal* 2015; **46**: 1816–9.

44 Pooran A, Theron G, Zijenah L, *et al.* Point of care Xpert MTB/RIF versus smear microscopy for tuberculosis diagnosis in southern African primary care clinics: a multicentre economic evaluation. *Lancet Glob Health* 2019; **7**: e798–807.

45 Chatterjee S, Pant M, Haldar P, Aggarwal MK, Laxminarayan R. Current costs & projected financial needs of India’s Universal Immunization Programme. *Indian Journal of Medical Research* 2016; **143**: 801.

46 Kakinda M, Matovu JKB. A yield and cost comparison of tuberculosis contact investigation and intensified case finding in Uganda. *PLOS ONE* 2020; **15**: e0234418.

47 Shah L, Peña MR, Mori O, *et al.* A pragmatic stepped-wedge cluster randomized trial to evaluate the effectiveness and cost-effectiveness of active case finding for household contacts within a routine tuberculosis program, San Juan de Lurigancho, Lima, Peru. *International Journal of Infectious Diseases* 2020; **100**: 95–103.

48 Hussain H, Malik A, Ahmed JF, *et al.* Cost-effectiveness of household contact investigation for detection of tuberculosis in Pakistan. *BMJ Open* 2021; **11**: e049658.

49 Yadav RP, Nishikiori N, Satha P, Eang MT, Lubell Y. Cost-Effectiveness of a Tuberculosis Active Case Finding Program Targeting Household and Neighborhood Contacts in Cambodia. *The American Journal of Tropical Medicine and Hygiene* 2014; **90**: 866–72.

50 International Monetary Fund. International Finance Statistics. 2022; published online Aug 23. https://data.imf.org/?sk=4C514D48-B6BA-49ED-8AB9-52B0C1A0179B (accessed Aug 24, 2022).

51 DataBank | The World Bank. https://databank.worldbank.org/home.aspx (accessed Feb 9, 2020).

52 Yuen CM, Majidulla A, Jaswal M, *et al.* Cost of Delivering 12-Dose Isoniazid and Rifapentine Versus 6 Months of Isoniazid for Tuberculosis Infection in a High-Burden Setting. *Clin Infect Dis* 2020; **73**: e1135–41.

53 Arinaminpathy N, Cordier-Lassalle T, Lunte K, Dye C. The Global Drug Facility as an intervention in the market for tuberculosis drugs. *Bull World Health Organ* 2015; **93**: 237-248A.

54 Golub JE, Cohn S, Saraceni V, *et al.* Long-term Protection From Isoniazid Preventive Therapy for Tuberculosis in HIV-Infected Patients in a Medium-Burden Tuberculosis Setting: The TB/HIV in Rio (THRio) Study. *Clinical Infectious Diseases* 2015; **60**: 639–45.

55 Yanes-Lane M, Ortiz-Brizuela E, Campbell JR, *et al.* Tuberculosis preventive therapy for people living with HIV: A systematic review and network meta-analysis. *PLoS Med* 2021; **18**: e1003738.

56 Pease C, Hutton B, Yazdi F, *et al.* Efficacy and completion rates of rifapentine and isoniazid (3HP) compared to other treatment regimens for latent tuberculosis infection: a systematic review with network meta-analyses. *BMC Infect Dis* 2017; **17**: 265.

57 Global Burden of Disease Collaborative Network. Global Burden of Disease Study 2019 (GBD 2019) Disability Weights. 2020. DOI:10.6069/1W19-VX76.

58 Institute for Health Metrics and Evaluation (IHME). GBD Compare Data Visualization. Seattle, WA: IHME, University of Washington, 2020 http://www.healthdata.org/data-visualization/gbd-compare (accessed Nov 15, 2021).

59 Dowdy DW, Behr MA. Are we underestimating the annual risk of infection with Mycobacterium tuberculosis in high-burden settings? *The Lancet Infectious Diseases* 2022; **0**. DOI:10.1016/S1473-3099(22)00153-0.

60 Ochalek J, Lomas J, Claxton K. Estimating health opportunity costs in low-income and middle-income countries: a novel approach and evidence from cross-country data. *BMJ Global Health* 2018; **3**: e000964.

SUPPLEMENTARY RESULTS

Appendix Figure 1: Projected TPT initiation among household contacts and PLWHA in 29 countries

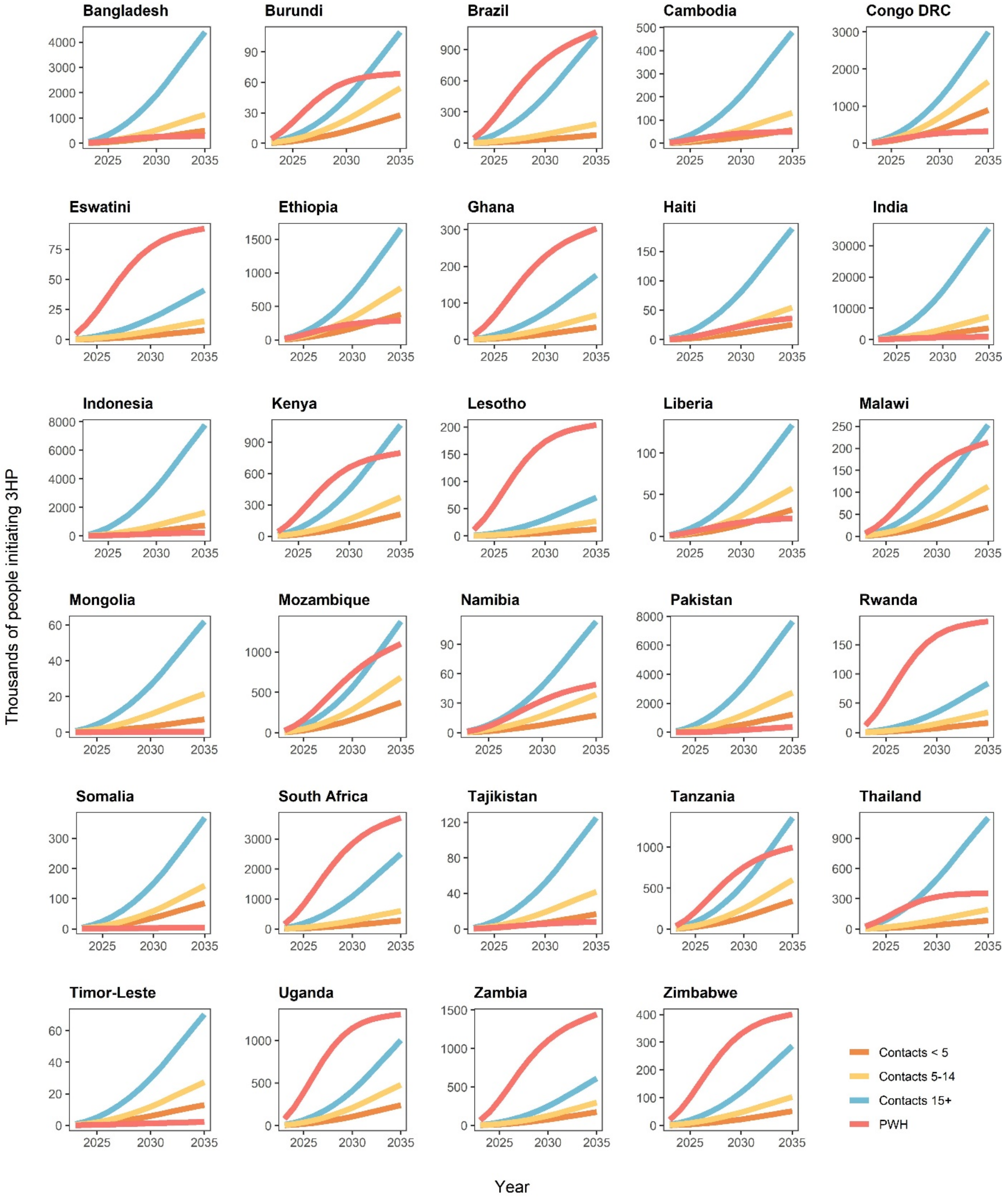

Figure shows the modeled number of people in each country initiating 3HP in each year under the 3HP scenario, by target population. Contacts ages are displayed in years

Appendix Figure 2: Cumulative ten-year costs and health impact of scaling up household contact investigations with or without TPT in 29 countries

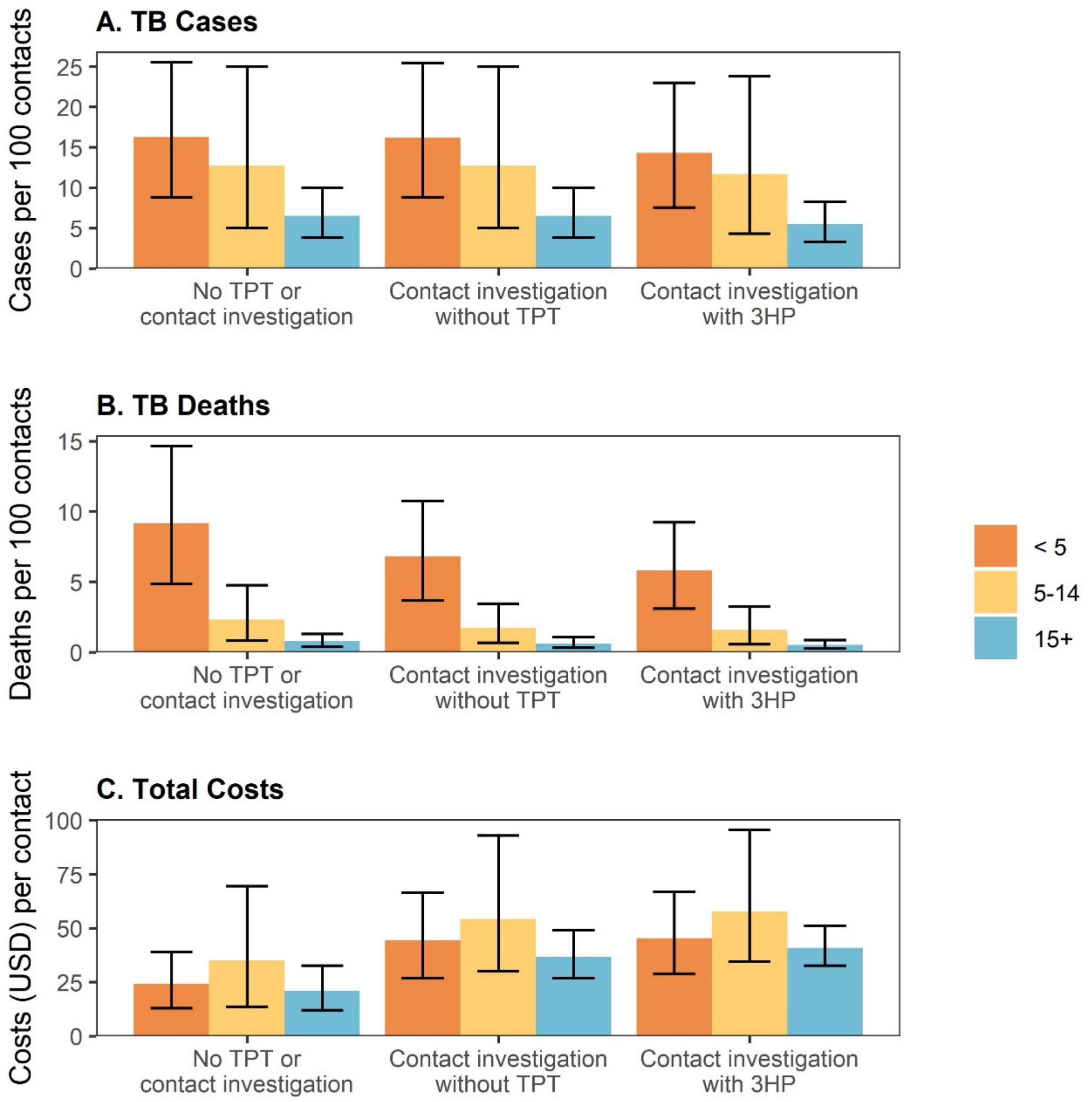

Figure shows how summary outcomes (cumulative TB cases in panel A, cumulative TB Deaths in panel B, and cumulative costs in panel C) vary when contact investigation to screen for active cases only is scaled up ("Contact investigation without TPT") compared to the 3HP ("Contact investigation with 3HP") and no TPT ("No TPT or contact investigation") scenarios presented in the main analysis. Shading indicates contact age group (displayed in years).

Appendix Table 6: Costs of scaling up a 3HP program for household contacts under five years

Country	Scenario	3HP (% of total)	Contact Investigation (% of total)	Toxicity (% of total)	TB Treatment (% of total)	Total Cost (USD millions)	Cost per Contact (USD)
Bangladesh	No TPT	0%	0%	0%	100%	9 [4-18]	6.4 [2.7-13.7]
	3HP	13%	26%	1%	61%	38 [24-56]	27.9 [18.0-41.8]
Burundi	No TPT	0%	0%	0%	100%	1 [1-3]	18.8 [7.5-41.0]
	3HP	8%	8%	< 1%	84%	3 [2-5]	40.6 [21.7-68.0]
Brazil	No TPT	0%	0%	0%	100%	10 [5-19]	49.7 [22.9-91.8]
	3HP	5%	10%	< 1%	85%	16 [9-26]	77.6 [42.6-127.0]
Cambodia	No TPT	0%	0%	0%	100%	18 [9-32]	121.3 [58.7-213.6]
	3HP	3%	4%	< 1%	94%	22 [11-37]	144.5 [74.9-242.4]
Congo DRC	No TPT	0%	0%	0%	100%	30 [13-56]	12.9 [5.8-24.4]
	3HP	13%	20%	1%	66%	57 [36-85]	24.5 [15.7-36.7]
Eswatini	No TPT	0%	0%	0%	100%	6 [2-11]	277.8 [117.6-555.7]
	3HP	1%	6%	< 1%	93%	8 [4-14]	414.5 [206.2-713.9]
Ethiopia	No TPT	0%	0%	0%	100%	57 [27-101]	57.3 [27.3-101.5]
	3HP	5%	10%	< 1%	86%	75 [41-121]	75.0 [41.5-121.6]
Ghana	No TPT	0%	0%	0%	100%	2 [1-5]	21.8 [7.7-52.9]
	3HP	4%	19%	< 1%	77%	7 [4-12]	82.7 [45.4-136.4]
Haiti	No TPT	0%	0%	0%	100%	4 [2-8]	61.9 [28.1-116.1]
	3HP	4%	3%	< 1%	93%	6 [3-10]	81.9 [42.1-139.2]
India	No TPT	0%	0%	0%	100%	120 [54-229]	12.3 [5.5-23.6]
	3HP	12%	21%	< 1%	67%	340 [210-521]	35.0 [21.6-53.6]
Indonesia	No TPT	0%	0%	0%	100%	43 [21-76]	22.0 [10.5-38.6]
	3HP	9%	30%	< 1%	60%	89 [59-129]	45.1 [30.0-65.4]
Kenya	No TPT	0%	0%	0%	100%	32 [14-63]	59.8 [26.0-116.0]
	3HP	4%	13%	< 1%	82%	52 [29-84]	95.2 [52.7-155.7]
Lesotho	No TPT	0%	0%	0%	100%	2 [1-4]	48.9 [18.3-111.8]
	3HP	3%	8%	< 1%	88%	3 [2-6]	105.3 [52.9-182.6]
Liberia	No TPT	0%	0%	0%	100%	3 [1-5]	31.9 [14.5-59.5]
	3HP	7%	11%	< 1%	82%	4 [2-6]	46.6 [26.3-75.8]
Malawi	No TPT	0%	0%	0%	100%	8 [4-16]	49.5 [21.3-97.4]
	3HP	4%	7%	< 1%	88%	13 [7-22]	76.7 [40.1-129.4]
Mongolia	No TPT	0%	0%	0%	100%	4 [2-7]	185.3 [77.2-378.4]
	3HP	1%	4%	< 1%	95%	6 [3-10]	297.5 [145.1-515.3]
Mozambique	No TPT	0%	0%	0%	100%	26 [12-47]	27.3 [12.8-48.9]
	3HP	8%	12%	< 1%	80%	38 [22-61]	39.6 [22.9-62.9]
Namibia	No TPT	0%	0%	0%	100%	12 [6-21]	253.8 [121.1-451.3]
	3HP	2%	9%	< 1%	89%	15 [8-25]	326.7 [174.9-536.0]
Pakistan	No TPT	0%	0%	0%	100%	40 [19-74]	12.3 [5.7-22.7]
	3HP	15%	21%	1%	63%	75 [50-111]	23.1 [15.2-34.1]
Rwanda	No TPT	0%	0%	0%	100%	7 [4-12]	167.5 [81.9-290.3]
	3HP	2%	3%	< 1%	95%	8 [4-14]	196.9 [101.4-328.0]
Somalia	No TPT	0%	0%	0%	100%	8 [4-17]	39.0 [16.7-76.8]
	3HP	6%	6%	< 1%	87%	13 [7-22]	60.7 [32.2-101.5]
South Africa	No TPT	0%	0%	0%	100%	69 [32-123]	94.6 [44.3-169.6]
	3HP	5%	23%	< 1%	72%	109 [66-167]	149.6 [91.3-229.5]
Tajikistan	No TPT	0%	0%	0%	100%	3 [1-5]	57.8 [24.3-114.8]
	3HP	4%	3%	< 1%	93%	4 [2-7]	88.7 [44.4-151.6]
Tanzania	No TPT	0%	0%	0%	100%	49 [24-84]	56.1 [27.8-95.4]
	3HP	5%	12%	< 1%	83%	64 [36-101]	72.5 [41.6-114.8]
Thailand	No TPT	0%	0%	0%	100%	5 [2-10]	21.2 [8.4-44.7]
	3HP	10%	38%	< 1%	52%	14 [9-20]	62.3 [42.0-89.7]
Timor-Leste	No TPT	0%	0%	0%	100%	2 [1-4]	57.7 [27.0-104.2]
	3HP	5%	9%	< 1%	86%	3 [1-4]	77.6 [42.6-126.5]
Uganda	No TPT	0%	0%	0%	100%	24 [11-42]	38.0 [17.8-68.2]
	3HP	6%	12%	< 1%	81%	34 [19-54]	54.3 [31.2-86.5]
Zambia	No TPT	0%	0%	0%	100%	18 [8-37]	41.7 [17.4-83.4]
	3HP	5%	13%	< 1%	82%	31 [17-51]	70.1 [38.6-114.5]
Zimbabwe	No TPT	0%	0%	0%	100%	4 [2-9]	32.5 [13.2-66.9]
	3HP	8%	14%	< 1%	77%	8 [5-13]	63.7 [36.1-103.0]

Appendix Table 7: Costs of scaling up a 3HP program for household contacts five to fourteen years

Country	Scenario	3HP (% of total)	Contact Investigation (% of total)	Toxicity (% of total)	TB Treatment (% of total)	Total Cost (USD millions)	Cost per Contact (USD)
Bangladesh	No TPT	0%	0%	0%	100%	74 [28-153]	25.2 [9.4-51.8]
	3HP	13%	30%	< 1%	56%	131 [81-215]	44.6 [27.4-73.0]
Burundi	No TPT	0%	0%	0%	100%	5 [2-10]	34.9 [12.5-74.0]
	3HP	11%	20%	< 1%	68%	8 [4-13]	55.5 [29.7-99.5]
Brazil	No TPT	0%	0%	0%	100%	35 [13-73]	74.4 [27.5-153.6]
	3HP	7%	16%	< 1%	78%	46 [22-86]	97.0 [47.2-181.5]
Cambodia	No TPT	0%	0%	0%	100%	49 [19-100]	145.6 [55.3-297.2]
	3HP	4%	8%	< 1%	88%	53 [23-104]	155.9 [67.4-307.2]
Congo DRC	No TPT	0%	0%	0%	100%	72 [27-148]	17.7 [6.6-36.1]
	3HP	16%	36%	< 1%	47%	148 [98-227]	35.9 [23.8-55.4]
Eswatini	No TPT	0%	0%	0%	100%	14 [5-30]	357.2 [128.0-759.7]
	3HP	2%	9%	< 1%	89%	17 [7-35]	433.8 [179.8-873.0]
Ethiopia	No TPT	0%	0%	0%	100%	131 [49-268]	67.9 [25.5-138.9]
	3HP	7%	19%	< 1%	74%	167 [86-305]	86.6 [44.4-158.1]
Ghana	No TPT	0%	0%	0%	100%	8 [3-20]	49.7 [15.3-119.1]
	3HP	6%	25%	< 1%	68%	17 [9-32]	101.8 [52.7-188.2]
Haiti	No TPT	0%	0%	0%	100%	13 [5-26]	93.1 [35.5-188.5]
	3HP	6%	9%	< 1%	85%	14 [7-28]	103.1 [46.5-199.0]
India	No TPT	0%	0%	0%	100%	545 [197-1133]	29.0 [10.5-60.4]
	3HP	13%	27%	< 1%	60%	942 [554-1588]	50.2 [29.5-84.7]
Indonesia	No TPT	0%	0%	0%	100%	110 [41-226]	25.9 [9.7-53.2]
	3HP	12%	38%	< 1%	50%	226 [146-355]	53.2 [34.4-83.8]
Kenya	No TPT	0%	0%	0%	100%	66 [23-139]	69.4 [24.8-146.9]
	3HP	6%	22%	< 1%	72%	98 [51-179]	104.1 [53.9-189.6]
Lesotho	No TPT	0%	0%	0%	100%	5 [2-12]	77.5 [25.4-177.3]
	3HP	5%	15%	< 1%	80%	8 [4-17]	122.5 [56.0-241.4]
Liberia	No TPT	0%	0%	0%	100%	5 [2-10]	33.3 [11.9-70.5]
	3HP	11%	24%	< 1%	65%	8 [4-14]	54.0 [30.1-95.0]
Malawi	No TPT	0%	0%	0%	100%	19 [7-39]	67.1 [24.4-140.1]
	3HP	7%	16%	< 1%	77%	25 [12-48]	90.2 [44.0-169.7]
Mongolia	No TPT	0%	0%	0%	100%	18 [7-37]	322.7 [120.7-661.4]
	3HP	2%	6%	< 1%	92%	20 [8-40]	354.6 [144.2-713.5]
Mozambique	No TPT	0%	0%	0%	100%	57 [21-116]	33.6 [12.5-68.6]
	3HP	12%	25%	< 1%	62%	86 [49-147]	50.7 [29.2-86.7]
Namibia	No TPT	0%	0%	0%	100%	27 [10-55]	271.1 [101.2-557.8]
	3HP	2%	14%	< 1%	83%	32 [15-62]	325.7 [148.4-628.5]
Pakistan	No TPT	0%	0%	0%	100%	99 [36-204]	14.3 [5.2-29.5]
	3HP	19%	35%	1%	46%	215 [143-327]	31.1 [20.7-47.3]
Rwanda	No TPT	0%	0%	0%	100%	12 [4-25]	140.1 [50.6-296.4]
	3HP	4%	9%	< 1%	87%	15 [6-30]	175.6 [75.4-348.7]
Somalia	No TPT	0%	0%	0%	100%	19 [7-40]	53.7 [19.6-113.1]
	3HP	9%	17%	< 1%	74%	26 [13-48]	74.4 [37.6-138.1]
South Africa	No TPT	0%	0%	0%	100%	147 [54-306]	94.6 [34.9-197.3]
	3HP	5%	32%	< 1%	62%	241 [138-414]	155.4 [89.1-266.8]
Tajikistan	No TPT	0%	0%	0%	100%	10 [4-20]	89.6 [33.6-182.7]
	3HP	6%	9%	< 1%	84%	11 [5-22]	106.7 [48.1-206.0]
Tanzania	No TPT	0%	0%	0%	100%	80 [30-164]	53.6 [20.1-110.0]
	3HP	8%	23%	< 1%	68%	114 [62-202]	76.7 [41.7-135.8]
Thailand	No TPT	0%	0%	0%	100%	16 [6-33]	31.2 [11.2-66.0]
	3HP	10%	44%	< 1%	46%	37 [24-57]	73.5 [48.1-113.6]
Timor-Leste	No TPT	0%	0%	0%	100%	4 [1-8]	60.0 [21.9-124.8]
	3HP	8%	17%	< 1%	75%	6 [3-10]	82.1 [41.2-152.3]
Uganda	No TPT	0%	0%	0%	100%	54 [20-111]	44.8 [16.9-91.8]
	3HP	10%	23%	< 1%	67%	78 [43-137]	65.0 [36.0-113.8]
Zambia	No TPT	0%	0%	0%	100%	39 [14-82]	52.6 [19.0-110.7]
	3HP	8%	23%	< 1%	69%	60 [32-108]	81.3 [43.2-146.2]
Zimbabwe	No TPT	0%	0%	0%	100%	12 [4-25]	43.2 [15.2-92.2]
	3HP	10%	25%	< 1%	65%	20 [11-35]	73.7 [40.5-129.4]

Appendix Table 8: Costs of scaling up a 3HP program for household contacts fifteen years and above

Country	Scenario	3HP (% of total)	Contact Investigation (% of total)	Toxicity (% of total)	TB Treatment (% of total)	Total Cost (USD millions)	Cost per Contact (USD)
Bangladesh	No TPT	0%	0%	0%	100%	158 [86-258]	14.8 [8.0-24.2]
	3HP	21%	36%	2%	41%	345 [272-436]	32.5 [25.6-41.1]
Burundi	No TPT	0%	0%	0%	100%	6 [3-10]	23.8 [12.8-38.9]
	3HP	18%	24%	1%	57%	10 [7-13]	38.2 [28.3-51.1]
Brazil	No TPT	0%	0%	0%	100%	126 [69-206]	50.3 [27.4-82.1]
	3HP	11%	20%	1%	69%	163 [113-229]	64.9 [45.2-91.2]
Cambodia	No TPT	0%	0%	0%	100%	79 [42-131]	68.4 [36.7-113.8]
	3HP	9%	12%	1%	79%	92 [60-136]	80.2 [52.4-118.0]
Congo DRC	No TPT	0%	0%	0%	100%	65 [35-106]	9.3 [5.1-15.2]
	3HP	25%	42%	2%	31%	187 [152-228]	26.8 [21.9-32.8]
Eswatini	No TPT	0%	0%	0%	100%	23 [12-37]	231.8 [123.7-383.5]
	3HP	3%	13%	< 1%	83%	25 [16-37]	258.8 [164.2-384.8]
Ethiopia	No TPT	0%	0%	0%	100%	142 [77-234]	36.5 [19.8-60.1]
	3HP	13%	26%	1%	60%	208 [151-285]	53.5 [38.7-73.2]
Ghana	No TPT	0%	0%	0%	100%	18 [9-32]	43.6 [21.1-77.8]
	3HP	9%	31%	1%	59%	31 [22-43]	74.7 [53.6-103.3]
Haiti	No TPT	0%	0%	0%	100%	20 [11-32]	43.2 [23.3-70.8]
	3HP	13%	13%	1%	73%	24 [16-35]	53.5 [36.2-76.4]
India	No TPT	0%	0%	0%	100%	1618 [882-2644]	18.8 [10.3-30.8]
	3HP	19%	32%	2%	47%	3145 [2408-4077]	36.6 [28.1-47.5]
Indonesia	No TPT	0%	0%	0%	100%	252 [137-413]	13.4 [7.3-22.0]
	3HP	18%	47%	1%	33%	741 [589-925]	39.5 [31.4-49.3]
Kenya	No TPT	0%	0%	0%	100%	117 [63-193]	46.6 [25.1-76.6]
	3HP	10%	29%	1%	60%	178 [129-243]	70.9 [51.3-96.5]
Lesotho	No TPT	0%	0%	0%	100%	10 [5-18]	60.7 [30.0-106.5]
	3HP	8%	18%	1%	73%	14 [9-21]	83.2 [55.5-121.4]
Liberia	No TPT	0%	0%	0%	100%	6 [3-10]	19.3 [10.0-32.7]
	3HP	18%	30%	1%	51%	11 [9-15]	36.5 [27.6-48.1]
Malawi	No TPT	0%	0%	0%	100%	25 [13-41]	42.0 [22.7-68.8]
	3HP	12%	20%	1%	67%	34 [24-47]	57.3 [40.4-79.7]
Mongolia	No TPT	0%	0%	0%	100%	20 [9-36]	134.0 [62.5-243.7]
	3HP	5%	10%	< 1%	85%	25 [15-38]	167.7 [102.4-258.2]
Mozambique	No TPT	0%	0%	0%	100%	61 [33-100]	19.1 [10.4-31.2]
	3HP	20%	31%	1%	48%	112 [86-145]	34.7 [26.6-45.1]
Namibia	No TPT	0%	0%	0%	100%	41 [22-68]	153.0 [81.9-254.5]
	3HP	4%	22%	< 1%	74%	51 [35-74]	191.5 [128.8-275.2]
Pakistan	No TPT	0%	0%	0%	100%	134 [70-227]	7.4 [3.9-12.5]
	3HP	29%	39%	2%	30%	430 [350-527]	23.6 [19.3-29.0]
Rwanda	No TPT	0%	0%	0%	100%	21 [11-33]	104.5 [57.2-169.2]
	3HP	6%	12%	< 1%	82%	22 [14-33]	113.9 [73.4-167.3]
Somalia	No TPT	0%	0%	0%	100%	24 [12-41]	27.4 [13.9-47.3]
	3HP	16%	23%	1%	60%	38 [27-53]	44.6 [32.0-61.6]
South Africa	No TPT	0%	0%	0%	100%	377 [205-618]	62.5 [34.0-102.6]
	3HP	8%	42%	1%	50%	684 [511-903]	113.5 [84.9-149.8]
Tajikistan	No TPT	0%	0%	0%	100%	14 [7-23]	46.9 [25.0-77.5]
	3HP	12%	13%	1%	74%	18 [12-25]	59.8 [40.3-85.8]
Tanzania	No TPT	0%	0%	0%	100%	94 [51-153]	29.6 [16.0-48.3]
	3HP	14%	31%	1%	54%	157 [118-208]	49.6 [37.1-65.7]
Thailand	No TPT	0%	0%	0%	100%	70 [39-114]	25.9 [14.3-42.0]
	3HP	13%	49%	1%	37%	170 [131-217]	62.5 [48.2-80.1]
Timor-Leste	No TPT	0%	0%	0%	100%	6 [3-10]	34.1 [17.9-57.2]
	3HP	14%	23%	1%	62%	9 [6-12]	51.0 [36.4-70.5]
Uganda	No TPT	0%	0%	0%	100%	53 [28-88]	22.6 [11.9-37.7]
	3HP	17%	32%	1%	51%	98 [74-128]	41.9 [31.8-55.0]
Zambia	No TPT	0%	0%	0%	100%	52 [29-85]	36.7 [20.1-59.8]
	3HP	12%	29%	1%	58%	80 [59-107]	56.1 [41.3-75.4]
Zimbabwe	No TPT	0%	0%	0%	100%	22 [12-37]	32.7 [17.6-54.2]
	3HP	14%	30%	1%	54%	38 [28-50]	55.9 [41.0-75.0]

Appendix Table 9: Costs of scaling up a 3HP program for people living with HIV/AIDS

Country	Scenario	3HP (% of total)	ART (% of total)	Toxicity (% of total)	TB Treatment (% of total)	Total Cost (USD millions)	Cost per PLWHA (USD)
Bangladesh	No TPT	0%	99%	0%	1%	314.6 [273.1-365.7]	999.3 [867.5-1161.7]
	3HP	1%	97%	< 1%	1%	319.5 [277.5-371.4]	1014.9 [881.6-1179.8]
Burundi	No TPT	0%	98%	0%	2%	72.1 [64.0-80.6]	934.7 [829.4-1044.3]
	3HP	1%	97%	< 1%	1%	73.1 [64.9-81.6]	947.8 [841.8-1058.3]
Brazil	No TPT	0%	98%	0%	2%	1007.4 [885.5-1165.6]	839.1 [737.6-970.8]
	3HP	2%	96%	< 1%	2%	1025.2 [902.3-1186.4]	853.9 [751.6-988.2]
Cambodia	No TPT	0%	90%	0%	10%	59.6 [51.4-70.0]	1051.7 [906.6-1234.2]
	3HP	1%	91%	< 1%	8%	59.5 [51.5-69.7]	1049.5 [908.5-1229.2]
Congo DRC	No TPT	0%	99%	0%	1%	323.0 [287.2-361.1]	881.1 [783.4-985.0]
	3HP	2%	97%	< 1%	1%	328.4 [292.3-366.8]	895.8 [797.3-1000.6]
Eswatini	No TPT	0%	83%	0%	17%	121.6 [99.9-152.3]	1163.7 [956.6-1458.3]
	3HP	1%	84%	< 1%	14%	119.6 [99.2-149.7]	1145.1 [949.9-1433.0]
Ethiopia	No TPT	0%	97%	0%	3%	291.4 [257.5-329.1]	906.4 [801.0-1023.6]
	3HP	2%	96%	< 1%	2%	295.3 [261.3-333.4]	918.6 [812.6-1036.9]
Ghana	No TPT	0%	94%	0%	6%	291.0 [258.1-328.5]	844.8 [749.3-953.7]
	3HP	2%	94%	< 1%	5%	293.5 [261.0-330.7]	852.0 [757.7-960.0]
Haiti	No TPT	0%	95%	0%	5%	29.1 [26.1-32.7]	705.0 [633.2-792.2]
	3HP	2%	94%	< 1%	4%	29.5 [26.5-33.1]	715.1 [643.5-803.0]
India	No TPT	0%	98%	0%	2%	906.5 [784.8-1078.9]	908.2 [786.2-1080.9]
	3HP	2%	97%	< 1%	1%	922.8 [799.4-1099.2]	924.5 [800.8-1101.2]
Indonesia	No TPT	0%	97%	0%	3%	208.2 [180.6-251.6]	785.5 [681.1-949.2]
	3HP	2%	95%	1%	2%	212.3 [184.1-256.7]	800.7 [694.6-968.5]
Kenya	No TPT	0%	98%	0%	2%	825.4 [718.8-965.2]	924.9 [805.4-1081.5]
	3HP	2%	97%	< 1%	1%	838.7 [730.7-981.1]	939.8 [818.8-1099.4]
Lesotho	No TPT	0%	93%	0%	7%	227.4 [199.5-259.1]	981.7 [861.1-1118.4]
	3HP	1%	92%	< 1%	6%	228.2 [200.7-259.1]	984.8 [866.3-1118.4]
Liberia	No TPT	0%	98%	0%	2%	19.8 [17.8-22.0]	816.9 [732.2-905.2]
	3HP	2%	97%	< 1%	2%	20.1 [18.1-22.3]	829.6 [744.4-918.5]
Malawi	No TPT	0%	97%	0%	3%	189.6 [170.3-210.6]	788.1 [707.6-875.2]
	3HP	2%	96%	< 1%	2%	192.4 [173.0-213.5]	799.5 [718.8-887.2]
Mongolia	No TPT	0%	79%	0%	21%	0.4 [0.4-0.5]	1120.2 [917.2-1420.9]
	3HP	1%	81%	< 1%	18%	0.4 [0.3-0.5]	1091.4 [908.2-1360.0]
Mozambique	No TPT	0%	98%	0%	2%	902.5 [814.4-1000.2]	713.9 [644.3-791.2]
	3HP	2%	96%	< 1%	2%	918.8 [830.0-1017.4]	726.8 [656.6-804.8]
Namibia	No TPT	0%	81%	0%	19%	55.4 [44.9-71.3]	981.5 [794.3-1262.4]
	3HP	2%	82%	1%	15%	54.7 [44.7-70.5]	968.5 [791.5-1247.9]
Pakistan	No TPT	0%	99%	0%	1%	214.8 [195.6-239.8]	502.7 [457.9-561.2]
	3HP	3%	96%	1%	1%	221.5 [202.0-247.2]	518.3 [472.7-578.6]
Rwanda	No TPT	0%	98%	0%	2%	200.5 [176.9-226.6]	950.4 [838.5-1073.7]
	3HP	1%	97%	< 1%	1%	203.3 [179.5-229.6]	963.5 [850.8-1088.3]
Somalia	No TPT	0%	97%	0%	3%	3.4 [3.1-3.9]	646.2 [577.8-737.3]
	3HP	2%	95%	< 1%	3%	3.5 [3.1-4.0]	659.0 [590.0-752.3]
South Africa	No TPT	0%	95%	0%	5%	4465.8 [3446.1-6486.5]	1058.6 [816.9-1537.6]
	3HP	2%	93%	1%	4%	4548.2 [3509.6-6621.6]	1078.1 [831.9-1569.6]
Tajikistan	No TPT	0%	96%	0%	4%	7.1 [6.3-7.9]	782.8 [699.8-877.0]
	3HP	2%	95%	< 1%	3%	7.2 [6.4-8.0]	793.0 [710.3-888.0]
Tanzania	No TPT	0%	97%	0%	3%	945.9 [839.6-1072.3]	833.4 [739.8-944.7]
	3HP	2%	96%	< 1%	2%	959.5 [852.7-1087.7]	845.4 [751.3-958.3]
Thailand	No TPT	0%	98%	0%	2%	467.8 [369.9-646.0]	1162.7 [919.4-1605.8]
	3HP	1%	96%	1%	1%	477.2 [377.3-659.5]	1186.1 [937.8-1639.3]
Timor-Leste	No TPT	0%	93%	0%	7%	1.7 [1.6-2.0]	695.1 [617.7-793.5]
	3HP	2%	92%	< 1%	5%	1.8 [1.6-2.0]	702.5 [626.1-801.8]
Uganda	No TPT	0%	99%	0%	1%	1375.0 [1212.8-1555.6]	939.7 [828.8-1063.1]
	3HP	1%	97%	< 1%	1%	1396.9 [1233.4-1579.8]	954.6 [842.9-1079.6]
Zambia	No TPT	0%	96%	0%	4%	1392.6 [1237.5-1567.9]	845.8 [751.6-952.2]
	3HP	2%	95%	< 1%	3%	1409.7 [1254.2-1586.0]	856.2 [761.7-963.2]
Zimbabwe	No TPT	0%	98%	0%	2%	446.0 [369.7-576.0]	988.5 [819.3-1276.4]
	3HP	2%	96%	1%	2%	455.0 [377.3-588.1]	1008.3 [836.2-1303.3]

Appendix Table 10: Costs of scaling up a 3HP program for people living with HIV/AIDS, excluding ART

Country	Scenario	3HP (% of total)	Toxicity (% of total)	TB Treatment (% of total)	Total Cost (USD millions)	Cost per PLWHA (USD)
Bangladesh	No TPT	0%	0%	100%	4.0 [1.7-7.4]	12.6 [5.4-23.4]
	3HP	54%	11%	36%	8.6 [6.6-11.5]	27.3 [20.9-36.6]
Burundi	No TPT	0%	0%	100%	1.1 [0.5-2.0]	14.2 [6.1-26.1]
	3HP	52%	7%	41%	2.1 [1.6-2.8]	26.8 [20.2-36.6]
Brazil	No TPT	0%	0%	100%	23.4 [9.8-44.4]	19.5 [8.2-37.0]
	3HP	44%	11%	45%	40.7 [29.0-58.0]	33.9 [24.1-48.3]
Cambodia	No TPT	0%	0%	100%	5.7 [2.5-10.4]	101.0 [44.4-183.8]
	3HP	16%	3%	81%	5.5 [3.0-9.4]	97.4 [52.3-165.8]
Congo DRC	No TPT	0%	0%	100%	3.3 [1.5-5.9]	8.9 [4.0-16.0]
	3HP	61%	9%	30%	8.3 [6.8-10.5]	22.7 [18.6-28.6]
Eswatini	No TPT	0%	0%	100%	21.1 [9.6-37.6]	202.3 [91.8-359.7]
	3HP	9%	3%	87%	19.1 [9.8-32.5]	182.8 [94.0-311.4]
Ethiopia	No TPT	0%	0%	100%	7.5 [3.3-13.7]	23.2 [10.3-42.7]
	3HP	42%	7%	51%	11.2 [7.8-16.4]	34.8 [24.2-50.9]
Ghana	No TPT	0%	0%	100%	16.8 [7.6-30.1]	48.7 [22.1-87.5]
	3HP	27%	5%	69%	19.0 [11.6-29.8]	55.2 [33.7-86.5]
Haiti	No TPT	0%	0%	100%	1.5 [0.7-2.6]	35.4 [16.3-62.1]
	3HP	33%	6%	61%	1.8 [1.2-2.7]	44.8 [29.5-66.3]
India	No TPT	0%	0%	100%	15.4 [7.0-27.5]	15.5 [7.0-27.5]
	3HP	49%	13%	39%	31.0 [23.3-41.4]	31.1 [23.3-41.5]
Indonesia	No TPT	0%	0%	100%	6.6 [3.1-11.3]	24.9 [11.6-42.8]
	3HP	39%	11%	50%	10.3 [7.3-14.2]	38.7 [27.6-53.5]
Kenya	No TPT	0%	0%	100%	14.9 [6.7-26.8]	16.7 [7.5-30.0]
	3HP	48%	10%	41%	27.9 [20.7-38.0]	31.3 [23.2-42.6]
Lesotho	No TPT	0%	0%	100%	16.6 [7.5-29.9]	71.8 [32.2-129.2]
	3HP	20%	4%	76%	17.2 [9.8-28.1]	74.0 [42.2-121.2]
Liberia	No TPT	0%	0%	100%	0.4 [0.2-0.7]	16.6 [7.6-29.8]
	3HP	48%	7%	45%	0.7 [0.5-1.0]	28.7 [21.4-39.4]
Malawi	No TPT	0%	0%	100%	5.9 [2.7-10.3]	24.4 [11.2-42.7]
	3HP	40%	6%	53%	8.5 [6.0-12.1]	35.4 [24.8-50.2]
Mongolia	No TPT	0%	0%	100%	0.1 [0.0-0.2]	245.9 [102.8-502.6]
	3HP	8%	2%	90%	0.1 [0.0-0.2]	215.9 [100.7-427.1]
Mozambique	No TPT	0%	0%	100%	22.1 [10.3-38.3]	17.5 [8.2-30.3]
	3HP	47%	8%	46%	37.5 [28.1-50.3]	29.6 [22.2-39.8]
Namibia	No TPT	0%	0%	100%	10.6 [5.0-18.3]	187.8 [88.2-323.9]
	3HP	10%	5%	85%	9.8 [5.3-16.1]	173.8 [93.8-284.2]
Pakistan	No TPT	0%	0%	100%	3.0 [1.4-5.1]	7.0 [3.3-12.0]
	3HP	63%	13%	24%	9.4 [7.9-11.3]	22.1 [18.6-26.6]
Rwanda	No TPT	0%	0%	100%	3.7 [1.6-6.8]	17.3 [7.4-32.4]
	3HP	48%	8%	44%	6.4 [4.6-9.0]	30.3 [22.0-42.9]
Somalia	No TPT	0%	0%	100%	0.1 [0.1-0.2]	21.5 [10.0-37.5]
	3HP	43%	8%	49%	0.2 [0.1-0.2]	33.9 [24.5-46.8]
South Africa	No TPT	0%	0%	100%	222.6 [101.5-393.9]	52.8 [24.0-93.4]
	3HP	27%	16%	58%	300.9 [190.6-451.6]	71.3 [45.2-107.0]
Tajikistan	No TPT	0%	0%	100%	0.3 [0.1-0.5]	33.4 [15.2-59.8]
	3HP	34%	6%	60%	0.4 [0.3-0.6]	43.1 [28.4-64.5]
Tanzania	No TPT	0%	0%	100%	28.6 [13.2-49.9]	25.2 [11.7-44.0]
	3HP	39%	7%	53%	41.5 [29.1-58.8]	36.5 [25.6-51.8]
Thailand	No TPT	0%	0%	100%	8.5 [3.6-16.0]	21.3 [9.1-39.7]
	3HP	41%	21%	38%	17.5 [12.0-25.3]	43.6 [29.8-62.8]
Timor-Leste	No TPT	0%	0%	100%	0.1 [0.1-0.2]	48.4 [22.6-83.5]
	3HP	27%	6%	68%	0.1 [0.1-0.2]	54.8 [34.6-82.6]
Uganda	No TPT	0%	0%	100%	13.8 [5.9-25.8]	9.4 [4.0-17.7]
	3HP	59%	10%	30%	35.1 [28.1-45.3]	24.0 [19.2-31.0]
Zambia	No TPT	0%	0%	100%	50.1 [22.7-89.5]	30.5 [13.8-54.4]
	3HP	36%	6%	58%	66.2 [44.4-97.6]	40.2 [26.9-59.2]
Zimbabwe	No TPT	0%	0%	100%	8.7 [3.9-15.7]	19.4 [8.7-34.8]
	3HP	43%	18%	39%	17.4 [12.4-24.2]	38.6 [27.6-53.6]

Appendix Figure 3: Average annual cost of a 3HP program, as a share of countries' annual TB budgets

	Contacts < 5	Contacts 5-14	Contacts 15+	PWH	Total
Bangladesh	2.3% [1.2 - 4.0%]	4.6% [2.8 - 6.9%]	15.2% [9.7 - 20.9%]	0.4% [0.2 - 0.5%]	22.5% [13.9 - 32.3%]
Brazil	1.6% [0.5 - 3.2%]	3.0% [1.2 - 5.3%]	10.2% [2.3 - 17.0%]	4.8% [2.4 - 7.5%]	19.6% [6.5 - 33.0%]
Burundi	6.8% [2.6 - 13.0%]	12.2% [5.7 - 21.0%]	16.0% [7.8 - 23.8%]	4.2% [2.7 - 5.6%]	39.3% [18.8 - 63.4%]
Cambodia	1.5% [-0.3 - 4.4%]	1.5% [-0.9 - 3.6%]	6.0% [-1.7 - 12.5%]	-0.1% [-0.6 - 0.4%]	9.0% [-3.6 - 20.9%]
Congo DRC	5.9% [2.8 - 10.2%]	16.5% [10.7 - 23.0%]	26.8% [18.8 - 35.3%]	1.1% [0.8 - 1.4%]	50.3% [33.1 - 69.9%]
Eswatini	2.2% [0.3 - 4.9%]	2.4% [0.2 - 5.6%]	2.1% [-1.7 - 5.3%]	-1.6% [-5.1 - 1.3%]	5.1% [-6.2 - 17.1%]
Ethiopia	2.8% [0.4 - 6.4%]	5.8% [2.4 - 9.0%]	10.6% [4.3 - 16.2%]	0.6% [0.3 - 0.9%]	19.8% [7.3 - 32.6%]
Ghana	6.2% [2.9 - 11.1%]	10.3% [5.0 - 18.5%]	15.1% [7.4 - 22.6%]	2.6% [-1.7 - 6.3%]	34.3% [13.5 - 58.5%]
Haiti	1.6% [0.1 - 3.8%]	1.6% [-0.2 - 3.2%]	5.3% [0.3 - 9.6%]	0.4% [0.1 - 0.8%]	8.9% [0.2 - 17.3%]
India	3.1% [1.5 - 5.5%]	5.6% [3.2 - 8.9%]	21.6% [12.8 - 31.0%]	0.2% [0.1 - 0.3%]	30.5% [17.6 - 45.8%]
Indonesia	2.0% [1.0 - 3.4%]	5.1% [3.3 - 7.4%]	21.4% [14.7 - 29.1%]	0.2% [0.1 - 0.3%]	28.7% [19.1 - 40.2%]
Kenya	3.5% [1.0 - 7.1%]	6.0% [2.8 - 10.4%]	11.2% [4.9 - 17.0%]	2.4% [1.4 - 3.6%]	23.0% [10.1 - 38.1%]
Lesotho	1.4% [0.5 - 2.6%]	2.2% [0.8 - 4.4%]	2.8% [0.7 - 4.7%]	0.4% [-2.2 - 2.5%]	6.7% [-0.2 - 14.3%]
Liberia	2.7% [0.7 - 5.6%]	6.7% [3.6 - 10.9%]	12.2% [7.1 - 17.3%]	0.7% [0.4 - 0.9%]	22.3% [11.8 - 34.7%]
Malawi	1.8% [0.4 - 3.8%]	2.5% [1.0 - 4.6%]	3.5% [1.0 - 5.7%]	1.0% [0.5 - 1.5%]	8.8% [2.8 - 15.6%]
Mongolia	2.4% [0.5 - 5.2%]	1.9% [-0.7 - 5.2%]	5.4% [-0.8 - 11.2%]	-0.0% [-0.0 - 0.0%]	9.7% [-1.1 - 21.5%]
Mozambique	3.1% [0.9 - 6.2%]	7.5% [4.1 - 10.9%]	13.0% [7.3 - 18.3%]	4.0% [2.4 - 5.5%]	27.4% [14.8 - 40.9%]
Namibia	2.5% [0.1 - 6.1%]	4.0% [0.9 - 7.9%]	7.7% [0.0 - 14.4%]	-0.6% [-2.1 - 0.9%]	13.6% [-1.1 - 29.3%]
Pakistan	4.2% [2.0 - 7.4%]	14.0% [9.0 - 20.1%]	35.4% [25.2 - 46.8%]	0.8% [0.6 - 1.0%]	54.4% [36.8 - 75.3%]
Rwanda	1.3% [-0.4 - 3.9%]	3.2% [0.5 - 7.0%]	1.9% [-2.4 - 5.3%]	2.9% [1.6 - 4.0%]	9.3% [-0.6 - 20.2%]
Somalia	3.1% [0.7 - 6.6%]	4.7% [1.9 - 8.4%]	9.6% [4.3 - 15.0%]	0.0% [0.0 - 0.1%]	17.5% [6.9 - 30.0%]
South Africa	1.4% [0.5 - 2.6%]	3.2% [1.8 - 5.2%]	10.5% [5.4 - 15.8%]	2.7% [-0.4 - 7.0%]	17.8% [7.3 - 30.7%]
Tajikistan	1.0% [0.2 - 2.3%]	1.4% [0.2 - 2.8%]	2.9% [0.5 - 5.1%]	0.1% [0.0 - 0.1%]	5.5% [0.9 - 10.3%]
Tanzania	3.3% [0.6 - 7.7%]	7.9% [4.1 - 12.4%]	14.6% [7.7 - 21.3%]	3.0% [1.2 - 4.7%]	28.9% [13.5 - 46.0%]
Thailand	2.4% [1.2 - 4.0%]	5.5% [3.4 - 8.4%]	25.4% [15.3 - 36.9%]	2.3% [0.8 - 4.5%]	35.5% [20.7 - 53.9%]
Timor-Leste	1.8% [0.2 - 4.0%]	3.9% [1.6 - 6.8%]	7.3% [3.0 - 11.4%]	0.0% [-0.0 - 0.1%]	13.0% [4.9 - 22.4%]
Uganda	2.6% [0.7 - 5.4%]	6.3% [3.3 - 9.6%]	11.6% [6.6 - 16.4%]	5.5% [3.9 - 7.1%]	25.9% [14.6 - 38.5%]
Zambia	3.1% [1.0 - 6.1%]	5.2% [2.6 - 8.8%]	6.8% [3.0 - 10.1%]	3.9% [0.9 - 6.7%]	19.0% [7.4 - 31.7%]
Zimbabwe	1.9% [0.7 - 3.9%]	3.9% [2.0 - 6.9%]	7.5% [3.6 - 11.8%]	4.1% [1.9 - 7.4%]	17.4% [8.1 - 29.9%]
**Median (IQR)**	2.4% [1.8 - 3.1%]	4.7% [3.0 - 6.3%]	10.5% [6.0 - 15.1%]	0.8% [0.2 - 2.9%]	19.6% [9.7 - 28.7%]

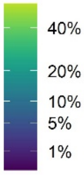

Numbers shown in the figure were calculated as the cumulative costs under the 3HP scenario minus the cumulative costs under the no TPT scenario divided by thirteen years (to calculate average annual costs), divided by countries' total expected TB funding in 2021 (as reported to the WHO). For people with HIV (PWH), ART costs are not included as these costs would likely not be borne by the National TB Program. Numbers indicate means with 2·5th and 97·5th uncertainty intervals in brackets (except the last row, which shows medians and interquartile ranges across country means). Household contacts ages are displayed in years.

Appendix Table 11: Total projected TB cases with and without 3HP

Country	Scenario	Household contacts < 5	Household contacts 5-14	Household contacts 15+	People living with HIV/AIDS
Bangladesh	No TPT	219.6 [119.8-345.2]	376.4 [149.3-737.6]	694.2 [408.9-1067.0]	13.2 [6.0-23.5]
	3HP	193.9 [103.0-311.8]	346.0 [127.9-702.9]	585.4 [353.8-880.1]	10.4 [4.6-19.0]
	Cases averted by 3HP	25.6 [10.3-47.1]	30.4 [11.1-59.5]	108.8 [39.6-213.1]	2.8 [1.0-5.3]
Brazil	No TPT	34.0 [18.5-53.4]	60.9 [24.2-119.4]	164.4 [96.8-252.7]	25.4 [11.2-46.0]
	3HP	30.0 [15.9-48.3]	56.0 [20.7-113.8]	138.8 [83.9-208.7]	19.9 [8.8-36.6]
	Cases averted by 3HP	4.0 [1.6-7.3]	4.9 [1.8-9.6]	25.6 [9.3-50.1]	5.4 [2.2-10.3]
Burundi	No TPT	11.6 [6.3-18.3]	17.2 [6.8-33.8]	16.4 [9.7-25.2]	2.5 [1.1-4.5]
	3HP	10.2 [5.4-16.4]	15.8 [5.8-32.2]	13.8 [8.3-20.7]	2.0 [0.9-3.6]
	Cases averted by 3HP	1.4 [0.6-2.6]	1.4 [0.5-2.8]	2.6 [1.0-5.2]	0.5 [0.2-1.0]
Cambodia	No TPT	24.6 [13.4-38.7]	43.2 [17.1-84.7]	75.0 [44.2-115.3]	4.9 [2.2-8.5]
	3HP	21.7 [11.5-34.9]	39.7 [14.7-80.7]	63.2 [38.2-95.0]	3.8 [1.8-6.8]
	Cases averted by 3HP	2.9 [1.2-5.3]	3.5 [1.3-6.9]	11.8 [4.3-23.1]	1.0 [0.4-1.9]
Congo DRC	No TPT	373.5 [203.2-588.1]	521.4 [205.8-1024.7]	449.7 [265.1-690.2]	16.3 [7.7-28.0]
	3HP	328.0 [173.2-528.6]	478.0 [175.0-975.8]	377.3 [228.2-566.9]	12.9 [6.0-22.4]
	Cases averted by 3HP	45.4 [18.1-83.5]	43.4 [15.8-85.1]	72.4 [26.2-141.9]	3.4 [1.4-6.2]
Eswatini	No TPT	3.3 [1.8-5.1]	5.1 [2.0-9.9]	6.3 [3.7-9.7]	5.1 [2.4-8.7]
	3HP	2.9 [1.5-4.6]	4.7 [1.7-9.5]	5.3 [3.2-8.0]	4.0 [1.9-7.0]
	Cases averted by 3HP	0.4 [0.2-0.7]	0.4 [0.1-0.8]	1.0 [0.4-2.0]	1.1 [0.4-1.9]
Ethiopia	No TPT	162.2 [88.3-255.3]	245.6 [97.2-482.2]	252.9 [149.0-388.3]	12.0 [5.7-20.7]
	3HP	142.8 [75.6-229.7]	225.3 [82.7-459.3]	212.4 [128.5-319.4]	9.5 [4.5-16.6]
	Cases averted by 3HP	19.4 [7.8-35.6]	20.3 [7.4-39.9]	40.4 [14.7-79.2]	2.5 [1.0-4.6]
Ghana	No TPT	14.2 [7.7-22.3]	21.4 [8.5-42.1]	27.0 [15.9-41.4]	15.2 [7.3-26.0]
	3HP	12.5 [6.6-20.0]	19.7 [7.2-40.0]	22.7 [13.7-34.1]	12.0 [5.7-20.8]
	Cases averted by 3HP	1.7 [0.7-3.1]	1.8 [0.6-3.5]	4.3 [1.6-8.4]	3.2 [1.4-5.8]
Haiti	No TPT	11.1 [6.1-17.5]	17.9 [7.1-35.1]	29.5 [17.4-45.3]	1.9 [0.9-3.2]
	3HP	9.8 [5.2-15.8]	16.4 [6.1-33.4]	24.9 [15.0-37.4]	1.5 [0.7-2.5]
	Cases averted by 3HP	1.3 [0.5-2.4]	1.5 [0.5-2.8]	4.6 [1.7-9.1]	0.4 [0.2-0.7]
India	No TPT	1581.2 [862.0-2487.5]	2394.9 [949.3-4696.0]	5608.3 [3303.8-8620.2]	43.6 [21.0-73.7]
	3HP	1393.4 [738.4-2241.0]	2201.1 [812.1-4474.1]	4730.9 [2859.0-7113.0]	34.4 [16.4-59.0]
	Cases averted by 3HP	187.8 [75.1-344.6]	193.7 [70.6-379.3]	877.4 [319.3-1718.1]	9.2 [3.9-16.4]
Indonesia	No TPT	321.7 [175.4-506.2]	542.1 [215.0-1062.6]	1226.6 [722.5-1885.3]	21.9 [10.8-35.9]
	3HP	283.4 [150.1-455.8]	498.4 [184.1-1012.6]	1034.4 [625.1-1555.2]	17.2 [8.5-28.6]
	Cases averted by 3HP	38.3 [15.3-70.3]	43.7 [15.9-85.6]	192.1 [69.9-376.3]	4.6 [2.2-7.9]
Kenya	No TPT	87.9 [47.9-138.4]	120.3 [47.6-236.0]	163.1 [96.1-250.5]	17.7 [8.4-30.4]
	3HP	77.2 [40.8-124.4]	110.5 [40.7-224.9]	137.1 [82.9-206.1]	13.9 [6.6-24.3]
	Cases averted by 3HP	10.7 [4.3-19.6]	9.8 [3.6-19.2]	26.0 [9.4-51.0]	3.7 [1.5-6.8]
Lesotho	No TPT	5.4 [2.9-8.5]	8.8 [3.5-17.2]	11.1 [6.5-17.0]	12.6 [5.9-21.6]
	3HP	4.7 [2.5-7.6]	8.1 [3.0-16.4]	9.3 [5.6-14.0]	9.9 [4.7-17.4]
	Cases averted by 3HP	0.6 [0.3-1.2]	0.7 [0.3-1.4]	1.7 [0.6-3.4]	2.6 [1.1-4.8]
Liberia	No TPT	13.3 [7.2-20.9]	18.3 [7.2-35.9]	20.4 [12.0-31.3]	0.9 [0.4-1.6]
	3HP	11.7 [6.2-18.8]	16.8 [6.2-34.2]	17.2 [10.4-25.8]	0.7 [0.3-1.3]
	Cases averted by 3HP	1.6 [0.6-3.0]	1.5 [0.5-3.0]	3.3 [1.2-6.4]	0.2 [0.1-0.3]
Malawi	No TPT	27.3 [14.9-43.0]	35.8 [14.1-70.3]	38.3 [22.6-58.7]	8.1 [3.9-13.5]
	3HP	24.0 [12.7-38.7]	32.8 [12.0-67.0]	32.1 [19.4-48.3]	6.4 [3.1-10.8]
	Cases averted by 3HP	3.3 [1.3-6.1]	3.0 [1.1-5.8]	6.1 [2.2-12.0]	1.7 [0.8-2.9]
Mongolia	No TPT	3.2 [1.7-5.0]	7.2 [2.8-14.0]	9.6 [5.7-14.8]	0.0 [0.0-0.0]
	3HP	2.8 [1.5-4.5]	6.6 [2.4-13.4]	8.1 [4.9-12.2]	0.0 [0.0-0.0]
	Cases averted by 3HP	0.4 [0.1-0.7]	0.6 [0.2-1.1]	1.5 [0.6-3.0]	0.0 [0.0-0.0]
Mozambique	No TPT	156.1 [85.0-245.8]	214.9 [84.8-422.4]	207.9 [122.5-319.0]	76.9 [38.0-127.0]
	3HP	137.2 [72.4-221.0]	197.0 [72.1-402.2]	174.5 [105.6-262.3]	60.6 [29.7-100.9]
	Cases averted by 3HP	19.0 [7.6-34.8]	17.9 [6.5-35.1]	33.3 [12.1-65.3]	16.3 [7.7-27.8]
Namibia	No TPT	7.6 [4.1-11.9]	12.6 [5.0-24.7]	17.4 [10.3-26.8]	3.8 [1.9-6.3]
	3HP	6.7 [3.5-10.7]	11.6 [4.3-23.5]	14.7 [8.9-22.1]	3.0 [1.5-5.0]
	Cases averted by 3HP	0.9 [0.4-1.7]	1.0 [0.4-2.0]	2.8 [1.0-5.4]	0.8 [0.4-1.3]

Country	Scenario	Household contacts < 5	Household contacts 5-14	Household contacts 15+	People living with HIV/AIDS
Pakistan	No TPT	530.9 [289.4-835.3]	882.3 [349.4-1731.5]	1180.9 [696.2-1813.9]	20.5 [10.2-33.5]
	3HP	468.1 [248.0-752.8]	809.6 [298.0-1648.4]	993.8 [601.0-1493.9]	15.9 [7.9-26.3]
	Cases averted by 3HP	62.8 [25.1-115.3]	72.7 [26.4-142.4]	187.1 [68.0-366.6]	4.6 [2.3-7.5]
Rwanda	No TPT	7.0 [3.8-10.9]	10.9 [4.3-21.4]	12.8 [7.5-19.6]	2.0 [0.9-3.6]
	3HP	6.1 [3.2-9.9]	10.0 [3.7-20.4]	10.7 [6.5-16.1]	1.6 [0.7-2.9]
	Cases averted by 3HP	0.8 [0.3-1.5]	0.9 [0.3-1.8]	2.0 [0.7-4.0]	0.4 [0.2-0.8]
Somalia	No TPT	35.1 [19.1-55.3]	44.4 [17.5-87.4]	55.5 [32.7-85.2]	0.2 [0.1-0.3]
	3HP	30.8 [16.3-49.7]	40.7 [14.9-83.2]	46.6 [28.2-70.1]	0.2 [0.1-0.3]
	Cases averted by 3HP	4.3 [1.7-7.9]	3.7 [1.3-7.3]	8.9 [3.2-17.4]	0.0 [0.0-0.1]
South Africa	No TPT	117.9 [64.3-185.5]	198.4 [78.7-388.9]	392.6 [231.4-603.3]	217.6 [104.8-367.4]
	3HP	103.9 [55.0-167.0]	182.3 [67.3-370.6]	331.0 [200.1-497.6]	171.7 [82.1-292.6]
	Cases averted by 3HP	14.0 [5.6-25.7]	16.0 [5.9-31.4]	61.6 [22.4-120.6]	45.8 [20.1-81.4]
Tajikistan	No TPT	7.1 [3.9-11.2]	13.6 [5.4-26.7]	19.2 [11.3-29.4]	0.3 [0.1-0.5]
	3HP	6.3 [3.3-10.1]	12.5 [4.6-25.5]	16.1 [9.7-24.2]	0.2 [0.1-0.4]
	Cases averted by 3HP	0.8 [0.3-1.6]	1.1 [0.4-2.2]	3.1 [1.1-6.0]	0.1 [0.0-0.1]
Tanzania	No TPT	141.7 [77.0-223.1]	189.3 [74.7-371.9]	205.0 [120.8-314.6]	55.5 [27.0-92.5]
	3HP	124.3 [65.6-200.4]	173.5 [63.6-354.2]	172.0 [104.0-258.6]	43.8 [21.2-73.9]
	Cases averted by 3HP	17.3 [6.9-31.8]	15.7 [5.7-30.8]	32.9 [11.9-64.5]	11.7 [5.2-20.5]
Thailand	No TPT	36.7 [20.0-57.7]	64.6 [25.7-126.5]	177.9 [104.8-273.4]	20.1 [9.1-35.9]
	3HP	32.4 [17.2-52.1]	59.4 [22.0-120.6]	150.4 [90.8-226.2]	15.9 [7.1-28.9]
	Cases averted by 3HP	4.3 [1.7-7.9]	5.1 [1.9-10.1]	27.5 [10.0-53.8]	4.2 [1.6-8.1]
Timor-Leste	No TPT	5.6 [3.1-8.9]	8.7 [3.4-17.0]	10.9 [6.4-16.8]	0.2 [0.1-0.3]
	3HP	5.0 [2.6-8.0]	7.9 [2.9-16.2]	9.2 [5.6-13.8]	0.2 [0.1-0.3]
	Cases averted by 3HP	0.7 [0.3-1.2]	0.7 [0.3-1.4]	1.7 [0.6-3.4]	0.0 [0.0-0.1]
Uganda	No TPT	100.7 [54.8-158.6]	153.4 [60.7-301.1]	150.6 [88.8-231.1]	33.0 [14.8-58.9]
	3HP	88.6 [46.9-142.6]	140.7 [51.7-286.8]	126.3 [76.4-189.8]	26.1 [11.6-47.5]
	Cases averted by 3HP	12.2 [4.9-22.3]	12.6 [4.6-24.8]	24.3 [8.8-47.6]	6.9 [2.6-13.1]
Zambia	No TPT	71.5 [38.9-112.6]	93.6 [37.0-184.0]	91.8 [54.1-140.9]	82.8 [39.7-141.0]
	3HP	62.8 [33.1-101.2]	85.9 [31.5-175.3]	77.1 [46.6-115.8]	65.2 [30.9-112.3]
	Cases averted by 3HP	8.8 [3.5-16.1]	7.7 [2.8-15.2]	14.8 [5.3-28.9]	17.6 [7.7-31.6]
Zimbabwe	No TPT	21.2 [11.5-33.3]	34.2 [13.5-67.0]	43.6 [25.7-67.0]	15.1 [7.1-26.1]
	3HP	18.6 [9.8-29.9]	31.4 [11.6-63.8]	36.6 [22.2-55.1]	12.0 [5.6-20.9]
	Cases averted by 3HP	2.6 [1.0-4.7]	2.7 [1.0-5.3]	7.0 [2.5-13.7]	3.1 [1.3-5.7]

Appendix Table 12: Projected TB cases per 100 contacts and per 100 people living with HIV/AIDS, with and without 3HP

Country	Scenario	Household contacts < 5	Household contacts 5-14	Household contacts 15+	People living with HIV/AIDS
Bangladesh	No TPT	16.3 [8.9-25.7]	12.8 [5.1-25.0]	6.5 [3.8-10.0]	4.2 [1.9-7.5]
	3HP	14.4 [7.7-23.2]	11.8 [4.3-23.9]	5.5 [3.3-8.3]	3.3 [1.5-6.0]
	Cases averted by 3HP	1.9 [0.8-3.5]	1.0 [0.4-2.0]	1.0 [0.4-2.0]	0.9 [0.3-1.7]
Brazil	No TPT	16.3 [8.9-25.6]	12.8 [5.1-25.1]	6.5 [3.9-10.1]	2.1 [0.9-3.8]
	3HP	14.4 [7.7-23.2]	11.8 [4.3-23.9]	5.5 [3.3-8.3]	1.7 [0.7-3.1]
	Cases averted by 3HP	1.9 [0.8-3.5]	1.0 [0.4-2.0]	1.0 [0.4-2.0]	0.5 [0.2-0.9]
Burundi	No TPT	16.2 [8.8-25.5]	12.7 [5.0-25.0]	6.5 [3.8-9.9]	3.3 [1.5-5.8]
	3HP	14.2 [7.5-22.9]	11.7 [4.3-23.8]	5.4 [3.3-8.1]	2.6 [1.2-4.7]
	Cases averted by 3HP	2.0 [0.8-3.6]	1.1 [0.4-2.1]	1.0 [0.4-2.0]	0.7 [0.3-1.3]
Cambodia	No TPT	16.3 [8.9-25.6]	12.8 [5.1-25.0]	6.5 [3.8-10.0]	8.6 [4.0-15.0]
	3HP	14.3 [7.6-23.1]	11.7 [4.3-23.9]	5.5 [3.3-8.3]	6.7 [3.1-12.0]
	Cases averted by 3HP	1.9 [0.8-3.5]	1.0 [0.4-2.0]	1.0 [0.4-2.0]	1.8 [0.7-3.4]
Congo DRC	No TPT	16.2 [8.8-25.5]	12.7 [5.0-25.0]	6.5 [3.8-9.9]	4.4 [2.1-7.6]
	3HP	14.2 [7.5-22.9]	11.6 [4.3-23.8]	5.4 [3.3-8.1]	3.5 [1.6-6.1]
	Cases averted by 3HP	2.0 [0.8-3.6]	1.1 [0.4-2.1]	1.0 [0.4-2.0]	0.9 [0.4-1.7]
Eswatini	No TPT	16.2 [8.8-25.5]	12.8 [5.1-25.0]	6.5 [3.8-10.0]	4.9 [2.3-8.3]
	3HP	14.3 [7.5-22.9]	11.7 [4.3-23.9]	5.5 [3.3-8.2]	3.9 [1.8-6.7]
	Cases averted by 3HP	1.9 [0.8-3.6]	1.0 [0.4-2.0]	1.0 [0.4-2.0]	1.0 [0.4-1.8]
Ethiopia	No TPT	16.2 [8.8-25.6]	12.7 [5.0-25.0]	6.5 [3.8-10.0]	3.7 [1.8-6.4]
	3HP	14.3 [7.6-23.0]	11.7 [4.3-23.8]	5.4 [3.3-8.2]	3.0 [1.4-5.2]
	Cases averted by 3HP	1.9 [0.8-3.6]	1.1 [0.4-2.1]	1.0 [0.4-2.0]	0.8 [0.3-1.4]
Ghana	No TPT	16.2 [8.8-25.5]	12.7 [5.0-25.0]	6.5 [3.8-10.0]	4.4 [2.1-7.6]
	3HP	14.3 [7.5-22.9]	11.7 [4.3-23.8]	5.5 [3.3-8.2]	3.5 [1.6-6.0]
	Cases averted by 3HP	2.0 [0.8-3.6]	1.0 [0.4-2.1]	1.0 [0.4-2.0]	0.9 [0.4-1.7]
Haiti	No TPT	16.3 [8.9-25.6]	12.8 [5.1-25.0]	6.5 [3.8-10.0]	4.5 [2.2-7.6]
	3HP	14.3 [7.6-23.1]	11.7 [4.3-23.8]	5.5 [3.3-8.3]	3.6 [1.7-6.1]
	Cases averted by 3HP	1.9 [0.8-3.5]	1.0 [0.4-2.0]	1.0 [0.4-2.0]	0.9 [0.4-1.6]
India	No TPT	16.3 [8.9-25.6]	12.8 [5.1-25.0]	6.5 [3.8-10.0]	4.4 [2.1-7.4]
	3HP	14.3 [7.6-23.1]	11.7 [4.3-23.9]	5.5 [3.3-8.3]	3.4 [1.6-5.9]
	Cases averted by 3HP	1.9 [0.8-3.5]	1.0 [0.4-2.0]	1.0 [0.4-2.0]	0.9 [0.4-1.6]
Indonesia	No TPT	16.3 [8.9-25.6]	12.8 [5.1-25.0]	6.5 [3.8-10.0]	8.3 [4.1-13.5]
	3HP	14.3 [7.6-23.0]	11.7 [4.3-23.9]	5.5 [3.3-8.3]	6.5 [3.2-10.8]
	Cases averted by 3HP	1.9 [0.8-3.6]	1.0 [0.4-2.0]	1.0 [0.4-2.0]	1.8 [0.8-3.0]
Kenya	No TPT	16.2 [8.8-25.5]	12.7 [5.0-25.0]	6.5 [3.8-10.0]	2.0 [0.9-3.4]
	3HP	14.2 [7.5-22.9]	11.7 [4.3-23.8]	5.5 [3.3-8.2]	1.6 [0.7-2.7]
	Cases averted by 3HP	2.0 [0.8-3.6]	1.0 [0.4-2.0]	1.0 [0.4-2.0]	0.4 [0.2-0.8]
Lesotho	No TPT	16.3 [8.9-25.6]	12.8 [5.1-25.0]	6.5 [3.8-10.0]	5.4 [2.6-9.3]
	3HP	14.3 [7.6-23.1]	11.7 [4.3-23.8]	5.5 [3.3-8.2]	4.3 [2.0-7.5]
	Cases averted by 3HP	1.9 [0.8-3.5]	1.0 [0.4-2.0]	1.0 [0.4-2.0]	1.1 [0.5-2.1]
Liberia	No TPT	16.2 [8.8-25.5]	12.7 [5.0-25.0]	6.5 [3.8-10.0]	3.8 [1.8-6.5]
	3HP	14.2 [7.5-22.9]	11.7 [4.3-23.8]	5.5 [3.3-8.2]	3.0 [1.4-5.2]
	Cases averted by 3HP	2.0 [0.8-3.6]	1.0 [0.4-2.1]	1.0 [0.4-2.0]	0.8 [0.4-1.4]
Malawi	No TPT	16.2 [8.8-25.5]	12.7 [5.0-25.0]	6.5 [3.8-9.9]	3.4 [1.6-5.6]
	3HP	14.2 [7.5-22.9]	11.7 [4.3-23.8]	5.4 [3.3-8.2]	2.7 [1.3-4.5]
	Cases averted by 3HP	2.0 [0.8-3.6]	1.0 [0.4-2.1]	1.0 [0.4-2.0]	0.7 [0.3-1.2]
Mongolia	No TPT	16.3 [8.9-25.6]	12.8 [5.1-25.1]	6.5 [3.8-10.0]	6.6 [3.1-11.1]
	3HP	14.4 [7.6-23.1]	11.8 [4.3-23.9]	5.5 [3.3-8.2]	5.2 [2.5-8.9]
	Cases averted by 3HP	1.9 [0.8-3.5]	1.0 [0.4-2.0]	1.0 [0.4-2.0]	1.2 [0.5-2.3]
Mozambique	No TPT	16.2 [8.8-25.5]	12.7 [5.0-25.0]	6.5 [3.8-9.9]	6.1 [3.0-10.0]
	3HP	14.2 [7.5-22.9]	11.6 [4.3-23.8]	5.4 [3.3-8.2]	4.8 [2.3-8.0]
	Cases averted by 3HP	2.0 [0.8-3.6]	1.1 [0.4-2.1]	1.0 [0.4-2.0]	1.3 [0.6-2.2]
Namibia	No TPT	16.2 [8.8-25.5]	12.8 [5.1-25.0]	6.5 [3.8-10.0]	6.8 [3.4-11.1]
	3HP	14.3 [7.6-23.0]	11.7 [4.3-23.8]	5.5 [3.3-8.2]	5.4 [2.7-8.9]
	Cases averted by 3HP	1.9 [0.8-3.6]	1.0 [0.4-2.0]	1.0 [0.4-2.0]	1.4 [0.7-2.4]
Pakistan	No TPT	16.3 [8.9-25.6]	12.7 [5.0-25.0]	6.5 [3.8-10.0]	4.8 [2.4-7.8]
	3HP	14.3 [7.6-23.1]	11.7 [4.3-23.8]	5.5 [3.3-8.2]	3.7 [1.8-6.1]
	Cases averted by 3HP	1.9 [0.8-3.5]	1.1 [0.4-2.1]	1.0 [0.4-2.0]	1.1 [0.5-1.8]
Rwanda	No TPT	16.2 [8.8-25.5]	12.7 [5.0-25.0]	6.5 [3.8-10.0]	1.0 [0.4-1.7]

Country	Scenario	Household contacts < 5	Household contacts 5-14	Household contacts 15+	People living with HIV/AIDS
	3HP	14.3 [7.6-23.0]	11.7 [4.3-23.8]	5.5 [3.3-8.2]	0.8 [0.3-1.4]
	Cases averted by 3HP	1.9 [0.8-3.6]	1.1 [0.4-2.1]	1.0 [0.4-2.0]	0.2 [0.1-0.4]
Somalia	No TPT	16.1 [8.8-25.4]	12.7 [5.0-24.9]	6.5 [3.8-9.9]	3.7 [1.8-6.1]
	3HP	14.2 [7.5-22.8]	11.6 [4.2-23.8]	5.4 [3.3-8.2]	2.9 [1.4-4.8]
	Cases averted by 3HP	2.0 [0.8-3.6]	1.1 [0.4-2.1]	1.0 [0.4-2.0]	0.8 [0.4-1.4]
South Africa	No TPT	16.2 [8.9-25.6]	12.8 [5.1-25.0]	6.5 [3.8-10.0]	5.2 [2.5-8.7]
	3HP	14.3 [7.6-23.0]	11.7 [4.3-23.9]	5.5 [3.3-8.3]	4.1 [1.9-6.9]
	Cases averted by 3HP	1.9 [0.8-3.5]	1.0 [0.4-2.0]	1.0 [0.4-2.0]	1.1 [0.5-1.9]
Tajikistan	No TPT	16.2 [8.8-25.5]	12.8 [5.1-25.0]	6.5 [3.8-10.0]	3.3 [1.6-5.5]
	3HP	14.3 [7.5-22.9]	11.7 [4.3-23.8]	5.5 [3.3-8.2]	2.6 [1.2-4.4]
	Cases averted by 3HP	1.9 [0.8-3.5]	1.1 [0.4-2.1]	1.0 [0.4-2.0]	0.7 [0.3-1.2]
Tanzania	No TPT	16.2 [8.8-25.4]	12.7 [5.0-25.0]	6.5 [3.8-9.9]	4.9 [2.4-8.2]
	3HP	14.2 [7.5-22.9]	11.6 [4.3-23.8]	5.4 [3.3-8.2]	3.9 [1.9-6.5]
	Cases averted by 3HP	2.0 [0.8-3.6]	1.1 [0.4-2.1]	1.0 [0.4-2.0]	1.0 [0.5-1.8]
Thailand	No TPT	16.3 [8.9-25.6]	12.8 [5.1-25.1]	6.6 [3.9-10.1]	5.0 [2.3-8.9]
	3HP	14.4 [7.6-23.1]	11.8 [4.4-23.9]	5.5 [3.3-8.3]	3.9 [1.8-7.2]
	Cases averted by 3HP	1.9 [0.8-3.5]	1.0 [0.4-2.0]	1.0 [0.4-2.0]	1.1 [0.4-2.0]
Timor-Leste	No TPT	16.3 [8.9-25.6]	12.7 [5.0-25.0]	6.5 [3.8-10.0]	8.0 [4.0-13.1]
	3HP	14.4 [7.6-23.1]	11.7 [4.3-23.8]	5.5 [3.3-8.2]	6.2 [3.1-10.3]
	Cases averted by 3HP	1.9 [0.8-3.5]	1.1 [0.4-2.1]	1.0 [0.4-2.0]	1.7 [0.8-2.9]
Uganda	No TPT	16.2 [8.8-25.5]	12.7 [5.0-25.0]	6.5 [3.8-9.9]	2.3 [1.0-4.0]
	3HP	14.3 [7.5-22.9]	11.7 [4.3-23.8]	5.4 [3.3-8.1]	1.8 [0.8-3.2]
	Cases averted by 3HP	2.0 [0.8-3.6]	1.0 [0.4-2.1]	1.0 [0.4-2.0]	0.5 [0.2-0.9]
Zambia	No TPT	16.2 [8.8-25.4]	12.7 [5.0-25.0]	6.5 [3.8-9.9]	5.0 [2.4-8.6]
	3HP	14.2 [7.5-22.9]	11.7 [4.3-23.8]	5.4 [3.3-8.2]	4.0 [1.9-6.8]
	Cases averted by 3HP	2.0 [0.8-3.6]	1.1 [0.4-2.1]	1.0 [0.4-2.0]	1.1 [0.5-1.9]
Zimbabwe	No TPT	16.2 [8.8-25.5]	12.8 [5.1-25.0]	6.5 [3.8-9.9]	3.4 [1.6-5.8]
	3HP	14.2 [7.5-22.9]	11.8 [4.3-23.9]	5.4 [3.3-8.2]	2.7 [1.2-4.6]
	Cases averted by 3HP	2.0 [0.8-3.6]	1.0 [0.4-2.0]	1.0 [0.4-2.0]	0.7 [0.3-1.3]

Appendix Table 13: Total projected TB deaths with and without 3HP

Country	Scenario	Household contacts < 5	Household contacts 5-14	Household contacts 15+	People living with HIV/AIDS
Bangladesh	No TPT	168.7 [88.9-271.0]	59.6 [15.5-138.1]	59.3 [19.1-117.7]	2.08 [0.81-4.12]
	3HP	104.9 [55.7-167.2]	39.3 [10.5-90.1]	37.6 [12.5-74.9]	1.63 [0.63-3.28]
	Deaths averted by 3HP	63.8 [32.3-105.5]	20.3 [4.9-48.3]	21.7 [6.4-43.2]	0.45 [0.14-0.94]
Brazil	No TPT	19.4 [9.8-32.1]	12.1 [3.4-27.3]	12.8 [4.3-25.3]	4.96 [1.93-9.94]
	3HP	13.2 [6.7-21.8]	8.7 [2.5-19.7]	9.0 [3.1-17.6]	3.92 [1.51-7.87]
	Deaths averted by 3HP	6.2 [3.1-10.4]	3.4 [0.9-7.7]	3.8 [1.1-7.7]	1.05 [0.38-2.18]
Burundi	No TPT	7.6 [3.7-12.5]	4.0 [1.1-9.1]	1.6 [0.5-3.2]	0.34 [0.13-0.67]
	3HP	4.6 [2.2-7.5]	2.6 [0.7-5.9]	1.0 [0.3-2.0]	0.27 [0.10-0.53]
	Deaths averted by 3HP	3.0 [1.4-5.1]	1.4 [0.4-3.3]	0.6 [0.2-1.2]	0.07 [0.02-0.16]
Cambodia	No TPT	9.1 [3.9-16.0]	2.4 [0.9-4.9]	8.8 [2.7-17.5]	0.66 [0.26-1.28]
	3HP	5.8 [2.5-10.3]	1.7 [0.6-3.5]	5.7 [1.8-11.5]	0.51 [0.20-1.01]
	Deaths averted by 3HP	3.3 [1.4-5.9]	0.7 [0.3-1.5]	3.1 [0.9-6.1]	0.14 [0.05-0.30]
Congo DRC	No TPT	179.2 [76.1-311.5]	61.8 [18.0-139.8]	35.0 [12.2-67.9]	2.76 [1.13-5.34]
	3HP	111.1 [48.0-192.3]	41.0 [12.2-92.3]	21.9 [7.9-42.9]	2.18 [0.88-4.24]
	Deaths averted by 3HP	68.1 [27.8-121.6]	20.8 [5.7-48.1]	13.1 [4.2-25.7]	0.58 [0.21-1.19]
Eswatini	No TPT	1.8 [0.8-3.0]	1.2 [0.3-2.7]	0.8 [0.2-1.6]	0.69 [0.28-1.32]
	3HP	1.1 [0.5-1.9]	0.8 [0.2-1.8]	0.5 [0.2-1.0]	0.54 [0.22-1.05]
	Deaths averted by 3HP	0.7 [0.3-1.2]	0.4 [0.1-0.9]	0.3 [0.1-0.6]	0.15 [0.05-0.30]
Ethiopia	No TPT	61.5 [26.1-108.7]	17.9 [6.2-38.7]	14.8 [6.1-27.6]	1.77 [0.70-3.52]
	3HP	39.0 [16.6-69.1]	12.2 [4.2-26.5]	9.7 [4.0-18.3]	1.39 [0.54-2.79]
	Deaths averted by 3HP	22.5 [9.2-40.7]	5.7 [1.9-12.5]	5.1 [2.0-9.6]	0.38 [0.14-0.79]
Ghana	No TPT	11.0 [5.8-17.6]	8.8 [3.0-18.5]	6.3 [2.0-11.9]	2.37 [0.96-4.57]
	3HP	7.2 [3.8-11.5]	6.2 [2.1-13.1]	4.1 [1.3-7.9]	1.86 [0.75-3.61]
	Deaths averted by 3HP	3.8 [2.0-6.3]	2.6 [0.9-5.5]	2.2 [0.7-4.1]	0.51 [0.19-1.03]
Haiti	No TPT	5.4 [2.3-9.3]	1.1 [0.4-2.3]	2.9 [0.9-5.8]	0.27 [0.11-0.52]
	3HP	3.5 [1.5-6.0]	0.8 [0.3-1.7]	1.9 [0.6-3.8]	0.21 [0.09-0.41]
	Deaths averted by 3HP	1.9 [0.8-3.3]	0.3 [0.1-0.7]	1.0 [0.3-2.0]	0.06 [0.02-0.11]
India	No TPT	1101.5 [582.1-1772.9]	510.8 [139.5-1162.8]	624.6 [272.5-1146.1]	6.70 [2.76-12.82]
	3HP	700.8 [371.4-1121.9]	347.9 [96.3-786.4]	410.7 [179.5-757.7]	5.26 [2.14-10.07]
	Deaths averted by 3HP	400.7 [205.3-661.5]	163.0 [43.0-376.0]	213.9 [91.4-392.5]	1.44 [0.54-2.89]
Indonesia	No TPT	159.2 [80.5-263.6]	116.3 [39.7-246.8]	241.3 [131.7-393.7]	3.36 [1.41-6.27]
	3HP	103.6 [52.6-171.0]	80.9 [27.8-171.5]	158.6 [85.3-261.5]	2.63 [1.10-4.95]
	Deaths averted by 3HP	55.6 [27.4-94.4]	35.4 [11.7-76.6]	82.7 [45.5-133.6]	0.72 [0.29-1.40]
Kenya	No TPT	44.8 [19.5-77.2]	28.3 [7.7-63.8]	18.9 [5.8-37.2]	2.49 [1.01-4.83]
	3HP	28.0 [12.4-48.1]	19.1 [5.3-43.0]	12.1 [3.8-24.1]	1.95 [0.78-3.81]
	Deaths averted by 3HP	16.7 [7.1-29.7]	9.2 [2.4-21.0]	6.7 [1.9-13.2]	0.54 [0.19-1.10]
Lesotho	No TPT	3.7 [1.9-6.1]	3.1 [1.0-6.7]	2.5 [0.8-4.7]	1.87 [0.76-3.61]
	3HP	2.4 [1.2-3.9]	2.2 [0.7-4.6]	1.7 [0.6-3.2]	1.47 [0.59-2.86]
	Deaths averted by 3HP	1.3 [0.6-2.2]	1.0 [0.3-2.1]	0.8 [0.3-1.5]	0.40 [0.14-0.81]
Liberia	No TPT	5.9 [2.5-10.3]	3.9 [1.0-8.9]	3.4 [0.9-6.6]	0.15 [0.06-0.29]
	3HP	3.7 [1.6-6.5]	2.6 [0.7-6.0]	2.2 [0.6-4.3]	0.12 [0.05-0.23]
	Deaths averted by 3HP	2.2 [0.9-4.0]	1.3 [0.3-3.0]	1.2 [0.3-2.3]	0.03 [0.01-0.06]
Malawi	No TPT	14.5 [6.4-24.8]	6.8 [1.8-15.4]	3.8 [1.2-7.4]	1.07 [0.44-2.04]
	3HP	8.9 [4.0-15.3]	4.5 [1.2-10.2]	2.4 [0.8-4.8]	0.84 [0.35-1.61]
	Deaths averted by 3HP	5.6 [2.4-9.8]	2.3 [0.6-5.3]	1.4 [0.4-2.7]	0.23 [0.09-0.46]
Mongolia	No TPT	1.9 [0.9-3.2]	1.1 [0.3-2.4]	2.6 [0.9-4.7]	0.00 [0.00-0.01]
	3HP	1.2 [0.5-1.9]	0.7 [0.2-1.6]	1.7 [0.6-3.1]	0.00 [0.00-0.01]
	Deaths averted by 3HP	0.7 [0.3-1.2]	0.4 [0.1-0.9]	0.9 [0.3-1.6]	0.00 [-0.00-0.00]
Mozambique	No TPT	63.6 [27.1-111.6]	18.6 [6.1-40.5]	8.1 [4.2-13.8]	9.87 [4.11-18.62]
	3HP	39.7 [17.0-70.0]	12.5 [4.2-27.2]	5.4 [2.7-9.5]	7.68 [3.17-14.58]
	Deaths averted by 3HP	23.8 [9.8-43.0]	6.1 [1.9-13.5]	2.6 [1.4-4.4]	2.19 [0.88-4.25]
Namibia	No TPT	2.9 [1.2-5.2]	1.8 [0.5-4.2]	2.1 [0.5-4.3]	0.52 [0.22-0.98]
	3HP	1.8 [0.8-3.2]	1.2 [0.3-2.8]	1.3 [0.3-2.8]	0.41 [0.17-0.77]
	Deaths averted by 3HP	1.1 [0.4-2.0]	0.6 [0.2-1.4]	0.7 [0.1-1.5]	0.11 [0.04-0.22]
Pakistan	No TPT	236.3 [100.2-414.3]	144.6 [38.1-334.7]	193.6 [64.5-373.6]	2.74 [1.15-5.12]
	3HP	150.9 [64.1-264.4]	97.2 [26.0-224.1]	125.2 [41.8-245.8]	2.10 [0.87-3.97]
	Deaths averted by 3HP	85.5 [34.8-153.6]	47.4 [11.9-112.2]	68.4 [22.5-130.3]	0.64 [0.27-1.19]
Rwanda	No TPT	2.4 [1.1-4.3]	2.4 [0.6-5.6]	0.8 [0.3-1.5]	0.30 [0.12-0.59]
	3HP	1.5 [0.7-2.6]	1.6 [0.4-3.6]	0.5 [0.2-1.0]	0.23 [0.09-0.47]

Country	Scenario	Household contacts < 5	Household contacts 5-14	Household contacts 15+	People living with HIV/AIDS
	Deaths averted by 3HP	0.9 [0.4-1.7]	0.8 [0.2-2.0]	0.3 [0.1-0.5]	0.06 [0.02-0.14]
Somalia	No TPT	18.4 [8.1-31.5]	7.8 [2.0-17.9]	10.2 [3.0-20.0]	0.03 [0.01-0.05]
	3HP	11.4 [5.1-19.5]	5.2 [1.4-11.9]	6.6 [1.9-13.1]	0.02 [0.01-0.04]
	Deaths averted by 3HP	7.0 [3.0-12.3]	2.6 [0.7-6.2]	3.7 [1.1-7.1]	0.01 [0.00-0.01]
South Africa	No TPT	48.8 [21.0-85.2]	41.1 [11.0-93.3]	38.2 [12.6-74.8]	31.07 [12.71-59.31]
	3HP	31.8 [13.8-55.3]	28.4 [7.7-64.4]	25.6 [8.6-50.2]	24.32 [9.86-46.69]
	Deaths averted by 3HP	17.1 [7.0-30.6]	12.6 [3.2-29.1]	12.6 [3.8-24.7]	6.75 [2.56-13.49]
Tajikistan	No TPT	3.9 [1.7-6.6]	2.0 [0.6-4.6]	2.8 [1.1-5.3]	0.04 [0.02-0.09]
	3HP	2.4 [1.1-4.0]	1.3 [0.4-3.0]	1.8 [0.7-3.4]	0.04 [0.01-0.07]
	Deaths averted by 3HP	1.5 [0.6-2.6]	0.7 [0.2-1.6]	1.0 [0.4-1.9]	0.01 [0.00-0.02]
Tanzania	No TPT	43.0 [19.2-75.7]	23.0 [6.5-52.5]	17.8 [5.7-35.2]	7.12 [2.93-13.54]
	3HP	26.2 [11.8-46.0]	14.9 [4.3-33.7]	11.1 [3.7-22.1]	5.55 [2.27-10.62]
	Deaths averted by 3HP	16.8 [7.2-30.4]	8.1 [2.1-19.1]	6.7 [2.0-13.3]	1.56 [0.60-3.11]
Thailand	No TPT	22.3 [10.3-37.5]	17.0 [4.7-37.8]	7.3 [3.6-12.7]	3.03 [1.18-6.03]
	3HP	14.1 [6.6-23.5]	11.6 [3.2-25.6]	5.0 [2.5-8.8]	2.37 [0.92-4.79]
	Deaths averted by 3HP	8.2 [3.7-14.1]	5.4 [1.4-12.4]	2.3 [1.1-4.0]	0.65 [0.21-1.38]
Timor-Leste	No TPT	2.3 [1.0-4.1]	1.5 [0.4-3.6]	1.5 [0.5-3.0]	0.03 [0.01-0.05]
	3HP	1.5 [0.6-2.7]	1.0 [0.3-2.4]	1.0 [0.3-2.0]	0.02 [0.01-0.04]
	Deaths averted by 3HP	0.8 [0.3-1.5]	0.5 [0.1-1.2]	0.5 [0.2-1.0]	0.01 [0.00-0.01]
Uganda	No TPT	42.5 [18.4-74.6]	19.5 [5.6-44.5]	21.7 [6.3-42.5]	4.67 [1.82-9.35]
	3HP	27.1 [11.8-47.3]	13.3 [3.9-30.1]	14.0 [4.2-27.7]	3.66 [1.41-7.41]
	Deaths averted by 3HP	15.4 [6.4-27.7]	6.2 [1.7-14.5]	7.7 [2.1-15.0]	1.01 [0.34-2.14]
Zambia	No TPT	38.2 [16.7-65.5]	21.4 [5.8-48.3]	6.4 [2.4-12.2]	10.81 [4.39-20.84]
	3HP	23.6 [10.5-40.3]	14.2 [3.9-31.9]	4.1 [1.6-7.8]	8.41 [3.39-16.33]
	Deaths averted by 3HP	14.6 [6.2-25.7]	7.2 [1.9-16.6]	2.3 [0.8-4.4]	2.40 [0.90-4.85]
Zimbabwe	No TPT	12.2 [5.6-20.7]	9.4 [2.6-20.9]	5.4 [1.6-10.7]	2.06 [0.83-4.01]
	3HP	7.4 [3.4-12.5]	6.3 [1.8-13.9]	3.5 [1.1-7.0]	1.62 [0.64-3.16]
	Deaths averted by 3HP	4.8 [2.1-8.3]	3.1 [0.8-7.0]	1.9 [0.5-3.8]	0.44 [0.16-0.91]

Appendix Table 14: Projected TB deaths per 100 contacts and per 100 people living with HIV/AIDS, with and without 3HP

Country	Scenario	Household contacts < 5	Household contacts 5-14	Household contacts 15+	People living with HIV/AIDS
Bangladesh	No TPT	12.5 [6.6-20.1]	2.0 [0.5-4.7]	0.6 [0.2-1.1]	0.66 [0.26-1.31]
	3HP	7.8 [4.1-12.4]	1.3 [0.4-3.1]	0.4 [0.1-0.7]	0.52 [0.20-1.04]
	Deaths averted by 3HP	4.7 [2.4-7.8]	0.7 [0.2-1.6]	0.2 [0.1-0.4]	0.14 [0.05-0.30]
Brazil	No TPT	9.3 [4.7-15.4]	2.5 [0.7-5.7]	0.5 [0.2-1.0]	0.41 [0.16-0.83]
	3HP	6.3 [3.2-10.5]	1.8 [0.5-4.1]	0.4 [0.1-0.7]	0.33 [0.13-0.66]
	Deaths averted by 3HP	3.0 [1.5-5.0]	0.7 [0.2-1.6]	0.2 [0.0-0.3]	0.09 [0.03-0.18]
Burundi	No TPT	10.5 [5.1-17.4]	3.0 [0.8-6.7]	0.6 [0.2-1.3]	0.44 [0.17-0.87]
	3HP	6.4 [3.1-10.5]	1.9 [0.5-4.3]	0.4 [0.1-0.8]	0.34 [0.13-0.69]
	Deaths averted by 3HP	4.2 [1.9-7.1]	1.0 [0.3-2.4]	0.2 [0.1-0.5]	0.10 [0.03-0.20]
Cambodia	No TPT	6.0 [2.6-10.6]	0.7 [0.3-1.5]	0.8 [0.2-1.5]	1.16 [0.46-2.27]
	3HP	3.8 [1.7-6.8]	0.5 [0.2-1.0]	0.5 [0.2-1.0]	0.90 [0.36-1.79]
	Deaths averted by 3HP	2.2 [0.9-3.9]	0.2 [0.1-0.4]	0.3 [0.1-0.5]	0.25 [0.09-0.52]
Congo DRC	No TPT	7.8 [3.3-13.5]	1.5 [0.4-3.4]	0.5 [0.2-1.0]	0.75 [0.31-1.46]
	3HP	4.8 [2.1-8.3]	1.0 [0.3-2.2]	0.3 [0.1-0.6]	0.59 [0.24-1.16]
	Deaths averted by 3HP	2.9 [1.2-5.3]	0.5 [0.1-1.2]	0.2 [0.1-0.4]	0.16 [0.06-0.32]
Eswatini	No TPT	8.7 [3.9-14.9]	3.0 [0.8-6.8]	0.8 [0.2-1.6]	0.66 [0.27-1.27]
	3HP	5.5 [2.5-9.3]	2.0 [0.6-4.6]	0.5 [0.2-1.1]	0.52 [0.21-1.00]
	Deaths averted by 3HP	3.3 [1.4-5.7]	1.0 [0.2-2.2]	0.3 [0.1-0.6]	0.14 [0.05-0.29]
Ethiopia	No TPT	6.2 [2.6-10.9]	0.9 [0.3-2.0]	0.4 [0.2-0.7]	0.55 [0.22-1.10]
	3HP	3.9 [1.7-6.9]	0.6 [0.2-1.4]	0.2 [0.1-0.5]	0.43 [0.17-0.87]
	Deaths averted by 3HP	2.2 [0.9-4.1]	0.3 [0.1-0.6]	0.1 [0.1-0.2]	0.12 [0.04-0.25]
Ghana	No TPT	12.6 [6.7-20.2]	5.3 [1.8-11.0]	1.5 [0.5-2.9]	0.69 [0.28-1.33]
	3HP	8.2 [4.4-13.1]	3.7 [1.3-7.8]	1.0 [0.3-1.9]	0.54 [0.22-1.05]
	Deaths averted by 3HP	4.4 [2.3-7.2]	1.5 [0.5-3.3]	0.5 [0.2-1.0]	0.15 [0.06-0.30]
Haiti	No TPT	7.8 [3.4-13.5]	0.8 [0.3-1.6]	0.6 [0.2-1.3]	0.66 [0.27-1.26]
	3HP	5.1 [2.3-8.8]	0.6 [0.2-1.2]	0.4 [0.1-0.8]	0.52 [0.21-0.99]
	Deaths averted by 3HP	2.7 [1.1-4.8]	0.2 [0.1-0.5]	0.2 [0.1-0.4]	0.14 [0.06-0.27]
India	No TPT	11.3 [6.0-18.2]	2.7 [0.7-6.2]	0.7 [0.3-1.3]	0.67 [0.28-1.28]
	3HP	7.2 [3.8-11.5]	1.9 [0.5-4.2]	0.5 [0.2-0.9]	0.53 [0.21-1.01]
	Deaths averted by 3HP	4.1 [2.1-6.8]	0.9 [0.2-2.0]	0.2 [0.1-0.5]	0.14 [0.05-0.29]
Indonesia	No TPT	8.0 [4.1-13.3]	2.7 [0.9-5.8]	1.3 [0.7-2.1]	1.27 [0.53-2.36]
	3HP	5.2 [2.7-8.6]	1.9 [0.7-4.0]	0.8 [0.5-1.4]	0.99 [0.41-1.87]
	Deaths averted by 3HP	2.8 [1.4-4.8]	0.8 [0.3-1.8]	0.4 [0.2-0.7]	0.27 [0.11-0.53]
Kenya	No TPT	8.3 [3.6-14.2]	3.0 [0.8-6.8]	0.7 [0.2-1.5]	0.28 [0.11-0.54]
	3HP	5.2 [2.3-8.9]	2.0 [0.6-4.6]	0.5 [0.2-1.0]	0.22 [0.09-0.43]
	Deaths averted by 3HP	3.1 [1.3-5.5]	1.0 [0.3-2.2]	0.3 [0.1-0.5]	0.06 [0.02-0.12]
Lesotho	No TPT	11.3 [5.6-18.5]	4.6 [1.4-9.7]	1.5 [0.5-2.8]	0.81 [0.33-1.56]
	3HP	7.3 [3.7-11.9]	3.2 [1.0-6.7]	1.0 [0.3-1.9]	0.63 [0.25-1.24]
	Deaths averted by 3HP	4.0 [1.9-6.7]	1.4 [0.4-3.0]	0.5 [0.2-0.9]	0.17 [0.06-0.35]
Liberia	No TPT	7.2 [3.1-12.6]	2.7 [0.7-6.2]	1.1 [0.3-2.1]	0.62 [0.25-1.18]
	3HP	4.5 [1.9-7.9]	1.8 [0.5-4.1]	0.7 [0.2-1.4]	0.49 [0.20-0.94]
	Deaths averted by 3HP	2.7 [1.1-4.8]	0.9 [0.2-2.1]	0.4 [0.1-0.7]	0.13 [0.05-0.26]
Malawi	No TPT	8.6 [3.8-14.7]	2.4 [0.6-5.5]	0.6 [0.2-1.3]	0.44 [0.18-0.85]
	3HP	5.3 [2.4-9.0]	1.6 [0.4-3.6]	0.4 [0.1-0.8]	0.35 [0.14-0.67]
	Deaths averted by 3HP	3.3 [1.4-5.8]	0.8 [0.2-1.9]	0.2 [0.1-0.5]	0.10 [0.04-0.19]
Mongolia	No TPT	9.5 [4.4-16.1]	1.9 [0.5-4.4]	1.7 [0.6-3.2]	1.02 [0.37-2.24]
	3HP	5.9 [2.7-9.9]	1.3 [0.3-2.9]	1.1 [0.4-2.1]	0.80 [0.29-1.79]
	Deaths averted by 3HP	3.6 [1.6-6.3]	0.6 [0.2-1.5]	0.6 [0.2-1.1]	0.07 [-0.00-0.26]
Mozambique	No TPT	6.6 [2.8-11.6]	1.1 [0.4-2.4]	0.3 [0.1-0.4]	0.78 [0.33-1.47]
	3HP	4.1 [1.8-7.3]	0.7 [0.2-1.6]	0.2 [0.1-0.3]	0.61 [0.25-1.15]
	Deaths averted by 3HP	2.5 [1.0-4.5]	0.4 [0.1-0.8]	0.1 [0.0-0.1]	0.17 [0.07-0.34]
Namibia	No TPT	6.3 [2.7-11.1]	1.9 [0.5-4.3]	0.8 [0.2-1.6]	0.92 [0.39-1.73]
	3HP	3.9 [1.7-6.9]	1.2 [0.3-2.8]	0.5 [0.1-1.0]	0.72 [0.30-1.36]
	Deaths averted by 3HP	2.4 [1.0-4.3]	0.6 [0.2-1.5]	0.3 [0.1-0.6]	0.20 [0.08-0.38]
Pakistan	No TPT	7.2 [3.1-12.7]	2.1 [0.6-4.8]	1.1 [0.4-2.1]	0.64 [0.27-1.20]
	3HP	4.6 [2.0-8.1]	1.4 [0.4-3.2]	0.7 [0.2-1.4]	0.49 [0.20-0.93]
	Deaths averted by 3HP	2.6 [1.1-4.7]	0.7 [0.2-1.6]	0.4 [0.1-0.7]	0.15 [0.06-0.28]
Rwanda	No TPT	5.6 [2.5-10.0]	2.9 [0.8-6.5]	0.4 [0.2-0.8]	0.14 [0.05-0.28]

Country	Scenario	Household contacts < 5	Household contacts 5-14	Household contacts 15+	People living with HIV/AIDS
	3HP	3.5 [1.5-6.2]	1.9 [0.5-4.2]	0.3 [0.1-0.5]	0.11 [0.04-0.22]
	Deaths averted by 3HP	2.1 [0.9-3.9]	1.0 [0.2-2.3]	0.1 [0.1-0.3]	0.03 [0.01-0.06]
Somalia	No TPT	8.5 [3.7-14.5]	2.2 [0.6-5.1]	1.2 [0.3-2.3]	0.51 [0.21-0.97]
	3HP	5.3 [2.3-9.0]	1.5 [0.4-3.4]	0.8 [0.2-1.5]	0.40 [0.16-0.76]
	Deaths averted by 3HP	3.2 [1.4-5.7]	0.8 [0.2-1.8]	0.4 [0.1-0.8]	0.11 [0.04-0.21]
South Africa	No TPT	6.7 [2.9-11.7]	2.6 [0.7-6.0]	0.6 [0.2-1.2]	0.74 [0.30-1.41]
	3HP	4.4 [1.9-7.6]	1.8 [0.5-4.1]	0.4 [0.1-0.8]	0.58 [0.23-1.11]
	Deaths averted by 3HP	2.4 [1.0-4.2]	0.8 [0.2-1.9]	0.2 [0.1-0.4]	0.16 [0.06-0.32]
Tajikistan	No TPT	8.8 [3.9-15.0]	1.9 [0.5-4.3]	1.0 [0.4-1.8]	0.49 [0.20-0.95]
	3HP	5.4 [2.4-9.2]	1.2 [0.3-2.8]	0.6 [0.2-1.1]	0.39 [0.16-0.75]
	Deaths averted by 3HP	3.4 [1.4-6.0]	0.6 [0.2-1.5]	0.4 [0.1-0.7]	0.10 [0.03-0.21]
Tanzania	No TPT	4.9 [2.2-8.6]	1.5 [0.4-3.5]	0.6 [0.2-1.1]	0.63 [0.26-1.19]
	3HP	3.0 [1.3-5.2]	1.0 [0.3-2.3]	0.4 [0.1-0.7]	0.49 [0.20-0.94]
	Deaths averted by 3HP	1.9 [0.8-3.5]	0.5 [0.1-1.3]	0.2 [0.1-0.4]	0.14 [0.05-0.27]
Thailand	No TPT	9.9 [4.6-16.6]	3.4 [0.9-7.5]	0.3 [0.1-0.5]	0.75 [0.29-1.50]
	3HP	6.3 [2.9-10.4]	2.3 [0.6-5.1]	0.2 [0.1-0.3]	0.59 [0.23-1.19]
	Deaths averted by 3HP	3.6 [1.6-6.3]	1.1 [0.3-2.5]	0.1 [0.0-0.1]	0.16 [0.05-0.34]
Timor-Leste	No TPT	6.8 [2.9-11.9]	2.3 [0.6-5.2]	0.9 [0.3-1.8]	1.02 [0.42-1.93]
	3HP	4.3 [1.9-7.7]	1.5 [0.4-3.5]	0.6 [0.2-1.2]	0.79 [0.32-1.50]
	Deaths averted by 3HP	2.4 [1.0-4.4]	0.8 [0.2-1.8]	0.3 [0.1-0.6]	0.21 [0.08-0.44]
Uganda	No TPT	6.8 [3.0-12.0]	1.6 [0.5-3.7]	0.9 [0.3-1.8]	0.32 [0.12-0.64]
	3HP	4.4 [1.9-7.6]	1.1 [0.3-2.5]	0.6 [0.2-1.2]	0.25 [0.10-0.51]
	Deaths averted by 3HP	2.5 [1.0-4.5]	0.5 [0.1-1.2]	0.3 [0.1-0.6]	0.07 [0.02-0.15]
Zambia	No TPT	8.6 [3.8-14.8]	2.9 [0.8-6.6]	0.4 [0.2-0.9]	0.66 [0.27-1.27]
	3HP	5.3 [2.4-9.1]	1.9 [0.5-4.3]	0.3 [0.1-0.5]	0.51 [0.21-0.99]
	Deaths averted by 3HP	3.3 [1.4-5.8]	1.0 [0.3-2.2]	0.2 [0.1-0.3]	0.15 [0.05-0.29]
Zimbabwe	No TPT	9.3 [4.3-15.8]	3.5 [1.0-7.8]	0.8 [0.2-1.6]	0.46 [0.18-0.89]
	3HP	5.7 [2.6-9.5]	2.4 [0.7-5.2]	0.5 [0.2-1.0]	0.36 [0.14-0.70]
	Deaths averted by 3HP	3.7 [1.6-6.4]	1.1 [0.3-2.6]	0.3 [0.1-0.6]	0.10 [0.04-0.20]

Appendix Figure 4A: Comparative cost-effectiveness of TPT for different target populations

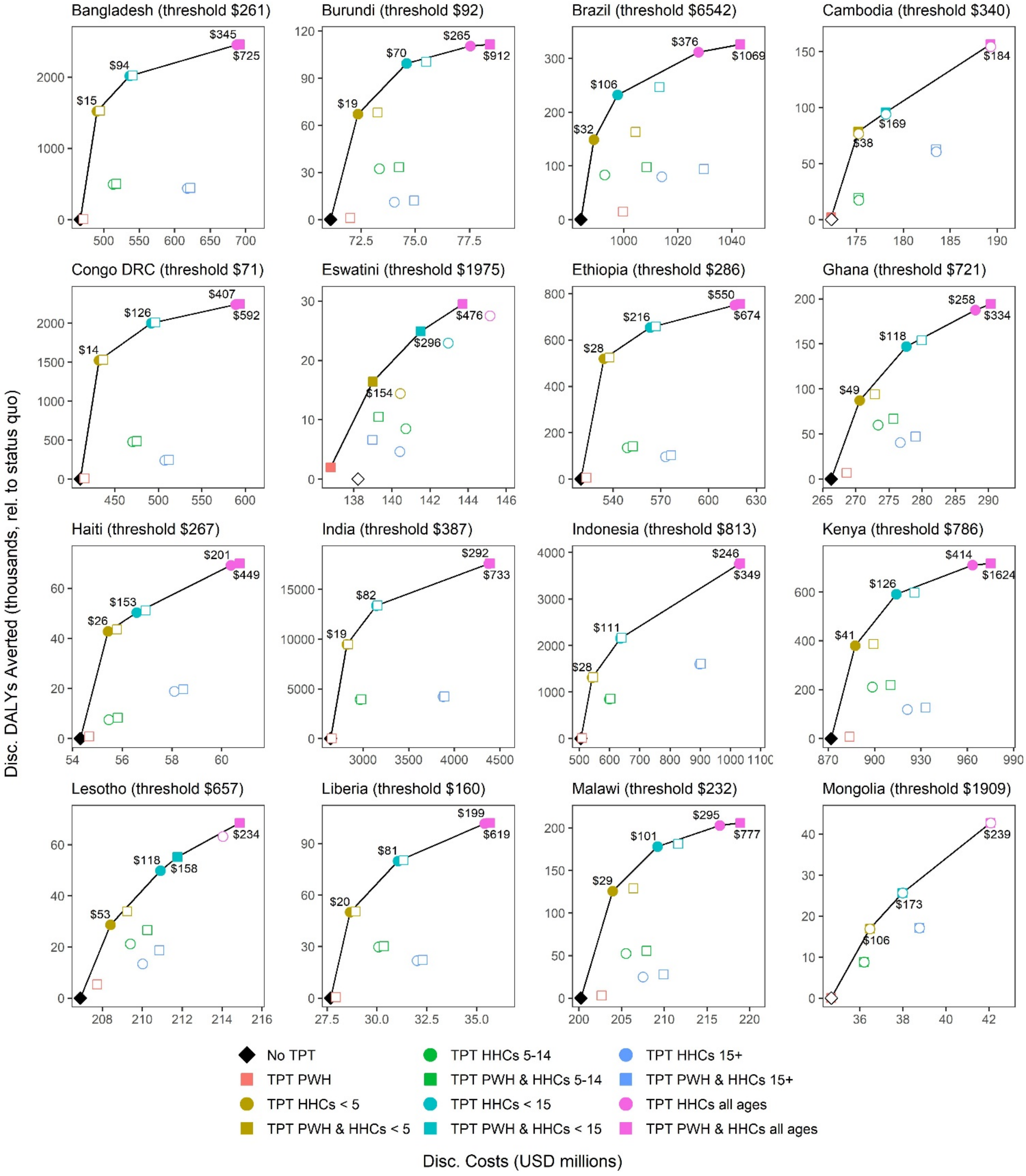

Figure shows incremental cost-effectiveness ratios of all combinations of strategies: 2 TPT scenarios (TPT or no TPT) and 4 target populations (contacts <5 years, contacts 5-14 years, contacts 15+ years and people with HIV: PWH). The incremental cost-effectiveness ratios are plotted on cost-effectiveness frontiers depicting discounted costs in 2020 USD on the x-axis and discounted disability adjusted life years averted relative to the No TPT scenario on the y-axis. Strategies appearing on the frontier are shaded, while dominated and extended dominated strategies

are unshaded (white fill). Shapes indicate whether TPT for PWH is included in the strategy, and colors indicate different contact age combinations. GNI p.c. = Gross National Income per capita.

Appendix Figure 4B: Comparative cost-effectiveness of TPT for different target populations

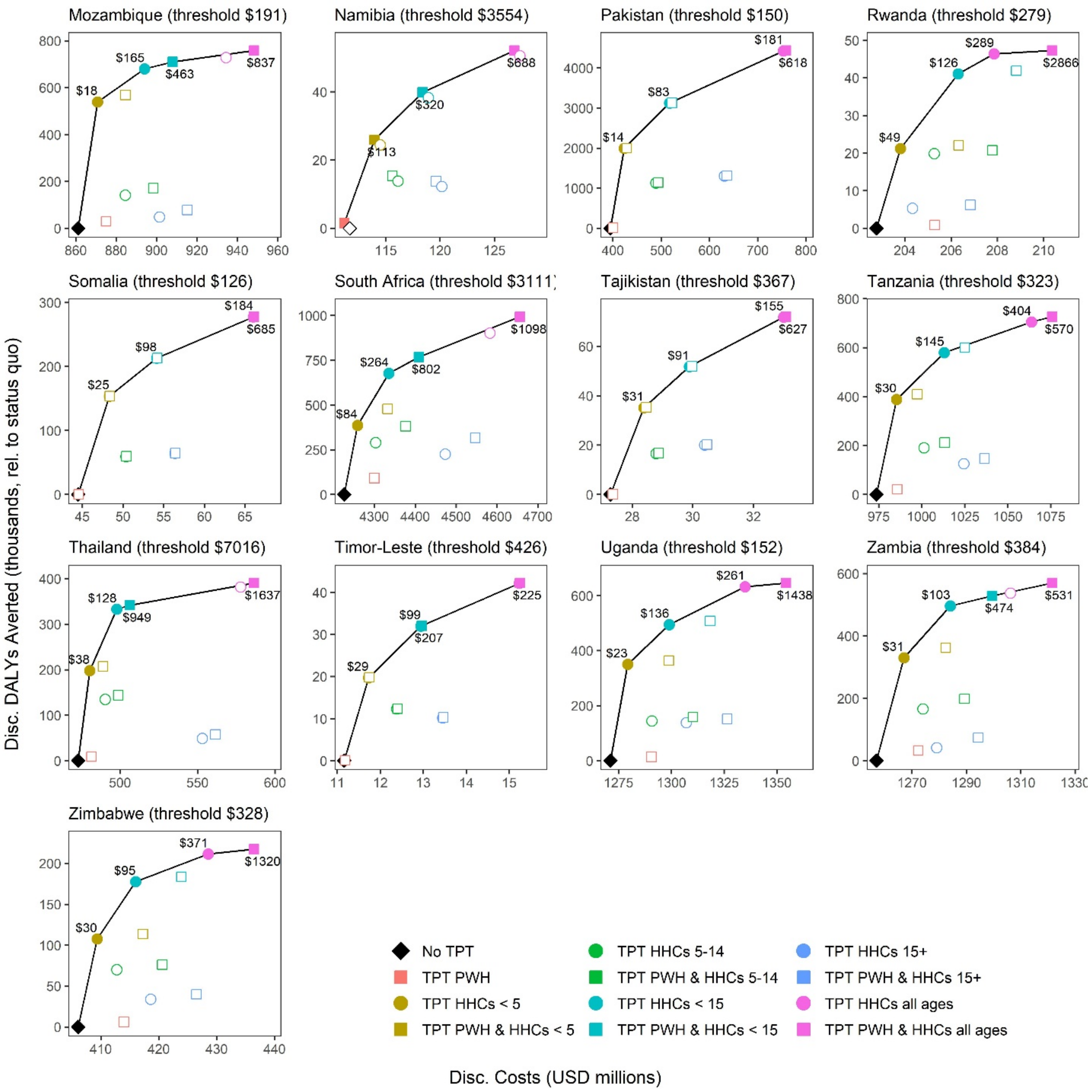

Figure shows incremental cost-effectiveness ratios of all combinations of strategies: 2 TPT scenarios (TPT or no TPT) and 4 target populations (contacts <5 years, contacts 5-14 years, contacts 15+ years and people with HIV: PWH). The incremental cost-effectiveness ratios are plotted on cost-effectiveness frontiers depicting discounted costs in 2020 USD on the x-axis and discounted disability adjusted life years averted relative to the No TPT scenario on the y-axis. Strategies appearing on the frontier are shaded, while dominated and extended dominated strategies are unshaded (white fill). Shapes indicate whether TPT for PWH is included in the strategy, and colors indicate different contact age combinations. GNI p.c. = Gross National Income per capita.

Appendix Figure 5: Cost-effectiveness of contact investigation with 3HP versus contact investigation without 3HP for household contacts in 29 countries

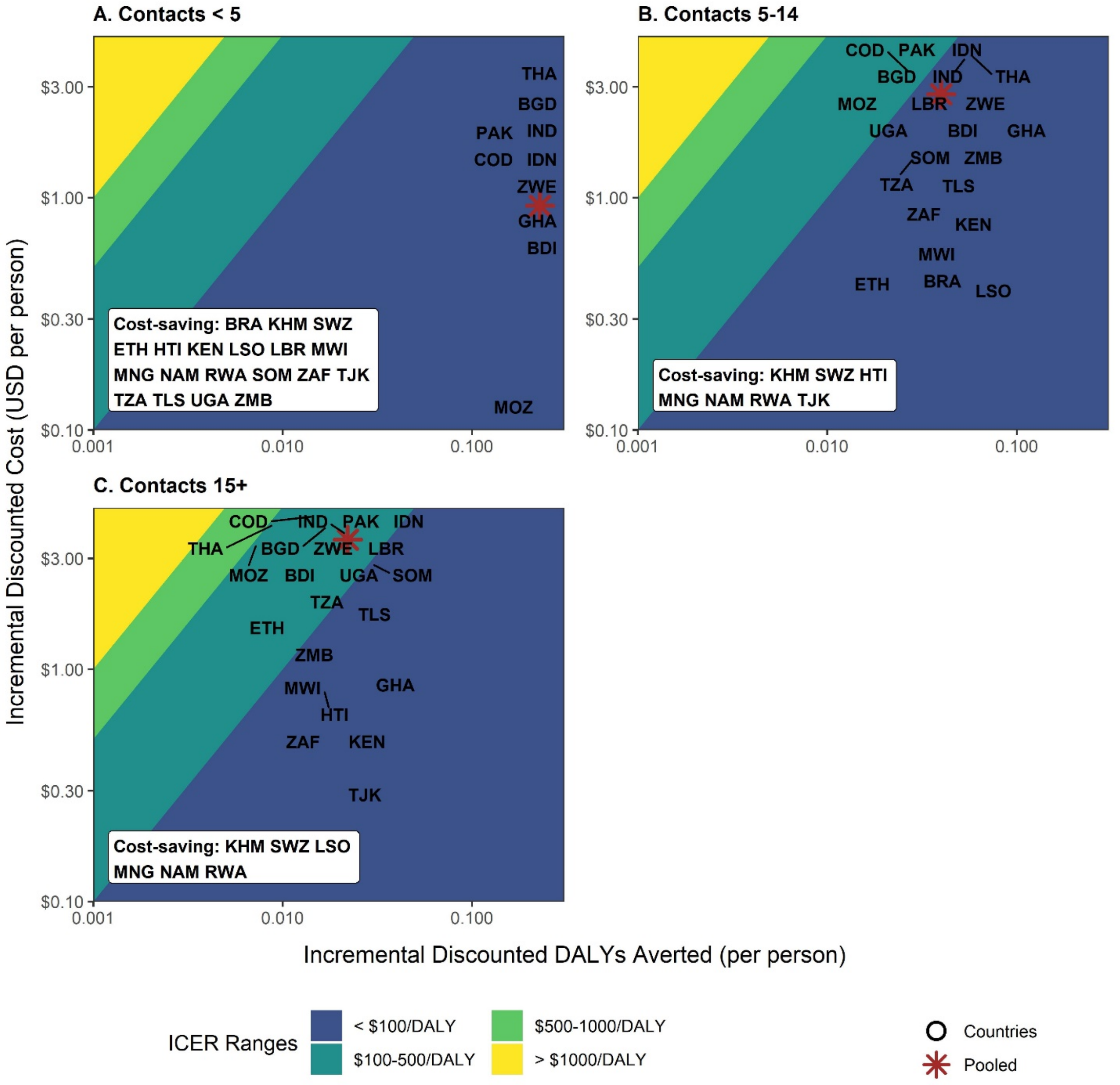

Each marker represents a country-specific estimate of discounted incremental disability-adjusted life years (DALYs) averted per household contact from implementing 3HP with contact investigation (x-axis) and corresponding discounted incremental costs (y-axis), compared to a scenario of contact investigation but no TPT. Each panel shows a different target population (A. household contacts < 5 years old, B. contacts 5-14 years old, C. contacts ≥ 15 years old). Countries are labeled by their 3-digit ISO codes, and averages across the 29 countries are designated via a dark red star. Filled areas of the graph indicate incremental cost-effectiveness ratios. Countries for which 3HP was estimated to be cost-saving compared to contact investigation alone are not labeled on the graphs, but are listed in the text boxes at the lower left of each panel.

Summary of sensitivity of results to individual model parameters

Sensitivity of the cost-effectiveness results to individual model parameters was assessed using linear regression metamodeling. Results were sensitive to three main model parameters. 3HP for household contacts was more cost-effective in settings with lower background notification rates and lower 3HP visit costs (Appendix Figures 6-9). For household contacts < 15, 3HP was generally more cost-effective in settings with lower TB treatment costs, because contact investigation tended to increase the numbers of people treated for TB. For household contacts ≥ 15 and PLWHA, 3HP was more cost-effective with higher TB treatment costs, because background treatment coverage ratios tend to be higher for older ages and thus the decrease in incidence from 3HP outweighed the increase in the notification rate from contact investigation (Appendix Figure 10). Cost-effectiveness for contacts < 15 was less sensitive to the price of 3HP than for contacts ≥ 15 and PLWHA, as 3HP made up a larger share of incremental costs for the latter populations (Appendix Figure 11).

Appendix Figure 6: Sensitivity of cost-effectiveness results for contacts < 5 years to parameter uncertainty

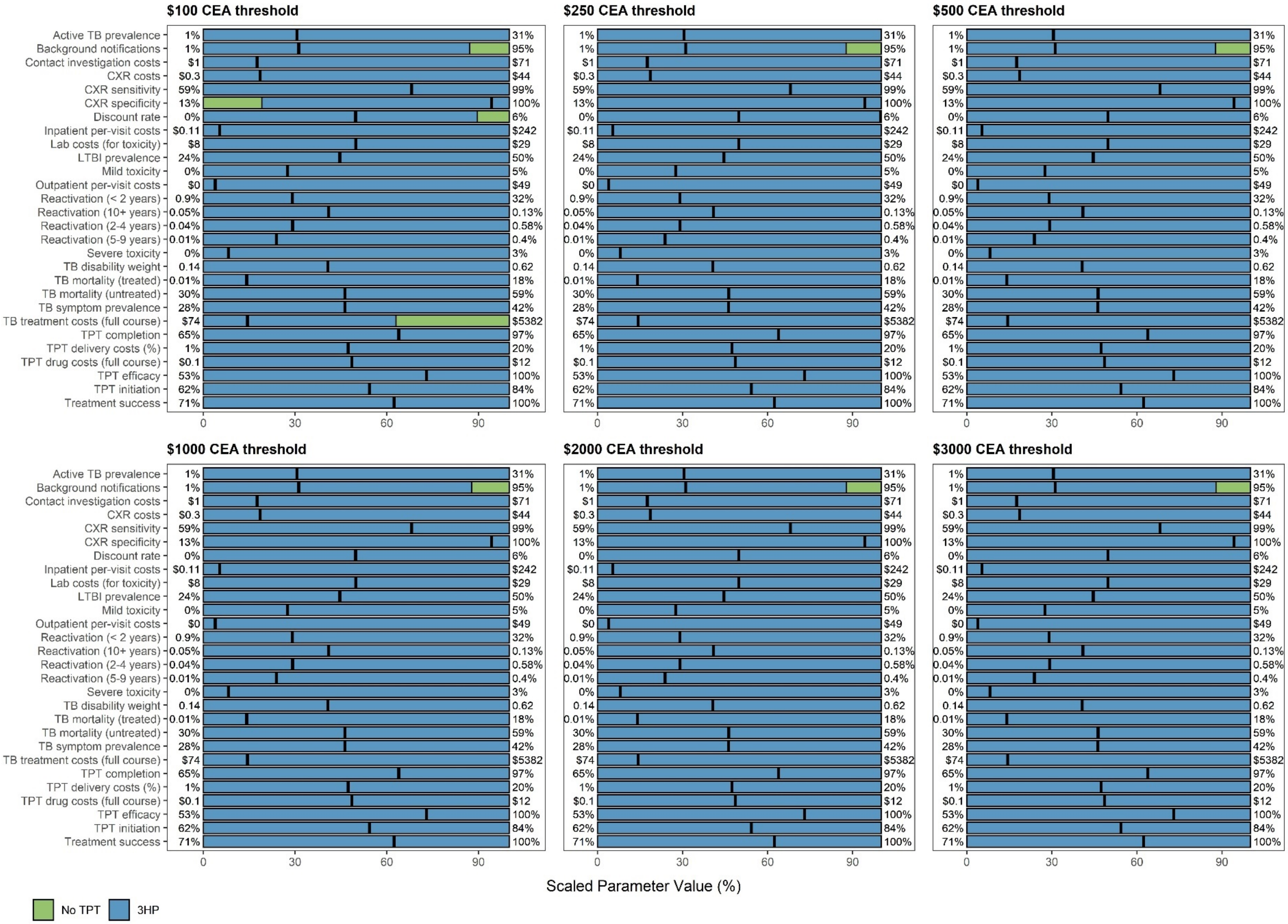

Each panel depicts the preferred strategy at a given cost-effectiveness threshold (“CEA threshold”) when all parameters are held at their mean values and a single parameter is adjusted over its full range. Thick black lines indicate the mean values for each parameter. The x-axis indicates the parameter value when it is scaled from 0% to 100%, with 0% representing the minimum, 100% representing the maximum, 50% representing the median, and so on. The x-axis locations where the graph changes color indicate the threshold of that parameter value at which the optimal strategy changes. Parameter minima and maxima are displayed on the plot margins. Parameter means and ranges of country-specific parameters (such as contact investigation costs and background notification rates) were obtained by pooling samples for each parameter separately over all 29 countries.

Appendix Figure 7: Sensitivity of cost-effectiveness results for contacts aged 5-14 years to parameter uncertainty

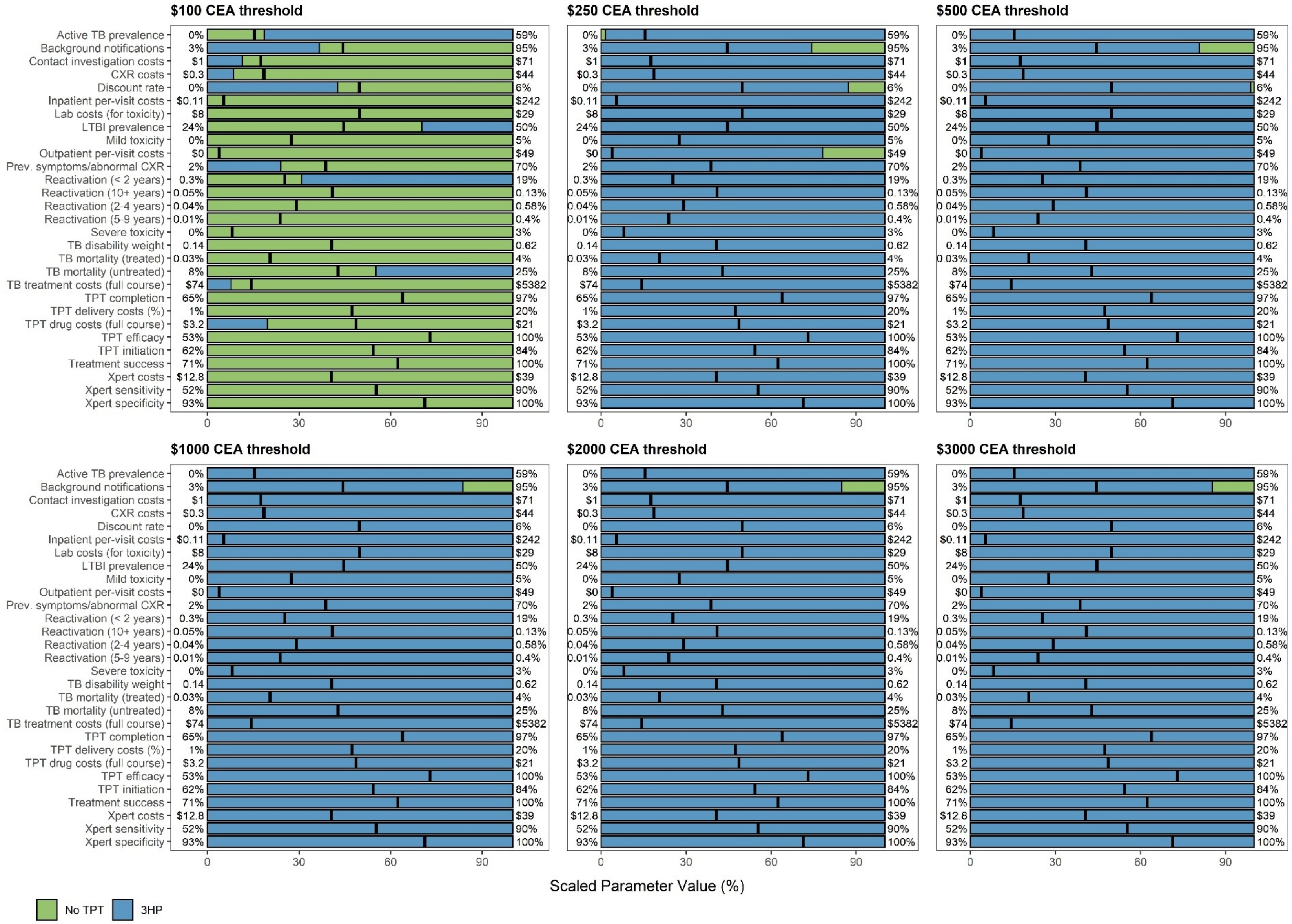

Each panel depicts the preferred strategy at a given cost-effectiveness threshold ("CEA threshold") when all parameters are held at their mean values and a single parameter is adjusted over its full range. Thick black lines indicate the mean values for each parameter. The x-axis indicates the parameter value when it is scaled from 0% to 100%, with 0% representing the minimum, 100% representing the maximum, 50% representing the median, and so on. The x-axis locations where the graph changes color indicate the threshold of that parameter value at which the optimal strategy changes. Parameter minima and maxima are displayed on the plot margins. Parameter means and ranges of country-specific parameters (such as contact investigation costs and background notification rates) were obtained by pooling samples for each parameter separately over all 29 countries.

Appendix Figure 8: Sensitivity of cost-effectiveness results for contacts aged 15 years and above to parameter uncertainty

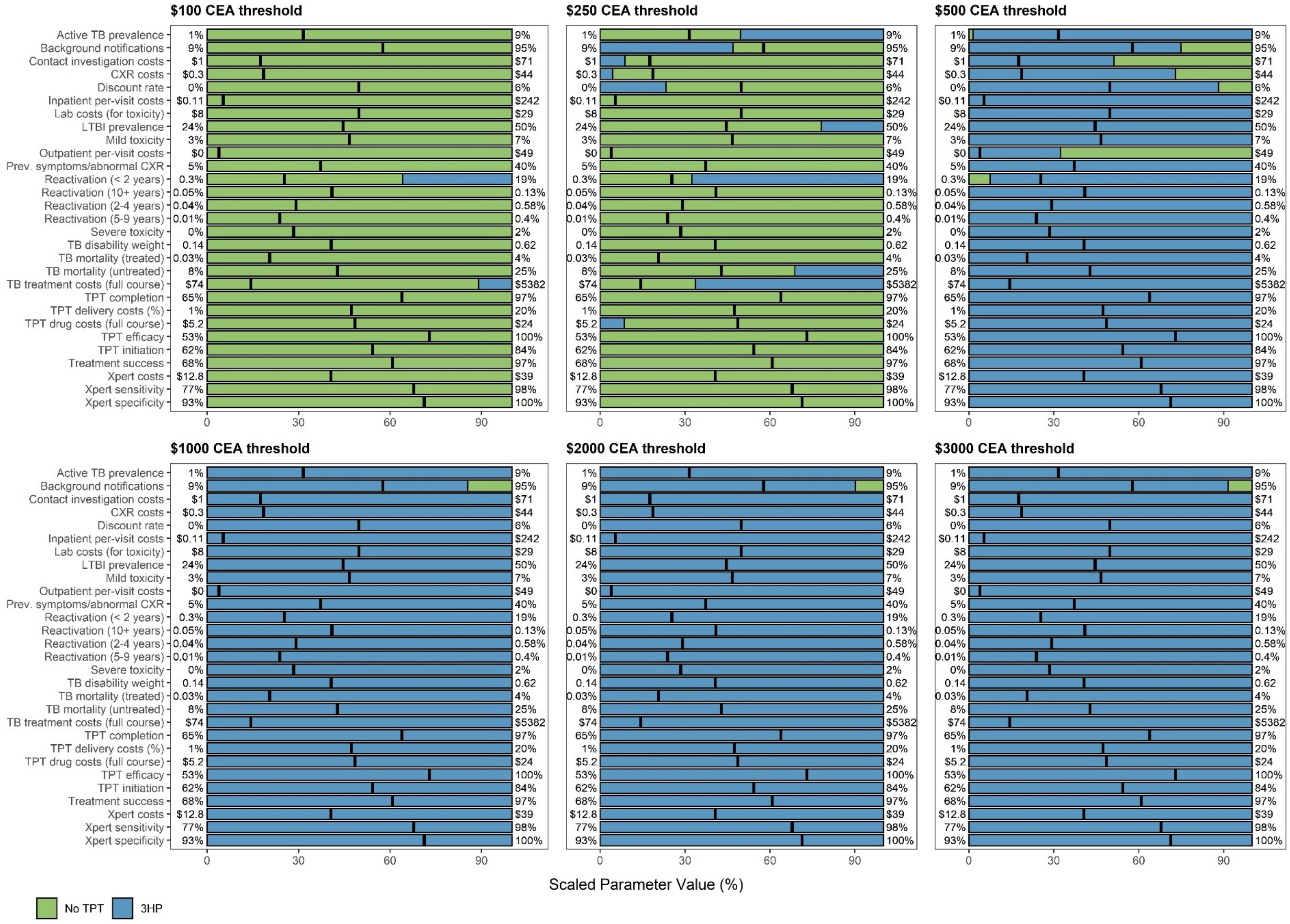

Each panel depicts the preferred strategy at a given cost-effectiveness threshold ("CEA threshold") when all parameters are held at their mean values and a single parameter is adjusted over its full range. Thick black lines indicate the mean values for each parameter. The x-axis indicates the parameter value when it is scaled from 0% to 100%, with 0% representing the minimum, 100% representing the maximum, 50% representing the median, and so on. The x-axis locations where the graph changes color indicate the threshold of that parameter value at which the optimal strategy changes. Parameter minima and maxima are displayed on the plot margins. Parameter means and ranges of country-specific parameters (such as contact investigation costs and background notification rates) were obtained by pooling samples for each parameter separately over all 29 countries.

Appendix Figure 9: Sensitivity of cost-effectiveness results for people living with HIV/AIDS to parameter uncertainty

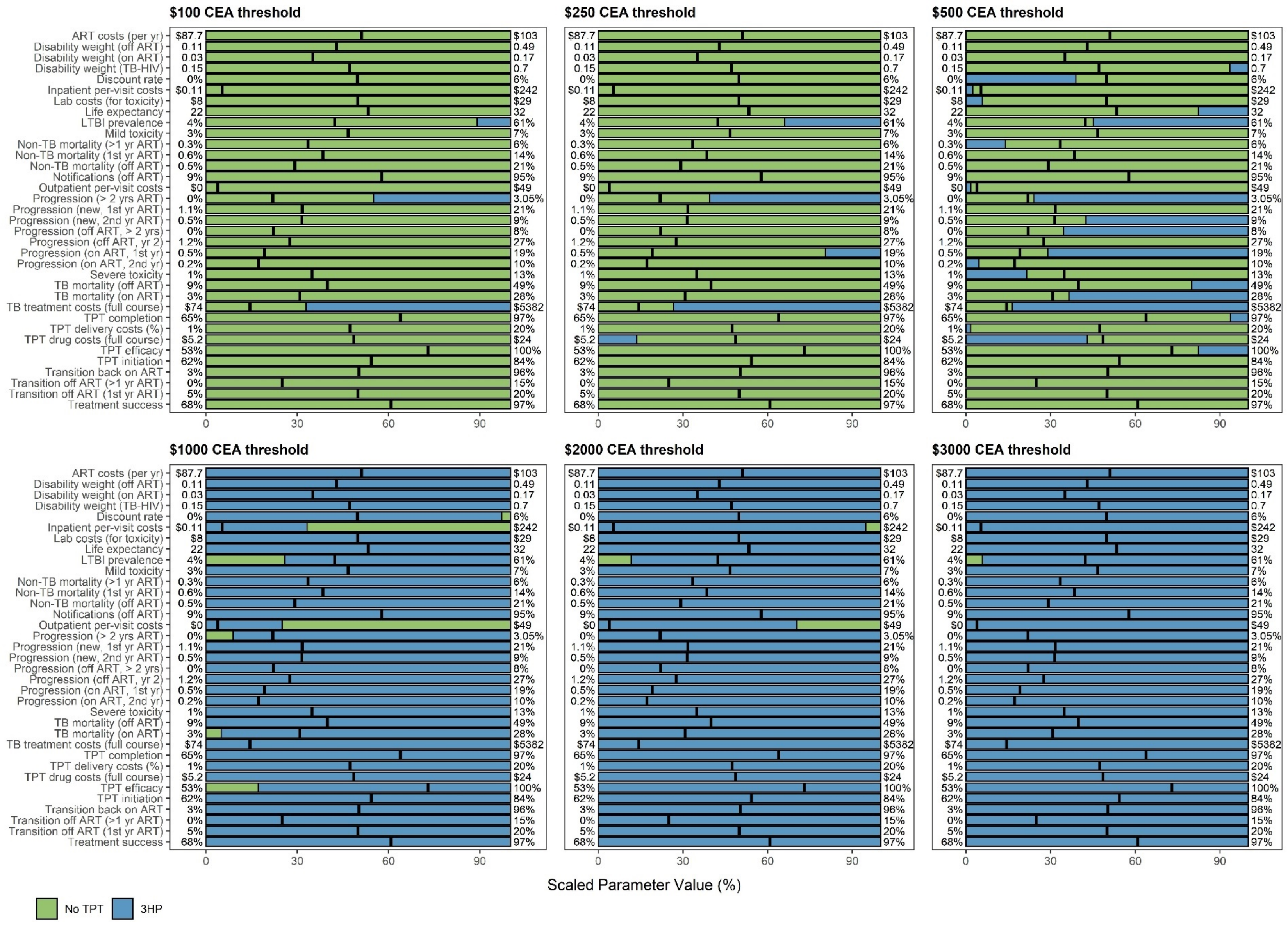

Each panel depicts the preferred strategy at a given cost-effectiveness threshold ("CEA threshold") when all parameters are held at their mean values and a single parameter is adjusted over its full range. Thick black lines indicate the mean values for each parameter. The x-axis indicates the parameter value when it is scaled from 0% to 100%, with 0% representing the minimum, 100% representing the maximum, 50% representing the median, and so on. The x-axis locations where the graph changes color indicate the threshold of that parameter value at which the optimal strategy changes. Parameter minima and maxima are displayed on the plot margins. Parameter means and ranges of country-specific parameters (such as contact investigation costs and background notification rates) were obtained by pooling samples for each parameter separately over all 29 countries.

Appendix Figure 10: Sensitivity of cost-effectiveness results to the cost of TB treatment

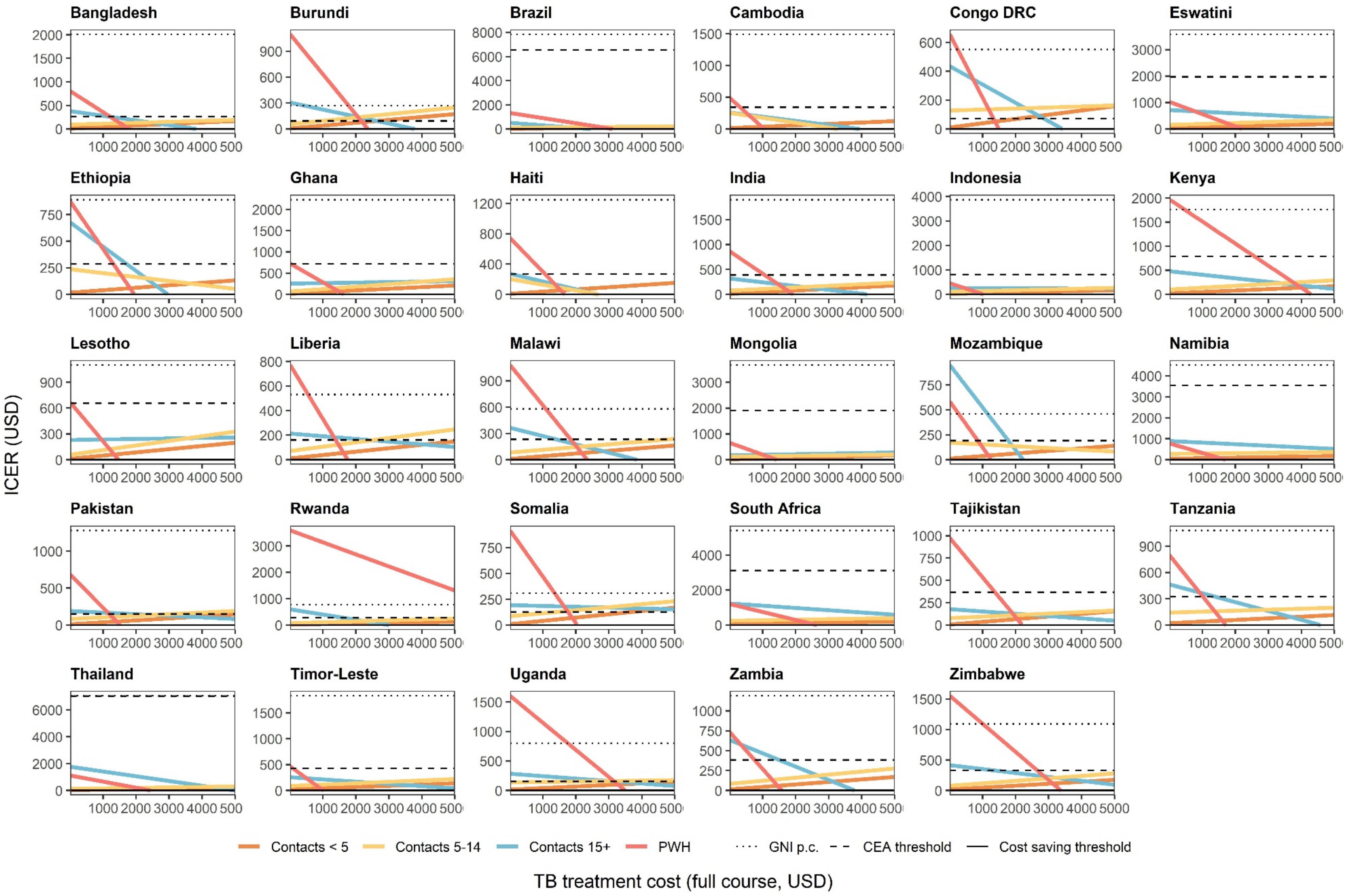

Figure shows how the incremental cost-effectiveness ratios (ICERs; y-axis) for each target population (colored lines, with household contact age groups displayed in years) vary when all parameters are held fixed at their mean values and the cost of a full course of TB treatment is varied from $10 to $5000 (x-axis). The points at which the colored lines cross the black horizonal lines indicate the 3HP price at which 3HP for a given population would be considered cost-effectiveness based on the main cost-effectiveness thresholds in each country ("CEA threshold"; dashed line), cost-effective based on a threshold equal to gross national income per capita (GNI p.c.; dotted line), and cost-saving (solid line with a y-intercept of $0), respectively. "PWH" = people with HIV.

Appendix Figure 11: Sensitivity of cost-effectiveness results to the price of 3HP

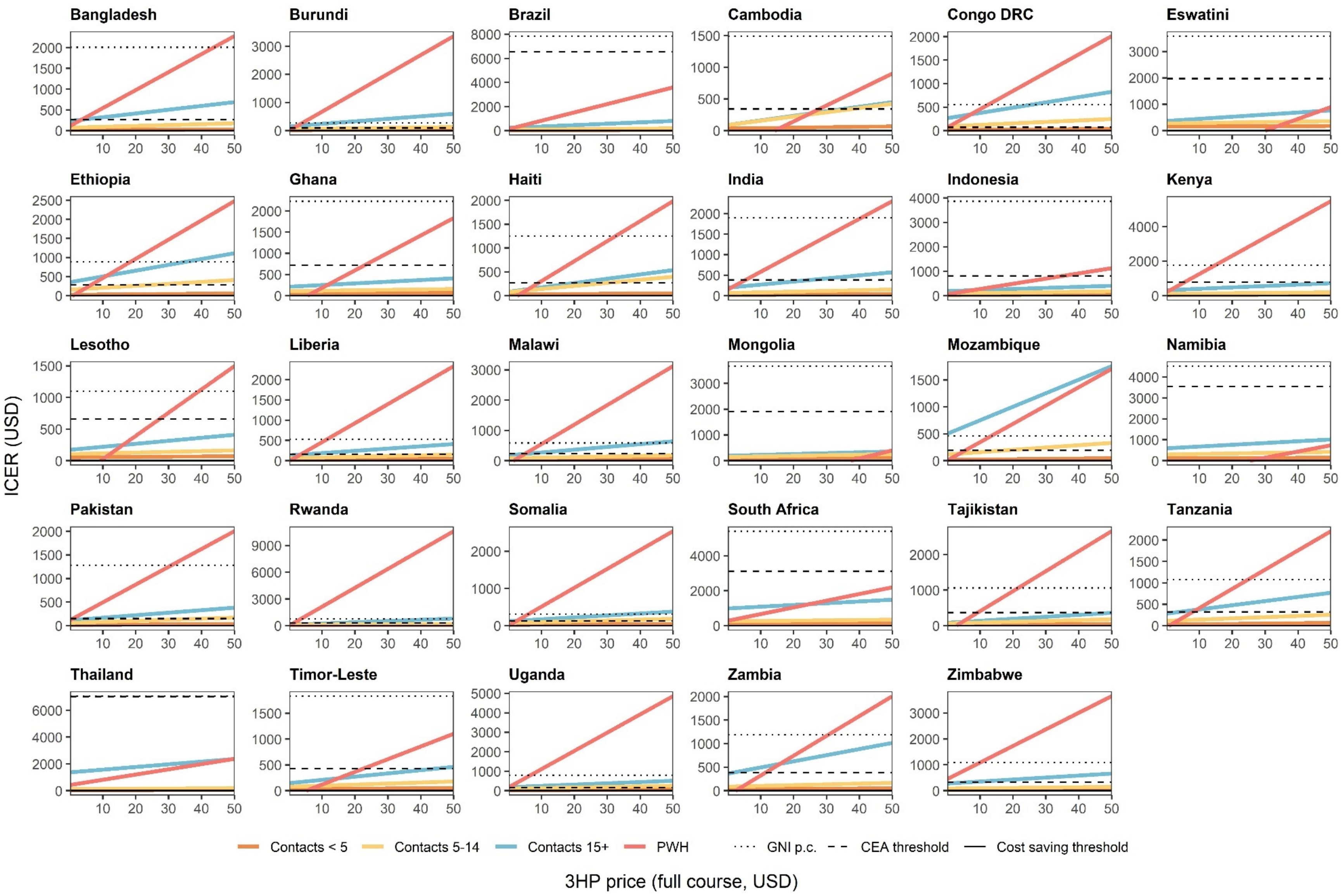

Figure shows how the incremental cost-effectiveness ratios (ICERs; y-axis) for each target population (colored lines, with household contact age groups displayed in years) vary when all parameters are held fixed at their mean values and the price of a full course of 3HP is varied from \$0·1 to \$50 (x-axis). The points at which the colored lines cross the black horizonal lines indicate the 3HP price at which 3HP for a given population would be considered cost-effectiveness based on the main cost-effectiveness thresholds in each country ("CEA threshold"; dashed line), cost-effective based on a threshold equal to gross national income per capita (GNI p.c.; dotted line), and cost-saving (solid line with a y-intercept of \$0), respectively. "PWH" = people with HIV.

Appendix Figure 12: Cost-effectiveness of TPT by population across cost-effectiveness thresholds

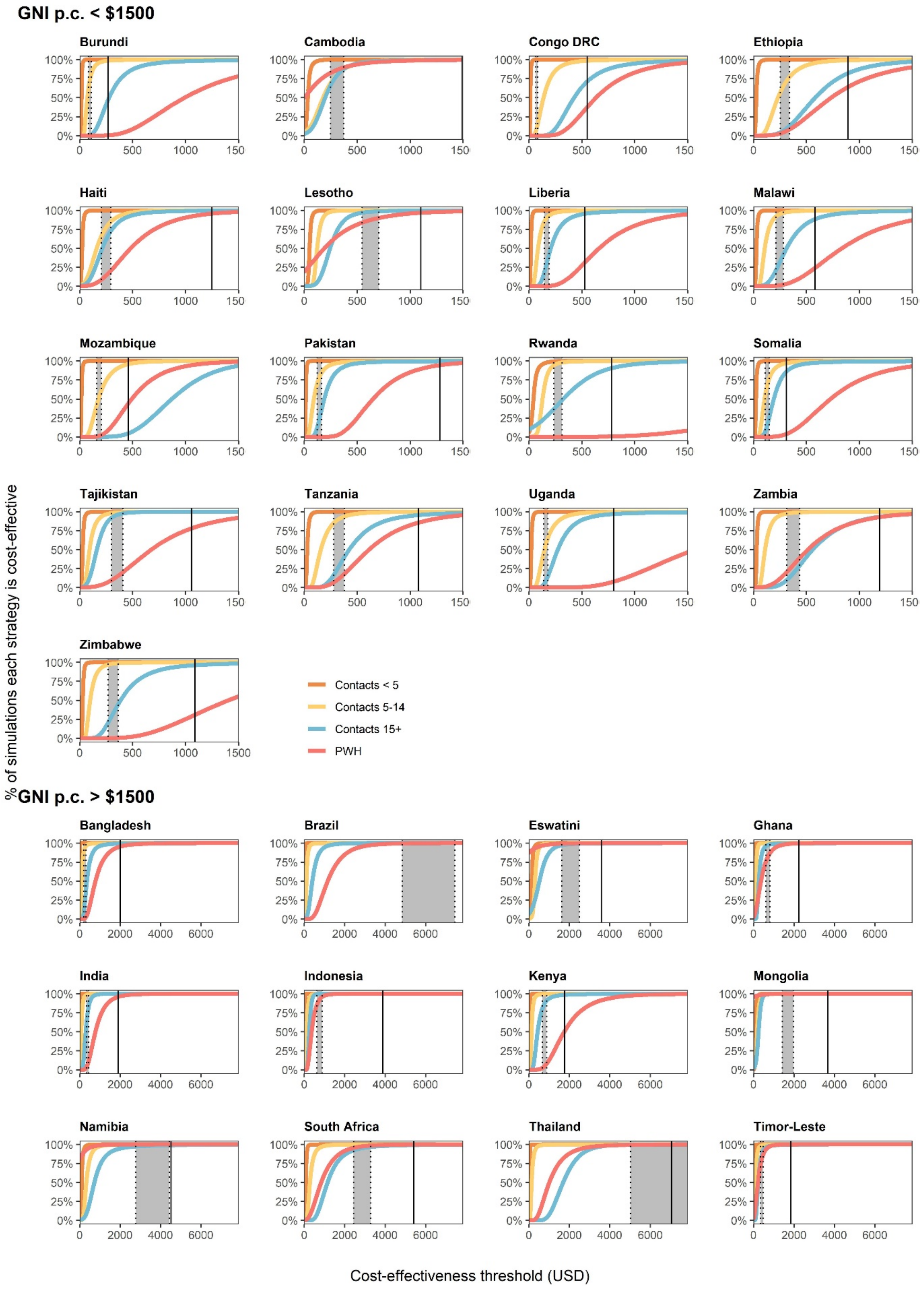

Figure shows the proportion of 50,000 probabilistic sensitivity analysis samples for which TPT for each of 4 target populations (shown via colored lines: contacts <5 years, contacts 5-14 years, contacts 15+ years, and people with HIV/PWH) was considered cost-effective (y-axis) over a range of cost-effectiveness thresholds (x-axis). Grey areas and dashed lines indicate country-specific willingness-to-pay ranges based on Ochalek et al. (see appendix page 14) – the upper dashed bound on the grey area is the base case willingness-to-pay threshold. Solid lines indicate each country's gross national income per capita. Countries are grouped by whether their gross national income per capita exceeds or falls below $3000 so that higher x-axis ranges can be shown for the higher-income countries.

Appendix Table 15: Cost-effectiveness of 3HP for household contacts and PLWHA in 29 countries – totals

Country	Inc. Disc Costs (USD millions)				Inc. Disc DALYs Averted (thousands)				ICERs			
	Contacts < 5 years	Contacts 5-14 years	Contacts 15+ years	PLWHA	Contacts < 5 years	Contacts 5-14 years	Contacts 15+ years	PLWHA	Contacts < 5 years	Contacts 5-14 years	Contacts 15+ years	PLWHA
Burundi	1·3	2·2	2·9	0·9	67·1	32·3	11·1	1·0	19	70	265	912
Bangladesh	23·4	46·3	150·8	4·4	1,520·4	494·3	436·8	6·1	15	94	345	725
Brazil	4·7	8·8	29·9	15·5	148·7	83·1	79·7	14·5	32	106	376	1069
Congo DRC	21·5	60·1	97·1	4·7	1,522·5	477·6	238·7	8·0	14	126	407	592
Ethiopia	14·4	29·1	53·2	3·5	518·4	135·0	96·6	5·2	28	216	550	674
Ghana	4·3	7·1	10·4	2·3	87·1	60·0	40·3	6·9	49	118	258	334
Haiti	1·1	1·2	3·8	0·4	42·7	7·5	18·8	0·8	26	153	201	449
Indonesia	36·9	93·9	393·2	3·4	1,308·2	847·8	1,599·8	9·8	28	111	246	349
India	177·5	321·9	1,230·0	14·3	9,446·5	3,919·2	4,216·8	19·6	19	82	292	733
Kenya	15·5	26·5	49·2	11·8	379·6	210·9	118·9	7·3	41	126	414	1624
Cambodia	2·9	2·9	11·2	0·0	76·7	17·2	60·6	1·9	38	169	184	-
Liberia	1·0	2·4	4·3	0·3	50·0	29·7	21·8	0·4	20	81	199	619
Lesotho	1·5	2·5	3·1	0·8	28·6	21·2	13·4	5·3	53	118	234	158
Mongolia	1·8	1·5	4·1	0·0	16·8	8·8	17·1	0·0	106	173	239	-
Mozambique	9·6	23·3	40·2	13·8	538·8	141·1	48·0	29·8	18	165	837	463
Malawi	3·7	5·3	7·3	2·4	125·7	52·4	24·6	3·1	29	101	295	777
Namibia	2·8	4·4	8·4	0·0	24·4	13·9	12·3	1·5	113	320	688	-
Pakistan	28·4	93·8	236·2	5·3	1,991·7	1,128·4	1,302·8	8·6	14	83	181	618
Rwanda	1·0	2·5	1·5	2·5	21·2	19·8	5·3	0·9	49	126	289	2866
Somalia	3·8	5·8	11·9	0·1	153·6	59·4	64·5	0·1	25	98	184	685
Eswatini	2·2	2·5	2·2	0·0	14·4	8·5	4·6	2·0	154	296	476	-
Thailand	7·5	17·3	80·0	8·5	197·9	134·9	48·8	8·9	38	128	1637	949
Tajikistan	1·1	1·5	3·1	0·1	35·1	16·6	20·1	0·1	31	91	155	627
Timor-Leste	0·6	1·2	2·3	0·0	19·7	12·3	10·2	0·1	29	99	225	207
Tanzania	11·7	27·6	50·7	12·0	388·4	191·0	125·7	21·0	30	145	404	570
Uganda	8·2	19·6	36·0	19·5	349·8	144·6	137·6	13·5	23	136	261	1438
South Africa	32·3	76·6	247·0	73·4	386·3	289·8	224·9	91·5	84	264	1098	802
Zambia	10·1	17·1	22·1	15·3	330·1	166·0	41·6	32·4	31	103	531	474
Zimbabwe	3·3	6·6	12·5	7·9	107·5	70·0	33·8	6·0	30	95	371	1320

ICERs (Incremental Cost-Effectiveness Ratios) are calculated as the ratio of incremental discounted costs and incremental discounted disability adjusted life years (DALYs) averted. PLWHA = People living with HIV/AIDS. All costs are presented in 2020 USD.

Appendix Table 16: Cost-effectiveness of 3HP for household contacts and PLWHA in 29 countries – per person

Country	Inc. Disc Costs (USD per person)				Inc. Disc DALYs Averted (per person)				ICERs			
	Contacts < 5 years	Contacts 5-14 years	Contacts 15+ years	PLWHA	Contacts < 5 years	Contacts 5-14 years	Contacts 15+ years	PLWHA	Contacts < 5 years	Contacts 5-14 years	Contacts 15+ years	PLWHA
Burundi	17·5	16·6	11·6	11·8	0·93	0·24	0·04	0·01	19	70	265	912
Bangladesh	17·4	15·7	14·2	14·0	1·13	0·17	0·04	0·02	15	94	345	725
Brazil	22·7	18·5	11·9	12·9	0·71	0·17	0·03	0·01	32	106	376	1069
Congo DRC	9·3	14·6	14·0	12·9	0·66	0·12	0·03	0·02	14	126	407	592
Ethiopia	14·4	15·1	13·6	10·9	0·52	0·07	0·02	0·02	28	216	550	674
Ghana	48·9	41·9	25·0	6·7	1·00	0·36	0·10	0·02	49	118	258	334
Haiti	16·3	8·2	8·4	8·7	0·63	0·05	0·04	0·02	26	153	201	449
Indonesia	18·6	22·1	20·9	13·0	0·66	0·20	0·09	0·04	28	111	246	349
India	18·3	17·2	14·3	14·4	0·97	0·21	0·05	0·02	19	82	292	733
Kenya	28·6	28·1	19·6	13·2	0·70	0·22	0·05	0·01	41	126	414	1624
Cambodia	19·1	8·6	9·7	-0·6	0·51	0·05	0·05	0·03	38	169	184	-
Liberia	11·9	16·7	13·8	11·1	0·61	0·21	0·07	0·02	20	81	199	619
Lesotho	45·6	36·5	18·4	3·7	0·86	0·31	0·08	0·02	53	118	234	158
Mongolia	91·0	27·0	27·6	-22·0	0·86	0·16	0·12	0·03	106	173	239	-
Mozambique	9·9	13·8	12·5	10·9	0·56	0·08	0·01	0·02	18	165	837	463
Malawi	22·0	18·7	12·3	10·0	0·74	0·19	0·04	0·01	29	101	295	777
Namibia	59·4	44·8	31·4	-9·2	0·52	0·14	0·05	0·03	113	320	688	-
Pakistan	8·7	13·6	13·0	12·5	0·61	0·16	0·07	0·02	14	83	181	618
Rwanda	24·1	29·0	7·8	11·9	0·49	0·23	0·03	0·00	49	126	289	2866
Somalia	17·5	16·7	13·8	10·7	0·71	0·17	0·08	0·02	25	98	184	685
Eswatini	110·5	63·3	22·6	-13·8	0·72	0·21	0·05	0·02	154	296	476	-
Thailand	33·2	34·4	29·5	21·0	0·88	0·27	0·02	0·02	38	128	1637	949
Tajikistan	25·0	14·1	10·5	9·0	0·80	0·16	0·07	0·01	31	91	155	627
Timor-Leste	16·2	17·9	13·6	6·5	0·57	0·18	0·06	0·03	29	99	225	207
Tanzania	13·3	18·5	16·0	10·6	0·44	0·13	0·04	0·02	30	145	404	570
Uganda	13·2	16·3	15·4	13·3	0·56	0·12	0·06	0·01	23	136	261	1438
South Africa	44·5	49·3	41·0	17·4	0·53	0·19	0·04	0·02	84	264	1098	802
Zambia	22·9	23·1	15·6	9·3	0·75	0·23	0·03	0·02	31	103	531	474
Zimbabwe	25·1	24·8	18·6	17·5	0·82	0·26	0·05	0·01	30	95	371	1320

ICERs (Incremental Cost-Effectiveness Ratios) are calculated as the ratio of incremental discounted costs and incremental discounted disability adjusted life years (DALYs) averted. PWH = People with HIV. All costs are presented in 2020 USD.

Appendix Table 17: Cost-effectiveness of 3HP for household contacts and PLWHA in 29 countries

Country	ICER (contacts < 5 years)	ICER (contacts 5-14 years)	ICER (contacts 15+ years)	ICER (PLWHA)	Cost-Effectiveness Threshold	Cost-Effectiveness Range	GNI p.c.
Burundi	19	70	265	912	92	84-110	270
Bangladesh	15	94	345	725	261	204-318	2,010
Brazil	32	106	376	1069	6,542	4839-7428	7,850
Congo DRC	14	126	407	592	71	65-82	550
Ethiopia	28	216	550	674	286	253-337	890
Ghana	49	118	258	334	721	609-811	2,230
Haiti	26	153	201	449	267	204-293	1,250
Indonesia	28	111	246	349	813	619-890	3,870
India	19	82	292	733	387	329-445	1,900
Kenya	41	126	414	1624	786	674-880	1,760
Cambodia	38	169	184	-	340	248-371	1,490
Liberia	20	81	199	619	160	145-190	530
Lesotho	53	118	234	158	657	546-704	1,100
Mongolia	106	173	239	-	1,909	1421-1990	3,670
Mozambique	18	165	837	463	191	162-206	460
Malawi	29	101	295	777	232	210-280	580
Namibia	113	320	688	-	3,554	2785-4448	4,520
Pakistan	14	83	181	618	150	122-163	1,280
Rwanda	49	126	289	2866	279	236-315	780
Somalia	25	98	184	685	126	111-146	310
Eswatini	154	296	476	-	1,975	1614-2507	3,580
Thailand	38	128	1637	949	7,016	5011-8018	7,050
Tajikistan	31	91	155	627	367	298-409	1,060
Timor-Leste	29	99	225	207	426	346-481	1,830
Tanzania	30	145	404	570	323	280-377	1,080
Uganda	23	136	261	1438	152	140-181	800
South Africa	84	264	1098	802	3,111	2432-3280	5,410
Zambia	31	103	531	474	384	315-433	1,190
Zimbabwe	30	95	371	1320	328	267-364	1,090

Incremental cost-effectiveness ratios (ICERs) are the same as those reported in Appendix Tables 4-5 and are repeated here for convenience. PLWHA = People living with HIV/AIDS, GNI p.c. = Gross National Income per capita. All costs are presented in 2020 USD.

Summary of sensitivity analysis results

Sensitivity analysis results are shown in Appendix Tables 18-21. In a sensitivity analysis that lowered eventual 3HP coverage to 50% (from 80% in the main analysis; "Lower TPT coverage"), 3HP was somewhat less cost-effective for PLWHA because it affected the annual proportions of PLWHA initiated on 3HP that were newly enrolled on ART vs. already established (newly enrolled PLWHA are at higher risk of progressing to active disease and are thus a more cost-effective population to cover). The cost-effectiveness of 3HP for household contacts was not affected by 3HP coverage.

In a sensitivity analysis that decreased ART turnover (resulting in overall higher adherence to ART; "Lower ART turnover"), 3HP was also somewhat less cost-effective for PLWHA – because PLWHA who discontinue ART are at greater risk of progressing to TB disease and thus receive more incremental benefit on average from taking 3HP.

1HP was found to be less cost-effective than 3HP in most settings, due to its increased drug costs. In some settings, the reduction in outpatient monitoring requirements and increased completion rates of 1HP outweighed the increased drug prices.

In an analysis that increased the number of required 3HP monitoring visits (with no improvement to 3HP outcomes), 3HP looked slightly less cost-effective but ICERs did not change substantially (generally < 10% increase for contacts).

The effect on cost-effectiveness of removing CXR from the screening algorithm for contacts aged 5 and above ("No CXR screen for HHCs") also varied by country. ICERs increased in countries where the costs of CXR relative to TB treatment were low and treatment coverage ratios were high because few savings were generated from the reduction in CXR costs, at the expense of fewer people with active TB linked to treatment.

3HP looked substantially more cost-effective when averted secondary transmission was factored into the analysis ("Secondary effects"). This was generally the sensitivity analysis that had the most dramatic impact on conclusions.

In most countries, the ICER of scaling up contact investigation without any TPT ("No TPT") was higher than the ICER of scaling up contact investigation with TPT, indicating that it is more cost-effective to include TPT when scaling up a contact investigation program.

Appendix Table 18: Sensitivity analysis results for contacts < 5 years

Country	Scenario	Inc. Disc. Costs (USD per person)	Inc. Disc. DALYs (per person)	ICER
Bangladesh	Base case	17·4	1·13	15
	Longer horizon	16·6	1·28	13
	Lower TPT coverage	9·7	0·63	15
	More monitoring visits	18·2	1·13	16
	No TPT	15·2	0·82	18
	Secondary effects (R=0·5)	16·6	1·14	14
	Secondary effects (R=1)	15·7	1·15	14
	1HP	22·6	1·14	20
Brazil	Base case	22·7	0·71	32
	Longer horizon	20·3	0·80	25
	Lower TPT coverage	12·6	0·40	32
	More monitoring visits	23·6	0·71	33
	No TPT	24·1	0·48	50
	Secondary effects (R=0·5)	20·3	0·73	28
	Secondary effects (R=1)	18·0	0·74	24
	1HP	27·8	0·72	38
Burundi	Base case	17·5	0·93	19
	Longer horizon	16·1	1·07	15
	Lower TPT coverage	9·7	0·52	19
	More monitoring visits	17·8	0·93	19
	No TPT	16·8	0·68	25
	Secondary effects (R=0·5)	16·3	0·95	17
	Secondary effects (R=1)	15·0	0·96	16
	1HP	22·9	0·94	24
Cambodia	Base case	19·1	0·51	38
	Longer horizon	14·5	0·56	26
	Lower TPT coverage	10·6	0·28	38
	More monitoring visits	20·0	0·51	39
	No TPT	26·8	0·35	75
	Secondary effects (R=0·5)	15·6	0·52	30
	Secondary effects (R=1)	12·1	0·53	23
	1HP	23·9	0·51	47
Congo DRC	Base case	9·3	0·66	14
	Longer horizon	8·7	0·74	12
	Lower TPT coverage	5·2	0·37	14
	More monitoring visits	9·7	0·66	15
	No TPT	8·0	0·47	17
	Secondary effects (R=0·5)	8·8	0·67	13
	Secondary effects (R=1)	8·3	0·68	12
	1HP	14·7	0·67	22
Eswatini	Base case	110·5	0·72	154
	Longer horizon	95·7	0·81	118
	Lower TPT coverage	61·4	0·40	154
	More monitoring visits	112·5	0·72	157
	No TPT	130·9	0·51	257
	Secondary effects (R=0·5)	99·6	0·73	136
	Secondary effects (R=1)	88·7	0·74	120
	1HP	115·0	0·73	158
Ethiopia	Base case	14·4	0·52	28
	Longer horizon	12·2	0·57	21
	Lower TPT coverage	8·0	0·29	28
	More monitoring visits	15·0	0·52	29
	No TPT	16·9	0·36	47
	Secondary effects (R=0·5)	12·3	0·53	23
	Secondary effects (R=1)	10·1	0·54	19
	1HP	19·6	0·53	37
Ghana	Base case	48·9	1·00	49
	Longer horizon	46·8	1·17	40
	Lower TPT coverage	27·2	0·55	49

Country	Scenario	Inc. Disc. Costs (USD per person)	Inc. Disc. DALYs (per person)	ICER
	More monitoring visits	49·4	1·00	50
	No TPT	48·1	0·69	70
	Secondary effects (R=0·5)	47·4	1·02	47
	Secondary effects (R=1)	45·9	1·04	44
	1HP	54·2	1·01	54
Haiti	Base case	16·3	0·63	26
	Longer horizon	13·6	0·69	20
	Lower TPT coverage	9·1	0·35	26
	More monitoring visits	17·0	0·63	27
	No TPT	19·1	0·43	44
	Secondary effects (R=0·5)	14·1	0·64	22
	Secondary effects (R=1)	11·9	0·65	18
	1HP	21·4	0·63	34
India	Base case	18·3	0·97	19
	Longer horizon	17·3	1·10	16
	Lower TPT coverage	10·1	0·54	19
	More monitoring visits	19·5	0·97	20
	No TPT	16·1	0·69	23
	Secondary effects (R=0·5)	17·4	0·99	18
	Secondary effects (R=1)	16·5	1·00	16
	1HP	23·6	0·98	24
Indonesia	Base case	18·6	0·66	28
	Longer horizon	17·7	0·74	24
	Lower TPT coverage	10·4	0·37	28
	More monitoring visits	20·1	0·66	30
	No TPT	17·2	0·46	38
	Secondary effects (R=0·5)	18·1	0·68	27
	Secondary effects (R=1)	17·6	0·69	25
	1HP	23·9	0·67	36
Kenya	Base case	28·6	0·70	41
	Longer horizon	25·6	0·79	32
	Lower TPT coverage	15·9	0·39	41
	More monitoring visits	29·7	0·70	42
	No TPT	30·8	0·49	62
	Secondary effects (R=0·5)	26·3	0·71	37
	Secondary effects (R=1)	24·1	0·73	33
	1HP	33·8	0·71	48
Lesotho	Base case	45·6	0·86	53
	Longer horizon	42·3	0·99	43
	Lower TPT coverage	25·3	0·48	53
	More monitoring visits	46·3	0·86	54
	No TPT	47·1	0·61	78
	Secondary effects (R=0·5)	43·4	0·88	49
	Secondary effects (R=1)	41·3	0·90	46
	1HP	50·7	0·87	58
Liberia	Base case	11·9	0·61	20
	Longer horizon	10·5	0·68	15
	Lower TPT coverage	6·6	0·34	20
	More monitoring visits	12·2	0·61	20
	No TPT	12·4	0·43	29
	Secondary effects (R=0·5)	11·1	0·63	18
	Secondary effects (R=1)	10·3	0·64	16
	1HP	17·3	0·62	28
Malawi	Base case	22·0	0·74	29
	Longer horizon	19·3	0·84	23
	Lower TPT coverage	12·2	0·41	29
	More monitoring visits	22·4	0·74	30
	No TPT	23·9	0·53	45
	Secondary effects (R=0·5)	19·8	0·76	26
	Secondary effects (R=1)	17·7	0·77	23
	1HP	27·3	0·75	36

Country	Scenario	Inc. Disc. Costs (USD per person)	Inc. Disc. DALYs (per person)	ICER
Mongolia	Base case	91·0	0·86	106
	Longer horizon	80·2	0·97	83
	Lower TPT coverage	50·5	0·48	106
	More monitoring visits	92·3	0·86	108
	No TPT	103·8	0·62	167
	Secondary effects (R=0·5)	86·1	0·87	99
	Secondary effects (R=1)	81·2	0·89	91
	1HP	95·6	0·87	110
Mozambique	Base case	9·9	0·56	18
	Longer horizon	8·8	0·62	14
	Lower TPT coverage	5·5	0·31	18
	More monitoring visits	10·4	0·56	19
	No TPT	9·8	0·39	25
	Secondary effects (R=0·5)	8·8	0·57	15
	Secondary effects (R=1)	7·6	0·58	13
	1HP	15·3	0·56	27
Namibia	Base case	59·4	0·52	113
	Longer horizon	49·4	0·58	85
	Lower TPT coverage	33·0	0·29	113
	More monitoring visits	62·0	0·52	118
	No TPT	77·3	0·37	209
	Secondary effects (R=0·5)	51·8	0·54	97
	Secondary effects (R=1)	44·2	0·55	81
	1HP	63·9	0·53	121
Pakistan	Base case	8·7	0·61	14
	Longer horizon	8·2	0·68	12
	Lower TPT coverage	4·8	0·34	14
	More monitoring visits	9·4	0·61	15
	No TPT	7·0	0·43	16
	Secondary effects (R=0·5)	8·4	0·63	13
	Secondary effects (R=1)	8·1	0·64	13
	1HP	14·0	0·62	23
Rwanda	Base case	24·1	0·49	49
	Longer horizon	18·0	0·55	33
	Lower TPT coverage	13·4	0·27	49
	More monitoring visits	24·6	0·49	50
	No TPT	36·3	0·35	104
	Secondary effects (R=0·5)	18·1	0·50	36
	Secondary effects (R=1)	12·2	0·52	24
	1HP	28·9	0·50	58
Somalia	Base case	17·5	0·71	25
	Longer horizon	15·4	0·79	19
	Lower TPT coverage	9·7	0·39	25
	More monitoring visits	18·4	0·71	26
	No TPT	18·1	0·50	36
	Secondary effects (R=0·5)	16·4	0·72	23
	Secondary effects (R=1)	15·2	0·74	21
	1HP	22·9	0·72	32
South Africa	Base case	44·5	0·53	84
	Longer horizon	40·8	0·59	69
	Lower TPT coverage	24·7	0·30	84
	More monitoring visits	48·4	0·53	91
	No TPT	47·1	0·37	129
	Secondary effects (R=0·5)	41·5	0·55	76
	Secondary effects (R=1)	38·5	0·56	69
	1HP	49·6	0·54	92
Tajikistan	Base case	25·0	0·80	31
	Longer horizon	21·6	0·91	24
	Lower TPT coverage	13·9	0·44	31
	More monitoring visits	25·5	0·80	32
	No TPT	27·4	0·58	48

Country	Scenario	Inc. Disc. Costs (USD per person)	Inc. Disc. DALYs (per person)	ICER
	Secondary effects (R=0·5)	22·8	0·81	28
	Secondary effects (R=1)	20·7	0·82	25
	1HP	30·1	0·81	37
Tanzania	Base case	13·3	0·44	30
	Longer horizon	11·2	0·49	23
	Lower TPT coverage	7·4	0·25	30
	More monitoring visits	14·0	0·44	32
	No TPT	15·6	0·31	50
	Secondary effects (R=0·5)	11·7	0·46	26
	Secondary effects (R=1)	10·0	0·47	21
	1HP	18·6	0·45	42
Thailand	Base case	33·2	0·88	38
	Longer horizon	32·0	0·99	32
	Lower TPT coverage	18·5	0·49	38
	More monitoring visits	36·3	0·88	41
	No TPT	30·1	0·63	48
	Secondary effects (R=0·5)	31·6	0·89	36
	Secondary effects (R=1)	30·1	0·90	33
	1HP	38·5	0·89	43
Timor-Leste	Base case	16·2	0·57	29
	Longer horizon	14·0	0·63	22
	Lower TPT coverage	9·0	0·32	29
	More monitoring visits	17·1	0·57	30
	No TPT	18·4	0·40	46
	Secondary effects (R=0·5)	14·6	0·58	25
	Secondary effects (R=1)	13·0	0·60	22
	1HP	21·3	0·58	37
Uganda	Base case	13·2	0·56	23
	Longer horizon	11·6	0·63	19
	Lower TPT coverage	7·3	0·31	23
	More monitoring visits	13·8	0·56	24
	No TPT	14·0	0·39	36
	Secondary effects (R=0·5)	12·2	0·58	21
	Secondary effects (R=1)	11·2	0·59	19
	1HP	18·5	0·57	32
Zambia	Base case	22·9	0·75	31
	Longer horizon	20·6	0·84	24
	Lower TPT coverage	12·7	0·41	31
	More monitoring visits	23·4	0·75	31
	No TPT	24·0	0·53	45
	Secondary effects (R=0·5)	20·9	0·76	28
	Secondary effects (R=1)	18·8	0·77	24
	1HP	28·2	0·75	37
Zimbabwe	Base case	25·1	0·82	30
	Longer horizon	23·1	0·94	25
	Lower TPT coverage	13·9	0·46	30
	More monitoring visits	27·3	0·82	33
	No TPT	23·7	0·59	40
	Secondary effects (R=0·5)	23·6	0·84	28
	Secondary effects (R=1)	22·1	0·85	26
	1HP	30·5	0·83	37

Table shows the discounted disability adjusted life years averted (DALYs), incremental discounted costs (in 2020 USD), and incremental cost-effectiveness ratios of TPT for contacts < 5 vs. no TPT for contacts < 5, by country and for 7 different sensitivity analyses (compared to the base case).

Appendix Table 19: Sensitivity analysis results for contacts aged 5-14 years

Country	Scenario	Inc. Disc. Costs (USD per person)	Inc. Disc. DALYs (per person)	ICER
Bangladesh	Base case	15·7	0·17	94
	Longer horizon	14·6	0·19	75
	Lower TPT coverage	8·7	0·09	94
	More monitoring visits	16·6	0·17	99
	No CXR screen for HHCs	11·9	0·12	103
	No TPT	12·6	0·13	95
	Secondary effects (R=0·5)	15·3	0·17	88
	Secondary effects (R=1)	14·8	0·18	82
	1HP	19·1	0·17	113
Brazil	Base case	18·5	0·17	106
	Longer horizon	14·9	0·21	72
	Lower TPT coverage	10·3	0·10	106
	More monitoring visits	19·4	0·17	111
	No CXR screen for HHCs	12·0	0·12	98
	No TPT	18·1	0·13	139
	Secondary effects (R=0·5)	17·2	0·18	95
	Secondary effects (R=1)	15·9	0·19	85
	1HP	21·8	0·18	123
Burundi	Base case	16·6	0·24	70
	Longer horizon	14·2	0·29	48
	Lower TPT coverage	9·2	0·13	70
	More monitoring visits	16·9	0·24	71
	No CXR screen for HHCs	9·2	0·16	56
	No TPT	14·2	0·19	75
	Secondary effects (R=0·5)	15·9	0·25	65
	Secondary effects (R=1)	15·3	0·25	61
	1HP	20·1	0·24	83
Cambodia	Base case	8·6	0·05	169
	Longer horizon	5·2	0·06	92
	Lower TPT coverage	4·8	0·03	169
	More monitoring visits	9·6	0·05	188
	No CXR screen for HHCs	4·1	0·04	114
	No TPT	12·8	0·04	340
	Secondary effects (R=0·5)	6·7	0·06	115
	Secondary effects (R=1)	4·9	0·07	74
	1HP	11·7	0·05	227
Congo DRC	Base case	14·6	0·12	126
	Longer horizon	13·9	0·13	105
	Lower TPT coverage	8·1	0·06	126
	More monitoring visits	15·0	0·12	129
	No CXR screen for HHCs	8·8	0·08	109
	No TPT	11·2	0·09	125
	Secondary effects (R=0·5)	14·4	0·12	117
	Secondary effects (R=1)	14·1	0·13	110
	1HP	18·2	0·12	155
Eswatini	Base case	63·3	0·21	296
	Longer horizon	42·2	0·26	163
	Lower TPT coverage	35·2	0·12	296
	More monitoring visits	65·3	0·21	305
	No CXR screen for HHCs	33·7	0·15	229
	No TPT	78·3	0·17	470
	Secondary effects (R=0·5)	57·4	0·22	261
	Secondary effects (R=1)	51·5	0·23	227
	1HP	66·0	0·22	306
Ethiopia	Base case	15·1	0·07	216
	Longer horizon	13·1	0·08	168
	Lower TPT coverage	8·4	0·04	216
	More monitoring visits	15·7	0·07	224
	No CXR screen for HHCs	8·2	0·05	167

Country	Scenario	Inc. Disc. Costs (USD per person)	Inc. Disc. DALYs (per person)	ICER
	No TPT	14·7	0·05	279
	Secondary effects (R=0·5)	14·0	0·08	185
	Secondary effects (R=1)	12·8	0·08	159
	1HP	18·5	0·07	261
Ghana	Base case	41·9	0·36	118
	Longer horizon	36·4	0·50	73
	Lower TPT coverage	23·3	0·20	118
	More monitoring visits	42·5	0·36	119
	No CXR screen for HHCs	26·3	0·25	106
	No TPT	40·0	0·27	146
	Secondary effects (R=0·5)	41·1	0·37	112
	Secondary effects (R=1)	40·3	0·38	107
	1HP	45·3	0·36	126
Haiti	Base case	8·2	0·05	153
	Longer horizon	5·9	0·06	98
	Lower TPT coverage	4·6	0·03	153
	More monitoring visits	8·9	0·05	167
	No CXR screen for HHCs	3·8	0·04	101
	No TPT	9·3	0·04	239
	Secondary effects (R=0·5)	7·0	0·06	116
	Secondary effects (R=1)	5·9	0·07	87
	1HP	11·4	0·05	211
India	Base case	17·2	0·21	82
	Longer horizon	15·5	0·25	62
	Lower TPT coverage	9·5	0·12	82
	More monitoring visits	18·4	0·21	88
	No CXR screen for HHCs	12·7	0·14	88
	No TPT	14·0	0·16	86
	Secondary effects (R=0·5)	16·7	0·22	77
	Secondary effects (R=1)	16·2	0·22	72
	1HP	20·5	0·21	97
Indonesia	Base case	22·1	0·20	111
	Longer horizon	20·7	0·24	87
	Lower TPT coverage	12·3	0·11	111
	More monitoring visits	23·6	0·20	118
	No CXR screen for HHCs	17·5	0·14	126
	No TPT	18·6	0·15	122
	Secondary effects (R=0·5)	21·8	0·21	105
	Secondary effects (R=1)	21·6	0·22	99
	1HP	25·5	0·20	126
Kenya	Base case	28·1	0·22	126
	Longer horizon	23·6	0·28	86
	Lower TPT coverage	15·6	0·12	126
	More monitoring visits	29·2	0·22	131
	No CXR screen for HHCs	17·2	0·15	112
	No TPT	27·2	0·17	156
	Secondary effects (R=0·5)	26·9	0·23	117
	Secondary effects (R=1)	25·8	0·24	108
	1HP	31·4	0·23	140
Lesotho	Base case	36·5	0·31	118
	Longer horizon	29·0	0·41	71
	Lower TPT coverage	20·3	0·17	118
	More monitoring visits	37·2	0·31	121
	No CXR screen for HHCs	21·3	0·21	100
	No TPT	36·1	0·24	151
	Secondary effects (R=0·5)	35·3	0·32	112
	Secondary effects (R=1)	34·2	0·32	105
	1HP	39·8	0·31	128
Liberia	Base case	16·7	0·21	81
	Longer horizon	14·7	0·25	58
	Lower TPT coverage	9·3	0·11	81

Country	Scenario	Inc. Disc. Costs (USD per person)	Inc. Disc. DALYs (per person)	ICER
	More monitoring visits	16·9	0·21	82
	No CXR screen for HHCs	9·6	0·14	67
	No TPT	14·2	0·16	88
	Secondary effects (R=0·5)	16·2	0·22	75
	Secondary effects (R=1)	15·8	0·22	71
	1HP	20·1	0·21	97
Malawi	Base case	18·7	0·19	101
	Longer horizon	14·9	0·22	67
	Lower TPT coverage	10·4	0·10	101
	More monitoring visits	19·2	0·19	103
	No CXR screen for HHCs	10·1	0·13	79
	No TPT	18·1	0·15	124
	Secondary effects (R=0·5)	17·6	0·19	91
	Secondary effects (R=1)	16·5	0·20	82
	1HP	22·1	0·19	118
Mongolia	Base case	27·0	0·16	173
	Longer horizon	14·5	0·18	80
	Lower TPT coverage	15·0	0·09	173
	More monitoring visits	28·4	0·16	182
	No CXR screen for HHCs	12·6	0·11	117
	No TPT	41·3	0·12	339
	Secondary effects (R=0·5)	24·3	0·17	147
	Secondary effects (R=1)	21·6	0·18	124
	1HP	29·7	0·16	189
Mozambique	Base case	13·8	0·08	165
	Longer horizon	12·6	0·09	136
	Lower TPT coverage	7·7	0·05	165
	More monitoring visits	14·3	0·08	171
	No CXR screen for HHCs	7·8	0·06	134
	No TPT	11·2	0·06	177
	Secondary effects (R=0·5)	13·2	0·09	148
	Secondary effects (R=1)	12·5	0·09	133
	1HP	17·3	0·08	205
Namibia	Base case	44·8	0·14	320
	Longer horizon	33·2	0·16	205
	Lower TPT coverage	24·9	0·08	320
	More monitoring visits	47·5	0·14	339
	No CXR screen for HHCs	25·6	0·10	265
	No TPT	55·3	0·11	507
	Secondary effects (R=0·5)	40·8	0·15	278
	Secondary effects (R=1)	36·7	0·15	239
	1HP	47·7	0·14	337
Pakistan	Base case	13·6	0·16	83
	Longer horizon	12·9	0·19	67
	Lower TPT coverage	7·5	0·09	83
	More monitoring visits	14·3	0·16	88
	No CXR screen for HHCs	10·2	0·11	91
	No TPT	9·7	0·13	77
	Secondary effects (R=0·5)	13·4	0·17	78
	Secondary effects (R=1)	13·2	0·18	73
	1HP	17·0	0·16	103
Rwanda	Base case	29·0	0·23	126
	Longer horizon	19·9	0·28	71
	Lower TPT coverage	16·1	0·13	126
	More monitoring visits	29·6	0·23	128
	No CXR screen for HHCs	14·8	0·16	93
	No TPT	32·6	0·18	179
	Secondary effects (R=0·5)	25·8	0·24	109
	Secondary effects (R=1)	22·6	0·24	93
	1HP	32·2	0·23	138
Somalia	Base case	16·7	0·17	98

Country	Scenario	Inc. Disc. Costs (USD per person)	Inc. Disc. DALYs (per person)	ICER
	Longer horizon	13·7	0·20	67
	Lower TPT coverage	9·3	0·09	98
	More monitoring visits	17·6	0·17	104
	No CXR screen for HHCs	9·0	0·12	77
	No TPT	15·0	0·13	114
	Secondary effects (R=0·5)	16·1	0·18	90
	Secondary effects (R=1)	15·5	0·19	82
	1HP	20·1	0·17	118
South Africa	Base case	49·3	0·19	264
	Longer horizon	44·2	0·22	200
	Lower TPT coverage	27·4	0·10	264
	More monitoring visits	53·3	0·19	285
	No CXR screen for HHCs	34·0	0·13	263
	No TPT	48·4	0·14	338
	Secondary effects (R=0·5)	47·7	0·19	247
	Secondary effects (R=1)	46·1	0·20	230
	1HP	52·6	0·19	279
Tajikistan	Base case	14·1	0·16	91
	Longer horizon	10·3	0·18	57
	Lower TPT coverage	7·8	0·09	91
	More monitoring visits	14·7	0·16	94
	No CXR screen for HHCs	7·4	0·11	69
	No TPT	14·9	0·12	123
	Secondary effects (R=0·5)	12·9	0·16	79
	Secondary effects (R=1)	11·7	0·17	69
	1HP	17·4	0·16	111
Tanzania	Base case	18·5	0·13	145
	Longer horizon	16·3	0·15	111
	Lower TPT coverage	10·3	0·07	145
	More monitoring visits	19·2	0·13	150
	No CXR screen for HHCs	10·9	0·09	123
	No TPT	17·1	0·10	170
	Secondary effects (R=0·5)	17·7	0·13	131
	Secondary effects (R=1)	16·8	0·14	119
	1HP	22·0	0·13	170
Thailand	Base case	34·4	0·27	128
	Longer horizon	32·3	0·32	100
	Lower TPT coverage	19·1	0·15	128
	More monitoring visits	37·5	0·27	140
	No CXR screen for HHCs	28·0	0·18	152
	No TPT	30·3	0·21	144
	Secondary effects (R=0·5)	33·5	0·27	123
	Secondary effects (R=1)	32·7	0·28	118
	1HP	37·7	0·27	140
Timor-Leste	Base case	17·9	0·18	99
	Longer horizon	14·8	0·21	69
	Lower TPT coverage	10·0	0·10	99
	More monitoring visits	18·9	0·18	105
	No CXR screen for HHCs	12·3	0·12	99
	No TPT	16·6	0·14	119
	Secondary effects (R=0·5)	17·1	0·19	91
	Secondary effects (R=1)	16·2	0·20	83
	1HP	21·3	0·18	117
Uganda	Base case	16·3	0·12	136
	Longer horizon	14·5	0·14	104
	Lower TPT coverage	9·1	0·07	136
	More monitoring visits	16·9	0·12	141
	No CXR screen for HHCs	9·4	0·08	113
	No TPT	14·4	0·09	158
	Secondary effects (R=0·5)	15·8	0·13	123
	Secondary effects (R=1)	15·2	0·14	112

Country	Scenario	Inc. Disc. Costs (USD per person)	Inc. Disc. DALYs (per person)	ICER
	1HP	19·7	0·12	163
Zambia	Base case	23·1	0·23	103
	Longer horizon	19·5	0·27	71
	Lower TPT coverage	12·9	0·13	103
	More monitoring visits	23·7	0·23	105
	No CXR screen for HHCs	13·8	0·15	89
	No TPT	21·6	0·18	122
	Secondary effects (R=0·5)	22·1	0·23	95
	Secondary effects (R=1)	21·0	0·24	88
	1HP	26·6	0·23	117
Zimbabwe	Base case	24·8	0·26	95
	Longer horizon	21·7	0·32	67
	Lower TPT coverage	13·8	0·15	95
	More monitoring visits	27·0	0·26	103
	No CXR screen for HHCs	15·5	0·18	87
	No TPT	21·9	0·21	106
	Secondary effects (R=0·5)	24·0	0·27	89
	Secondary effects (R=1)	23·2	0·28	84
	1HP	28·1	0·26	106

Table shows the discounted disability adjusted life years averted (DALYs), incremental discounted costs (in 2020 USD), and incremental cost-effectiveness ratios of TPT for contacts aged 5-14 years vs. no TPT for contacts aged 5-14 years, by country and for 8 different sensitivity analyses (compared to the base case).

Appendix Table 20: Sensitivity analysis results for contacts over 15 years

Country	Scenario	Inc. Disc. Costs (USD per person)	Inc. Disc. DALYs (per person)	ICER
Bangladesh	Base case	14·2	0·04	345
	Longer horizon	13·5	0·05	280
	Lower TPT coverage	7·9	0·02	345
	More monitoring visits	15·1	0·04	368
	No CXR screen for HHCs	10·7	0·03	369
	No TPT	10·2	0·02	416
	Secondary effects (R=0·5)	13·8	0·05	291
	Secondary effects (R=1)	13·3	0·05	249
	1HP	17·2	0·04	413
Brazil	Base case	11·9	0·03	376
	Longer horizon	9·4	0·04	245
	Lower TPT coverage	6·6	0·02	376
	More monitoring visits	12·9	0·03	406
	No CXR screen for HHCs	7·1	0·02	296
	No TPT	11·9	0·02	750
	Secondary effects (R=0·5)	10·7	0·04	277
	Secondary effects (R=1)	9·4	0·05	208
	1HP	14·8	0·03	457
Burundi	Base case	11·6	0·04	265
	Longer horizon	10·2	0·05	196
	Lower TPT coverage	6·4	0·02	265
	More monitoring visits	11·8	0·04	271
	No CXR screen for HHCs	5·5	0·03	180
	No TPT	8·8	0·03	337
	Secondary effects (R=0·5)	10·9	0·05	219
	Secondary effects (R=1)	10·3	0·06	182
	1HP	14·7	0·04	331
Cambodia	Base case	9·7	0·05	184
	Longer horizon	5·8	0·06	90
	Lower TPT coverage	5·4	0·03	184
	More monitoring visits	10·7	0·05	204
	No CXR screen for HHCs	4·5	0·04	119
	No TPT	11·7	0·03	386
	Secondary effects (R=0·5)	7·9	0·06	132
	Secondary effects (R=1)	6·1	0·07	91
	1HP	12·5	0·05	234
Congo DRC	Base case	14·0	0·03	407
	Longer horizon	13·5	0·04	334
	Lower TPT coverage	7·8	0·02	407
	More monitoring visits	14·4	0·03	419
	No CXR screen for HHCs	8·2	0·02	336
	No TPT	9·4	0·02	467
	Secondary effects (R=0·5)	13·7	0·04	341
	Secondary effects (R=1)	13·4	0·05	291
	1HP	17·1	0·03	491
Eswatini	Base case	22·6	0·05	476
	Longer horizon	8·5	0·06	149
	Lower TPT coverage	12·5	0·03	476
	More monitoring visits	24·7	0·05	520
	No CXR screen for HHCs	4·3	0·03	126
	No TPT	42·8	0·03	1548
	Secondary effects (R=0·5)	16·9	0·05	315
	Secondary effects (R=1)	11·3	0·06	188
	1HP	24·7	0·05	512
Ethiopia	Base case	13·6	0·02	550
	Longer horizon	11·9	0·03	409
	Lower TPT coverage	7·6	0·01	550
	More monitoring visits	14·3	0·02	575
	No CXR screen for HHCs	6·9	0·02	382

Country	Scenario	Inc. Disc. Costs (USD per person)	Inc. Disc. DALYs (per person)	ICER
	No TPT	12·3	0·01	903
	Secondary effects (R=0·5)	12·5	0·03	417
	Secondary effects (R=1)	11·4	0·04	324
	1HP	16·7	0·03	660
Ghana	Base case	25·0	0·10	258
	Longer horizon	21·2	0·13	163
	Lower TPT coverage	13·9	0·05	258
	More monitoring visits	25·6	0·10	264
	No CXR screen for HHCs	14·4	0·07	210
	No TPT	24·2	0·06	420
	Secondary effects (R=0·5)	24·3	0·11	227
	Secondary effects (R=1)	23·5	0·12	201
	1HP	28·0	0·10	284
Haiti	Base case	8·4	0·04	201
	Longer horizon	6·1	0·05	122
	Lower TPT coverage	4·6	0·02	201
	More monitoring visits	9·1	0·04	219
	No CXR screen for HHCs	3·7	0·03	125
	No TPT	7·7	0·02	325
	Secondary effects (R=0·5)	7·2	0·05	150
	Secondary effects (R=1)	6·1	0·05	111
	1HP	11·3	0·04	267
India	Base case	14·3	0·05	292
	Longer horizon	13·3	0·06	225
	Lower TPT coverage	8·0	0·03	292
	More monitoring visits	15·7	0·05	319
	No CXR screen for HHCs	10·7	0·04	302
	No TPT	10·5	0·03	374
	Secondary effects (R=0·5)	13·9	0·06	244
	Secondary effects (R=1)	13·4	0·06	208
	1HP	17·4	0·05	347
Indonesia	Base case	20·9	0·09	246
	Longer horizon	19·9	0·11	184
	Lower TPT coverage	11·6	0·05	246
	More monitoring visits	22·4	0·09	263
	No CXR screen for HHCs	16·7	0·06	275
	No TPT	16·3	0·05	327
	Secondary effects (R=0·5)	20·7	0·09	220
	Secondary effects (R=1)	20·4	0·10	199
	1HP	24·0	0·09	277
Kenya	Base case	19·6	0·05	414
	Longer horizon	16·8	0·06	292
	Lower TPT coverage	10·9	0·03	414
	More monitoring visits	20·7	0·05	439
	No CXR screen for HHCs	11·0	0·03	324
	No TPT	19·0	0·03	694
	Secondary effects (R=0·5)	18·4	0·05	339
	Secondary effects (R=1)	17·3	0·06	281
	1HP	22·6	0·05	469
Lesotho	Base case	18·4	0·08	234
	Longer horizon	13·5	0·10	133
	Lower TPT coverage	10·2	0·04	234
	More monitoring visits	19·1	0·08	243
	No CXR screen for HHCs	8·4	0·06	148
	No TPT	19·3	0·04	429
	Secondary effects (R=0·5)	17·2	0·09	200
	Secondary effects (R=1)	16·1	0·09	171
	1HP	21·2	0·08	265
Liberia	Base case	13·8	0·07	199
	Longer horizon	12·4	0·09	141
	Lower TPT coverage	7·6	0·04	199

Country	Scenario	Inc. Disc. Costs (USD per person)	Inc. Disc. DALYs (per person)	ICER
	More monitoring visits	14·0	0·07	203
	No CXR screen for HHCs	7·4	0·05	150
	No TPT	10·5	0·04	256
	Secondary effects (R=0·5)	13·3	0·08	172
	Secondary effects (R=1)	12·9	0·09	150
	1HP	16·9	0·07	239
Malawi	Base case	12·3	0·04	295
	Longer horizon	9·9	0·05	198
	Lower TPT coverage	6·8	0·02	295
	More monitoring visits	12·8	0·04	306
	No CXR screen for HHCs	5·3	0·03	180
	No TPT	11·6	0·02	480
	Secondary effects (R=0·5)	11·2	0·05	230
	Secondary effects (R=1)	10·1	0·06	182
	1HP	15·3	0·04	361
Mongolia	Base case	27·6	0·12	239
	Longer horizon	15·1	0·16	96
	Lower TPT coverage	15·4	0·06	239
	More monitoring visits	29·1	0·12	252
	No CXR screen for HHCs	11·9	0·08	147
	No TPT	36·2	0·07	520
	Secondary effects (R=0·5)	25·1	0·12	202
	Secondary effects (R=1)	22·5	0·13	169
	1HP	30·2	0·12	258
Mozambique	Base case	12·5	0·01	837
	Longer horizon	11·7	0·02	675
	Lower TPT coverage	6·9	0·01	837
	More monitoring visits	13·0	0·01	871
	No CXR screen for HHCs	6·7	0·01	604
	No TPT	9·1	0·01	1179
	Secondary effects (R=0·5)	11·9	0·02	592
	Secondary effects (R=1)	11·3	0·03	448
	1HP	15·6	0·02	1025
Namibia	Base case	31·4	0·05	688
	Longer horizon	22·3	0·06	402
	Lower TPT coverage	17·5	0·03	688
	More monitoring visits	34·2	0·05	750
	No CXR screen for HHCs	15·4	0·03	473
	No TPT	42·2	0·03	1590
	Secondary effects (R=0·5)	27·5	0·05	529
	Secondary effects (R=1)	23·6	0·06	405
	1HP	33·9	0·05	730
Pakistan	Base case	13·0	0·07	181
	Longer horizon	12·5	0·09	138
	Lower TPT coverage	7·2	0·04	181
	More monitoring visits	13·8	0·07	192
	No CXR screen for HHCs	9·8	0·05	192
	No TPT	8·1	0·04	191
	Secondary effects (R=0·5)	12·8	0·08	161
	Secondary effects (R=1)	12·7	0·09	144
	1HP	16·1	0·07	221
Rwanda	Base case	7·8	0·03	289
	Longer horizon	2·8	0·03	88
	Lower TPT coverage	4·4	0·02	289
	More monitoring visits	8·4	0·03	311
	No CXR screen for HHCs	-0·5	0·02	-
	No TPT	14·7	0·02	967
	Secondary effects (R=0·5)	4·8	0·03	146
	Secondary effects (R=1)	1·7	0·04	44
	1HP	10·5	0·03	381
Somalia	Base case	13·8	0·08	184

Country	Scenario	Inc. Disc. Costs (USD per person)	Inc. Disc. DALYs (per person)	ICER
	Longer horizon	11·7	0·10	120
	Lower TPT coverage	7·7	0·04	184
	More monitoring visits	14·7	0·08	196
	No CXR screen for HHCs	6·9	0·05	129
	No TPT	11·0	0·04	247
	Secondary effects (R=0·5)	13·2	0·08	158
	Secondary effects (R=1)	12·7	0·09	136
	1HP	16·9	0·08	221
South Africa	Base case	41·0	0·04	1098
	Longer horizon	37·7	0·04	841
	Lower TPT coverage	22·8	0·02	1098
	More monitoring visits	45·2	0·04	1210
	No CXR screen for HHCs	27·9	0·03	1025
	No TPT	40·4	0·02	1977
	Secondary effects (R=0·5)	39·4	0·04	898
	Secondary effects (R=1)	37·9	0·05	750
	1HP	43·9	0·04	1154
Tajikistan	Base case	10·5	0·07	155
	Longer horizon	7·4	0·08	87
	Lower TPT coverage	5·9	0·04	155
	More monitoring visits	11·1	0·07	164
	No CXR screen for HHCs	4·6	0·05	96
	No TPT	10·2	0·04	251
	Secondary effects (R=0·5)	9·4	0·08	125
	Secondary effects (R=1)	8·3	0·08	101
	1HP	13·5	0·07	195
Tanzania	Base case	16·0	0·04	404
	Longer horizon	14·4	0·05	307
	Lower TPT coverage	8·9	0·02	404
	More monitoring visits	16·7	0·04	422
	No CXR screen for HHCs	8·9	0·03	317
	No TPT	13·7	0·02	582
	Secondary effects (R=0·5)	15·2	0·05	329
	Secondary effects (R=1)	14·3	0·05	273
	1HP	19·1	0·04	473
Thailand	Base case	29·5	0·02	1637
	Longer horizon	28·5	0·02	1372
	Lower TPT coverage	16·4	0·01	1637
	More monitoring visits	32·8	0·02	1823
	No CXR screen for HHCs	24·6	0·01	1827
	No TPT	25·3	0·01	2728
	Secondary effects (R=0·5)	28·7	0·02	1252
	Secondary effects (R=1)	27·8	0·03	1002
	1HP	32·5	0·02	1768
Timor-Leste	Base case	13·6	0·06	225
	Longer horizon	11·5	0·08	152
	Lower TPT coverage	7·6	0·03	225
	More monitoring visits	14·6	0·06	241
	No CXR screen for HHCs	9·2	0·04	212
	No TPT	11·7	0·04	329
	Secondary effects (R=0·5)	12·8	0·07	189
	Secondary effects (R=1)	12·0	0·07	160
	1HP	16·6	0·06	269
Uganda	Base case	15·4	0·06	261
	Longer horizon	13·9	0·07	186
	Lower TPT coverage	8·6	0·03	261
	More monitoring visits	16·0	0·06	272
	No CXR screen for HHCs	8·6	0·04	204
	No TPT	12·3	0·03	359
	Secondary effects (R=0·5)	14·9	0·07	223
	Secondary effects (R=1)	14·4	0·07	193

Country	Scenario	Inc. Disc. Costs (USD per person)	Inc. Disc. DALYs (per person)	ICER
	1HP	18·5	0·06	309
Zambia	Base case	15·6	0·03	531
	Longer horizon	13·7	0·03	401
	Lower TPT coverage	8·6	0·02	531
	More monitoring visits	16·1	0·03	551
	No CXR screen for HHCs	8·3	0·02	396
	No TPT	14·2	0·02	847
	Secondary effects (R=0·5)	14·5	0·04	408
	Secondary effects (R=1)	13·5	0·04	321
	1HP	18·6	0·03	624
Zimbabwe	Base case	18·6	0·05	371
	Longer horizon	16·6	0·06	270
	Lower TPT coverage	10·3	0·03	371
	More monitoring visits	21·0	0·05	418
	No CXR screen for HHCs	10·9	0·04	304
	No TPT	15·6	0·03	538
	Secondary effects (R=0·5)	17·8	0·06	310
	Secondary effects (R=1)	17·1	0·06	264
	1HP	21·7	0·05	424

Table shows the discounted disability adjusted life years averted (DALYs), incremental discounted costs (in 2020 USD), and incremental cost-effectiveness ratios of TPT for contacts aged 15 years or older vs. no TPT for contacts 15 or older, by country and for 8 different sensitivity analyses (compared to the base case).

Appendix Table 21: Sensitivity analysis results for people living with HIV/AIDS

Country	Scenario	Inc. Disc. Costs (USD per person)	Inc. Disc. DALYs (per person)	ICER
Bangladesh	Base case	14·0	0·019	725
	Longer horizon	13·1	0·041	323
	Lower TPT coverage	12·6	0·014	911
	Lower ART turnover	14·0	0·018	797
	More monitoring visits	15·0	0·019	780
	Secondary effects (R=0·5)	13·5	0·026	520
	Secondary effects (R=1)	13·0	0·033	399
	1HP	21·3	0·020	1060
Brazil	Base case	12·9	0·012	1069
	Longer horizon	10·0	0·026	390
	Lower TPT coverage	11·7	0·008	1444
	Lower ART turnover	12·7	0·011	1117
	More monitoring visits	14·0	0·012	1159
	Secondary effects (R=0·5)	12·3	0·015	798
	Secondary effects (R=1)	11·7	0·019	624
	1HP	20·0	0·013	1586
Burundi	Base case	11·8	0·013	912
	Longer horizon	9·7	0·030	328
	Lower TPT coverage	10·7	0·009	1172
	Lower ART turnover	11·8	0·011	1047
	More monitoring visits	12·1	0·013	936
	Secondary effects (R=0·5)	11·2	0·018	615
	Secondary effects (R=1)	10·7	0·024	453
	1HP	19·1	0·013	1415
Cambodia	Base case	-0·6	0·034	-
	Longer horizon	-20·4	0·079	-
	Lower TPT coverage	2·2	0·024	92
	Lower ART turnover	-1·0	0·030	-
	More monitoring visits	0·5	0·034	15
	Secondary effects (R=0·5)	-4·5	0·049	-
	Secondary effects (R=1)	-8·4	0·065	-
	1HP	5·9	0·035	165
Congo DRC	Base case	12·9	0·022	592
	Longer horizon	13·0	0·045	286
	Lower TPT coverage	11·5	0·015	757
	Lower ART turnover	12·7	0·020	639
	More monitoring visits	13·4	0·022	612
	Secondary effects (R=0·5)	12·6	0·028	446
	Secondary effects (R=1)	12·3	0·035	355
	1HP	20·1	0·023	884
Eswatini	Base case	-13·8	0·019	-
	Longer horizon	-56·3	0·044	-
	Lower TPT coverage	-5·9	0·013	-
	Lower ART turnover	-14·0	0·016	-
	More monitoring visits	-11·4	0·019	-
	Secondary effects (R=0·5)	-20·5	0·026	-
	Secondary effects (R=1)	-27·2	0·034	-
	1HP	-7·9	0·020	-
Ethiopia	Base case	10·9	0·016	674
	Longer horizon	7·3	0·036	203
	Lower TPT coverage	10·2	0·011	908
	Lower ART turnover	10·8	0·014	751
	More monitoring visits	11·6	0·016	716
	Secondary effects (R=0·5)	9·9	0·021	471
	Secondary effects (R=1)	8·9	0·026	344
	1HP	18·1	0·017	1069
Ghana	Base case	6·7	0·020	334
	Longer horizon	-2·5	0·046	-
	Lower TPT coverage	7·2	0·014	535

Country	Scenario	Inc. Disc. Costs (USD per person)	Inc. Disc. DALYs (per person)	ICER
	Lower ART turnover	6·3	0·018	359
	More monitoring visits	7·4	0·020	367
	Secondary effects (R=0·5)	5·9	0·030	195
	Secondary effects (R=1)	5·1	0·040	126
	1HP	13·5	0·021	642
Haiti	Base case	8·7	0·019	449
	Longer horizon	2·1	0·046	46
	Lower TPT coverage	8·4	0·012	683
	Lower ART turnover	8·1	0·017	489
	More monitoring visits	9·5	0·019	491
	Secondary effects (R=0·5)	7·5	0·026	292
	Secondary effects (R=1)	6·4	0·032	198
	1HP	15·3	0·020	762
India	Base case	14·4	0·020	733
	Longer horizon	12·9	0·043	296
	Lower TPT coverage	12·9	0·013	960
	Lower ART turnover	14·1	0·017	810
	More monitoring visits	15·9	0·020	810
	Secondary effects (R=0·5)	13·9	0·028	502
	Secondary effects (R=1)	13·4	0·036	376
	1HP	21·5	0·020	1052
Indonesia	Base case	13·0	0·037	349
	Longer horizon	11·0	0·086	127
	Lower TPT coverage	11·6	0·024	478
	Lower ART turnover	12·2	0·032	383
	More monitoring visits	14·6	0·037	392
	Secondary effects (R=0·5)	12·5	0·053	235
	Secondary effects (R=1)	12·0	0·069	174
	1HP	19·7	0·039	507
Kenya	Base case	13·2	0·008	1624
	Longer horizon	10·3	0·019	549
	Lower TPT coverage	12·1	0·006	2144
	Lower ART turnover	13·2	0·007	1849
	More monitoring visits	14·6	0·008	1785
	Secondary effects (R=0·5)	12·7	0·012	1093
	Secondary effects (R=1)	12·1	0·015	806
	1HP	20·5	0·009	2408
Lesotho	Base case	3·7	0·023	158
	Longer horizon	-9·4	0·051	-
	Lower TPT coverage	5·2	0·016	324
	Lower ART turnover	3·2	0·021	156
	More monitoring visits	4·5	0·023	194
	Secondary effects (R=0·5)	2·2	0·034	64
	Secondary effects (R=1)	0·7	0·044	15
	1HP	10·4	0·024	432
Liberia	Base case	11·1	0·018	619
	Longer horizon	9·1	0·040	231
	Lower TPT coverage	10·1	0·012	829
	Lower ART turnover	10·9	0·016	679
	More monitoring visits	11·4	0·018	635
	Secondary effects (R=0·5)	10·7	0·025	423
	Secondary effects (R=1)	10·4	0·033	315
	1HP	18·2	0·019	968
Malawi	Base case	10·0	0·013	777
	Longer horizon	5·6	0·031	180
	Lower TPT coverage	9·4	0·009	1096
	Lower ART turnover	9·7	0·011	885
	More monitoring visits	10·5	0·013	816
	Secondary effects (R=0·5)	9·2	0·018	502
	Secondary effects (R=1)	8·4	0·024	352
	1HP	17·0	0·013	1260

Country	Scenario	Inc. Disc. Costs (USD per person)	Inc. Disc. DALYs (per person)	ICER
Mongolia	Base case	-22·0	0·028	-
	Longer horizon	-71·0	0·063	-
	Lower TPT coverage	-11·6	0·018	-
	Lower ART turnover	-23·1	0·024	-
	More monitoring visits	-20·4	0·028	-
	Secondary effects (R=0·5)	-26·0	0·043	-
	Secondary effects (R=1)	-29·9	0·057	-
	1HP	-16·5	0·029	-
Mozambique	Base case	10·9	0·024	463
	Longer horizon	8·6	0·061	143
	Lower TPT coverage	9·7	0·015	635
	Lower ART turnover	10·4	0·020	515
	More monitoring visits	11·5	0·024	486
	Secondary effects (R=0·5)	10·1	0·031	330
	Secondary effects (R=1)	9·3	0·038	247
	1HP	17·7	0·025	719
Namibia	Base case	-9·2	0·027	-
	Longer horizon	-51·2	0·066	-
	Lower TPT coverage	-1·8	0·017	-
	Lower ART turnover	-9·5	0·023	-
	More monitoring visits	-6·2	0·027	-
	Secondary effects (R=0·5)	-15·0	0·036	-
	Secondary effects (R=1)	-20·8	0·046	-
	1HP	-3·5	0·028	-
Pakistan	Base case	12·5	0·020	618
	Longer horizon	12·5	0·061	206
	Lower TPT coverage	10·2	0·012	827
	Lower ART turnover	11·9	0·017	689
	More monitoring visits	13·3	0·020	657
	Secondary effects (R=0·5)	12·3	0·028	443
	Secondary effects (R=1)	12·2	0·035	343
	1HP	18·9	0·021	896
Rwanda	Base case	11·9	0·004	2866
	Longer horizon	8·6	0·009	941
	Lower TPT coverage	11·0	0·003	3755
	Lower ART turnover	12·1	0·004	3256
	More monitoring visits	12·6	0·004	3027
	Secondary effects (R=0·5)	11·1	0·005	2028
	Secondary effects (R=1)	10·4	0·007	1520
	1HP	19·2	0·004	4447
Somalia	Base case	10·7	0·016	685
	Longer horizon	6·1	0·043	142
	Lower TPT coverage	9·6	0·010	965
	Lower ART turnover	10·4	0·013	769
	More monitoring visits	11·7	0·016	745
	Secondary effects (R=0·5)	10·3	0·023	454
	Secondary effects (R=1)	9·8	0·030	331
	1HP	17·4	0·016	1061
South Africa	Base case	17·4	0·022	802
	Longer horizon	7·8	0·051	153
	Lower TPT coverage	16·6	0·015	1133
	Lower ART turnover	16·9	0·019	893
	More monitoring visits	22·0	0·022	1016
	Secondary effects (R=0·5)	15·5	0·030	523
	Secondary effects (R=1)	13·6	0·038	362
	1HP	24·3	0·023	1074
Tajikistan	Base case	9·0	0·014	627
	Longer horizon	2·8	0·033	86
	Lower TPT coverage	8·8	0·009	925
	Lower ART turnover	8·7	0·012	694
	More monitoring visits	9·7	0·014	672

Country	Scenario	Inc. Disc. Costs (USD per person)	Inc. Disc. DALYs (per person)	ICER
	Secondary effects (R=0·5)	8·2	0·020	417
	Secondary effects (R=1)	7·4	0·025	295
	1HP	15·9	0·015	1056
Tanzania	Base case	10·6	0·019	570
	Longer horizon	6·2	0·046	135
	Lower TPT coverage	9·8	0·012	789
	Lower ART turnover	10·3	0·016	651
	More monitoring visits	11·3	0·019	613
	Secondary effects (R=0·5)	9·6	0·026	371
	Secondary effects (R=1)	8·6	0·033	260
	1HP	17·5	0·019	906
Thailand	Base case	21·0	0·022	949
	Longer horizon	18·9	0·047	398
	Lower TPT coverage	19·1	0·016	1202
	Lower ART turnover	21·1	0·020	1063
	More monitoring visits	25·0	0·022	1127
	Secondary effects (R=0·5)	20·0	0·028	704
	Secondary effects (R=1)	18·9	0·035	547
	1HP	28·4	0·023	1230
Timor-Leste	Base case	6·5	0·031	207
	Longer horizon	-4·9	0·089	-
	Lower TPT coverage	6·8	0·020	338
	Lower ART turnover	6·1	0·027	225
	More monitoring visits	7·5	0·031	239
	Secondary effects (R=0·5)	5·1	0·044	117
	Secondary effects (R=1)	3·7	0·056	66
	1HP	12·8	0·033	390
Uganda	Base case	13·3	0·009	1438
	Longer horizon	12·1	0·021	584
	Lower TPT coverage	12·0	0·007	1832
	Lower ART turnover	13·4	0·008	1634
	More monitoring visits	14·0	0·009	1512
	Secondary effects (R=0·5)	13·0	0·014	955
	Secondary effects (R=1)	12·7	0·018	706
	1HP	20·7	0·010	2139
Zambia	Base case	9·3	0·020	474
	Longer horizon	3·5	0·049	71
	Lower TPT coverage	8·9	0·013	668
	Lower ART turnover	9·2	0·017	538
	More monitoring visits	9·9	0·020	505
	Secondary effects (R=0·5)	8·1	0·027	296
	Secondary effects (R=1)	6·8	0·035	196
	1HP	16·2	0·021	791
Zimbabwe	Base case	17·5	0·013	1320
	Longer horizon	14·5	0·031	474
	Lower TPT coverage	15·8	0·009	1734
	Lower ART turnover	17·3	0·011	1510
	More monitoring visits	20·1	0·013	1521
	Secondary effects (R=0·5)	16·8	0·019	881
	Secondary effects (R=1)	16·2	0·025	649
	1HP	24·7	0·014	1787

Table shows the discounted disability adjusted life years averted (DALYs) per person, incremental discounted costs (in 2020 USD) per person, and incremental cost-effectiveness ratios of TPT for people living with HIV/AIDS vs. no TPT for people living with HIV/AIDS, by country and for 7 different sensitivity analyses (compared to the base case). A dash (“-“) in the ICER column indicates TPT was considered cost-saving in this analysis.
